# Feline calicivirus strain 2280 p30 antagonizes type I interferon-mediated antiviral innate immunity through directly degrading IFNAR1 mRNA

**DOI:** 10.1371/journal.ppat.1008944

**Published:** 2020-10-19

**Authors:** Jin Tian, Hongtao Kang, Jiapei Huang, Zhijie Li, Yudi Pan, Yin Li, Si Chen, Jikai Zhang, Hang Yin, Liandong Qu

**Affiliations:** Division of Zoonosis of Natural Foci, State Key Laboratory of Veterinary Biotechnology, Harbin Veterinary Research Institute, Chinese Academy of Agricultural Sciences, Harbin, P. R. China; University of Michigan, USA, UNITED STATES

## Abstract

Feline calicivirus (FCV) belongs to the *Caliciviridae*, which comprises small RNA viruses of both medical and veterinary importance. Once infection has occurred, FCV can persist in the cat population, but the molecular mechanism of how it escapes the innate immune response is still unknown. In this study, we found FCV strain 2280 to be relatively resistant to treatment with IFN-β. FCV 2280 infection inhibited IFN-induced activation of the ISRE (Interferon-stimulated response element) promoter and transcription of ISGs (Interferon-stimulated genes). The mechanistic analysis showed that the expression of IFNAR1, but not IFNAR2, was markedly reduced in FCV 2280-infected cells by inducing the degradation of IFNAR1 mRNA, which inhibited the phosphorylation of downstream adaptors. Further, overexpression of the FCV 2280 nonstructural protein p30, but not p30 of the attenuated strain F9, downregulated the expression of IFNAR1 mRNA. His-p30 fusion proteins were produced in *Escherichia coli* and purified, and an *in vitro* digestion assay was performed. The results showed that 2280 His-p30 could directly degrade IFNAR1 RNA but not IFNAR2 RNA. Moreover, the 5’UTR of IFNAR1 mRNA renders it directly susceptible to cleavage by 2280 p30. Next, we constructed two chimeric viruses: rFCV 2280-F9 p30 and rFCV F9-2280 p30. Compared to infection with the parental virus, rFCV 2280-F9 p30 infection displayed attenuated activities in reducing the level of IFNAR1 and inhibiting the phosphorylation of STAT1 and STAT2, whereas rFCV F9-2280 p30 displayed enhanced activities. Animal experiments showed that the virulence of rFCV 2280-F9 p30 infection was attenuated but that the virulence of rFCV F9-2280 p30 was increased compared to that of the parental viruses. Collectively, these data show that FCV 2280 p30 could directly and selectively degrade IFNAR1 mRNA, thus blocking the type I interferon-induced activation of the JAK-STAT signalling pathway, which may contribute to the pathogenesis of FCV infection.

## Author summary

Vaccination against FCV has been available for many years and has effectively reduced the incidence of clinical disease. However, vaccines cannot prevent infection, and vaccinated cats can still become persistently infected by FCV, suggesting that FCV has evolved several strategies for counteracting various components of the innate and adaptive immune systems. Here, we show that FCV strain 2280 is resistant to the antiviral effect of IFN. The molecular mechanism by which this occurs is that FCV 2280 infection blocks the JAK-STAT pathway through promoting the degradation of IFNAR1 mRNA by FCV p30 protein. An *in vitro* degradation assay demonstrated that 2280 p30, but not p30 of the vaccine strain F9, could directly and selectively decay IFNAR1 RNA. The exchange of p30 between 2280 and F9 strains using a reverse genetic system also showed that 2280 p30 is a key factor that contributes to the resistance to IFN and enhances virulence. Our findings reveal a new mechanism evolved by FCV to circumvent the host antiviral response.

## Introduction

Feline calicivirus (FCV) is a non-enveloped RNA virus with a single-stranded positive-sense RNA genome approximately 7.5 kb in size, which is enclosed in an icosahedral capsid with a diameter of 27–40 nm [1, 2]. FCV is a common pathogen of cats, with a widespread distribution in the cat population. FCV infection is moderate and not fatal in adult cats and could lead to pneumonia or severe upper respiratory tract disease in some young kittens cases [3]. More recently, highly virulent strains of FCV have emerged that are associated with high mortality and morbidity and a new range of clinical features (FCV-associated virulent systemic disease; VSD) [1, 4, 5].

FCV belongs to the genus *Vesivirus* in the *Caliciviridae*, which contains small RNA viruses of both medical and veterinary importance. The *Caliciviridae* has eleven well-defined members [6] [1]. Among these, seven members (*Lagovirus*, *Norovirus*, *Nebovirus*, *Recovirus*, *Sapovirus*, *Valovirus* and *Vesivirus*) infect mammals, two members (*Bavovirus* and *Nacovirus*) infect birds, and two members (*Minovirus* and *Salovirus*) infect fishes. Most caliciviruses are difficult to cultivate *in vitro*, and no highly efficient, easy-to-use cell culture model is available for human norovirus (HuNoV); however, recent studies indicate that limited HuNoV replication can occur in immortalized B cells [7–9] and stem cell-derived enteroids [10]. Additionally, due to the technical limitations of these experimental systems, no perfect animal model is widely available for the research of virus biology [11]; however, zebrafish larvae support the replication of HuNoV and may function as a good model to evaluate antiviral reagents [12]. This problem severely hinders the investigation of the calicivirus life cycle, and the function of some viral proteins is not well-known [13]. However, murine norovirus (MNV) (genus *Norovirus*) and FCV have been used as excellent models to explore the calicivirus biology due to their culturability and the availability of mature animal models for virus pathogenesis [14, 15].

Alpha and beta interferons (IFN-α/β) are crucial components of the early host response against virus infection [16]. Cells respond rapidly following stimulation with IFNs via the Janus kinase-signal transducer and activator of transcription (JAK-STAT) signal transduction pathway [17]. Briefly, the JAK-STAT pathway is activated when IFNs bind to the interferon alpha and beta receptor subunit 1 (IFNAR1) and IFNAR2. The binding of IFN-α/β to its receptors activates JAK1 and tyrosine kinase 2 (Tyk2), which phosphorylate and activate the signal transducer and activator of STAT2 and STAT1. Upon phosphorylation, STAT1 and STAT2 form heterodimers and then associate with interferon regulatory factor 9 (IRF-9) to form a transcription factor complex, named IFN-stimulated gene factor 3 (ISGF-3). The heterotrimer complexes translocate into the nucleus and bind to the IFN-stimulated response elements to induce the coordinated upregulation of hundreds of ISGs that orchestrate an antiviral state in the cell [18, 19].

To circumvent host antiviral innate immunity, viruses have evolved various strategies to prevent the activation of antiviral effectors in host cells, especially by minimizing IFN production and IFN-related antiviral protein expression [20–24]. A number of viruses have been found to impair the activity of the JAK-STAT signalling pathway to replicate successfully in the host [25]. Some viruses can block the function of one of the adaptor proteins required in the IFN signalling pathway [26–31]. Some viruses counteract this pathway through reducing the levels of IFNAR1/2 or inhibiting the interaction between IFNAR1/2 and adaptor proteins [32, 33]. Another class of viruses can globally impair cellular gene expression to reduce activation of immune pathways [34–38]. However, no one has described a similar process for caliciviruses.

FCV can replicate quickly and produce cytopathic effects in cells [39]. Moreover, reinfection with a variant of the same strain or with a different strain leads to a gradual increase in the diversity of FCV [40], which may contributes to widespread and persistent subclinical infection with 15–25% of cats being subclinical carriers [41]. Most cats recover from clinical disease, but some would become viral carrier that sheds virus particles into the environment. How does this virus escape the host innate antiviral immunity, which leads to virus reinfection in cats? As yet, the detailed mechanism of how FCV manipulates host innate antiviral immunity is unknown. In this study, we show that FCV strain 2280 is resistant to IFN treatment. Further, FCV 2280 infection contributes to degradation of IFNAR1 mRNA. Moreover, virus p30 protein is a key factor that induces degradation of IFNAR1 mRNA, although p30 of another vaccine strain, F9, does not affect the expression of IFNAR1 mRNA. Finally, two chimeric viruses, r2280-F9 p30 and rF9-2280 p30, were constructed, and both their anti-IFN activity and virulence in cats were evaluated. This study provides us with a better understanding of how FCV and other caliciviruses escape host innate antiviral immunity and reveals that the FCV p30 protein helps the virus to escape host innate immunity. Moreover, identification of viral factors that affect the JAK/STAT pathway would provide a strategy for creating newly attenuated vaccines by reverse genetics.

## Results

### IFN-β markedly inhibited replication of FCV strain F9 but only slightly inhibited replication of FCV strain 2280

**Fig 1:**
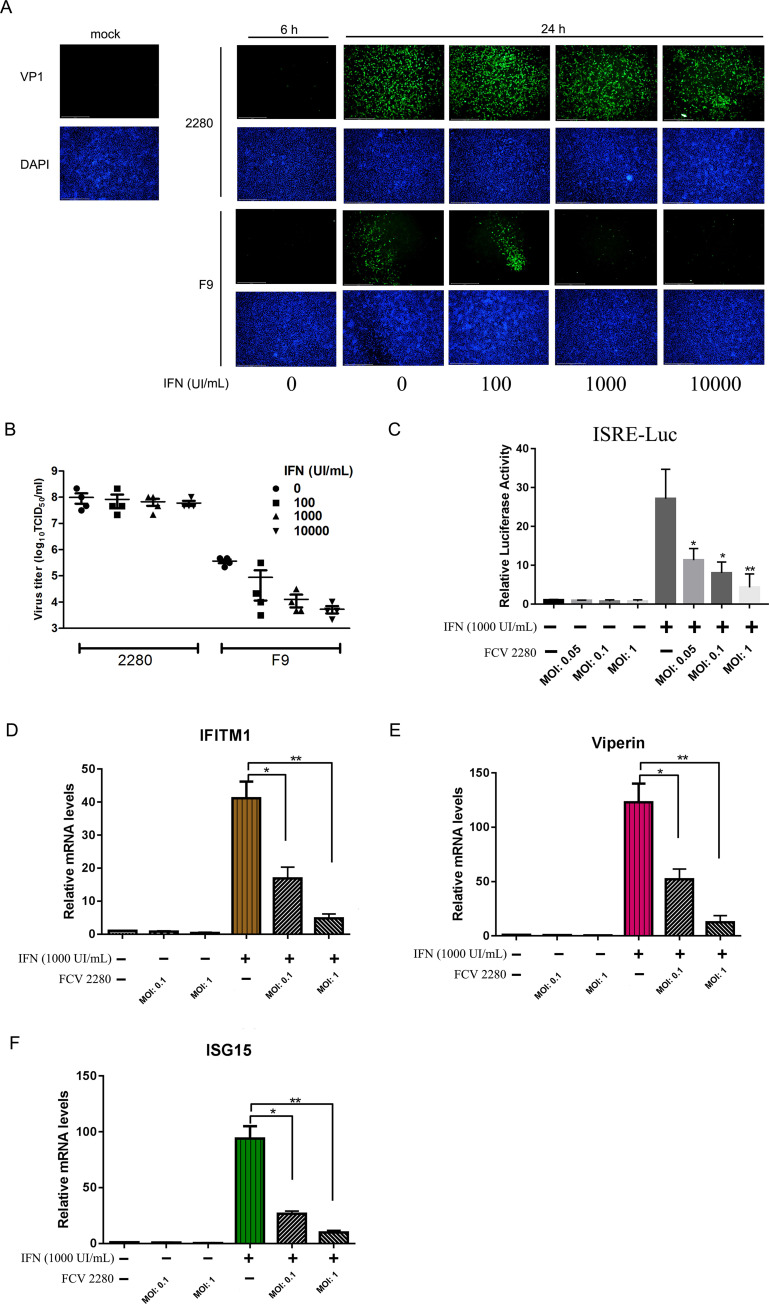
FCV 2280 infection is resistant to IFN-β. (A, B) CRFK cells were infected with FCV 2280 or F9 at an MOI of 0.01 for 6 h. Subsequently, cells were treated with different concentrations of IFN-β. At 18 h after IFN-β treatment, the cells were fixed and viral antigen was assayed by IFA using anti-VP1 antibody (A). At the same time, supernatant samples were collected, and viral titres were determined (B). (C) CRFK cells (2×10^5^) were transfected with 200 ng/well of the reporter plasmid pISRE-TA-Luc and with 20 ng/well of the pRLTK plasmid for 12 h. After transfection, the cells were infected with FCV 2280 at an MOI of 0.05, 0.1 or 1 for 8 h, then were treated with IFN-β (1000 U/mL) for 12 h. Luciferase assays were performed. (D, E, F) CRFK cells were infected with FCV 2280 at an MOI of 0.1 or 1 for 6 h, then were treated with IFN-β (1000 U/mL) for 12 h. The mRNA levels of IFITM1 (D), viperin (E) and ISG15 (F) relative to 18S rRNA was determined by qRT-PCR. The data in C, D, E, F shown represent the mean ± SD for three repeats, and the experiments were repeated three times. Differences (*p < 0.05, **p < 0.01, ***p < 0.001) between the experimental and control groups are noted.

Our previous work demonstrated that the overexpression of IFN-β at 12 hpi (hours post-infection) cannot efficiently inhibit FCV 2280 replication [42]. We performed an RNA-seq assay on the FCV strain 2280 infection at 12 hpi and found that infection led to the downregulated expression of interferon receptor 1 (IFNAR1) (S1 Table), suggesting that FCV 2280 is able to evade the host antiviral response. To examine the effects of IFN-β on FCV infection, CRFK cells were infected with FCV 2280 or F9 (MOI = 0.01) for 6 h to establish replication, and the cells were then cultured further in the presence of IFN-β with 100, 1000 or 10000 U/mL for 18 h. FCV F9 is a vaccine strain and served as a control. The antiviral effect of IFN-β was analysed. As shown in Fig 1A, IFN-β (1000 and 10000 U/mL) treatment was able to efficiently inhibit F9 infection but could not inhibit 2280 infection. To quantitatively evaluate the anti-IFN-β activity of FCV strain 2280 and F9, cellular supernatant from infected cells with the same treatment were harvested for viral titration. Low concentrations (100 and 1000 U/mL) of IFN-β were not able to significantly inhibit 2280 infection, and a high concentration (10000 U/mL) of IFN-β only slightly decreased the level of 2280 infection by 1- to 2-fold (Fig 1B). Strain F9 exhibited very high sensitivity to IFN-β, and 1000 U/mL of IFN-β was able to significantly inhibit virus infection, with 10-fold reduction (Fig 1B). These data suggest that FCV strain 2280 is resistant to IFN-β treatment.

### FCV 2280 infection can block ISRE activation and ISG expression

To systematically investigate the relationship between the type I interferon signalling pathway and FCV infection, the induction of IFN-β after FCV infection was examined at 10 hpi. FCV 2280 and F9 could not activate the IFN-β promoter (S1A Fig) or upregulate the expression of IFN-β mRNA (S1B Fig) at 10 hpi. Due to the potential anti-IFN activity of FCV 2280 infection, we evaluated whether 2280 infection could inhibit the IFN-β downstream response. CRFK cells were co-transfected with pISRE promoter-Luc and pRLTK for 12 h following FCV 2280 infection after which they were treated with IFN-β (1000 U/mL) for 12 h and luciferase assays were performed. FCV 2280 infection was able to block the IFN-β-induced ISRE activation and the effect depended on virus inoculation dose (Fig 1C). Further, the expression of three interferon-stimulated genes (ISGs), IFITM1, Viperin and ISG15, was identified by qRT-PCR. FCV 2280 infection could efficiently inhibit the IFN-induced upregulation of these three ISGs and the effect depended on virus inoculation dose, as shown in Fig 1D, 1E and 1F. To exclude that the reduction was caused by death upon infection, cell viability was examined upon 2280 infection for 18 h using trypan blue staining and CCK8 test. The results showed that the mean percentage of viable cells reached 91.65% by trypan blue staining assay (S1C Fig) and the mean ratio of viable cells reached 0.875 by CCK8 test (S1D Fig), revealing that most of cells are still live 18 h after 2280 infection with an MOI of 1.

These data indicate that FCV 2280 infection blocks activation of the interferon downstream signalling pathway.

### FCV 2280 infection downregulates IFNAR1 expression and blocks the activation of its downstream adaptor proteins

**Fig 2:**
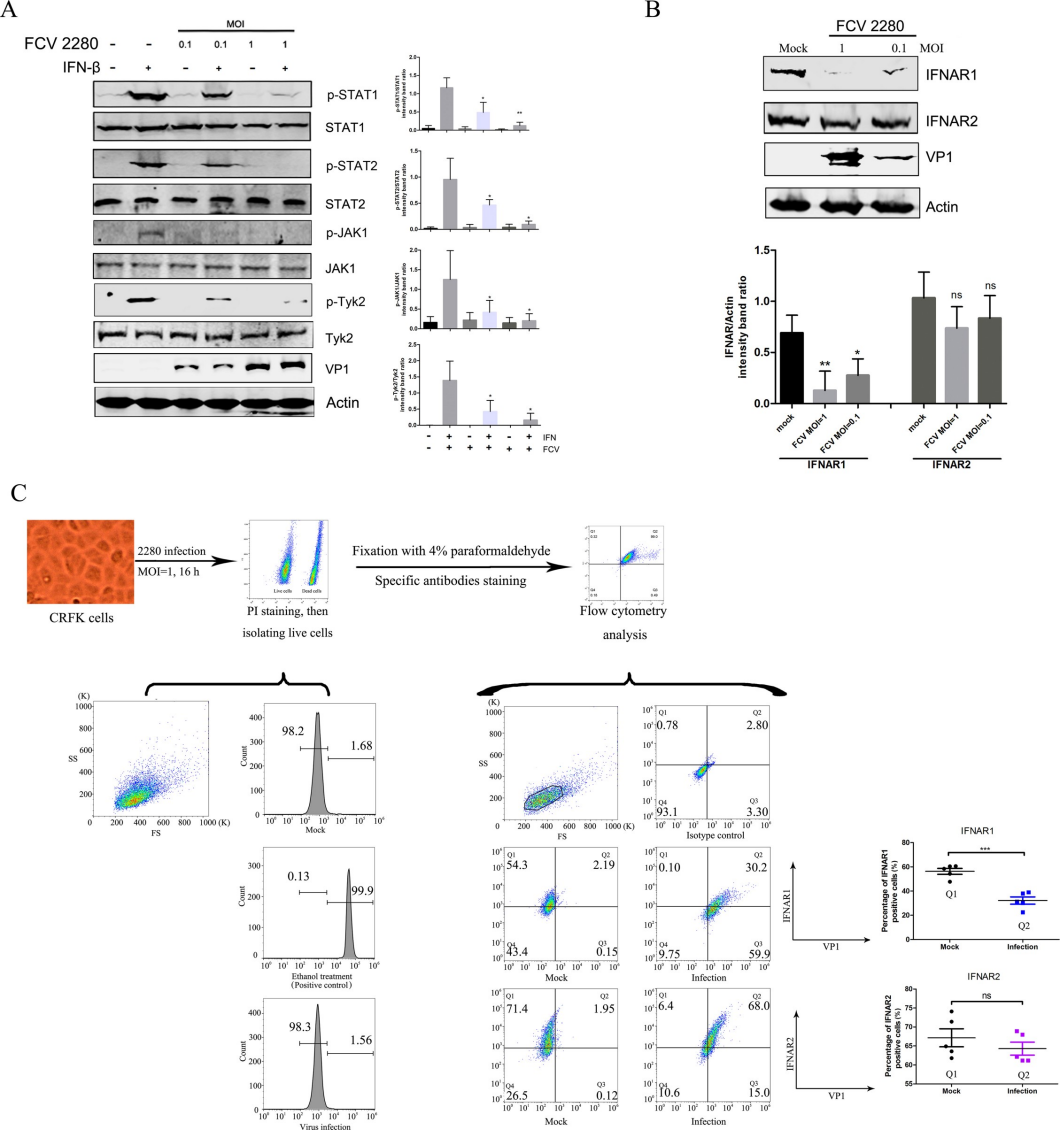
FCV 2280 infection reduces the expression of IFNAR1 and then inhibits the activation of STATs and JAKs. (A) CRFK cells were infected with FCV 2280 at an MOI of 0.1 or 1 for 16 h, then were treated with IFN-β (100 U/mL) for 15 min. Cells were lysed and analysed by Western blotting with antibodies against total/phosphorylated STAT1, STAT2, JAK1, Tyk2, FCV VP1 and β-actin. (B) CRFK cells were mock infected (Mock) or infected with FCV 2280 at an MOI of 0.1 or 1 for 16 h. Cells were lysed and were analysed by WB with antibodies against IFNAR1, IFNAR2, VP1 and β-actin. (C) CRFK cells were mock infected (Mock) or infected with FCV 2280 at an MOI of 1 for 16 h, then the cells were stained using PI to isolate live cells. Ethanol treated cells acted as a positive control. After that, the cells were fixed, then the surface expression of IFNAR1 and IFNAR2 as well as viral antigen were examined with rabbit anti-IFNAR1/2 antibody or mouse anti-FCV VP1 mAb by flow cytometry. The blots shown in A and B are representative of three independent experiments, and relative intensity levels were quantified using the ImageJ software. Differences (*p < 0.05, **p < 0.01, ***p < 0.001) between the experimental and control groups are noted.

IFN binding to its receptors induces a broad transcriptional response that is dependent on the phosphorylation of STAT1/2 induced by JAK1 and Tyk2 [43]. To investigate the mechanism by which FCV 2280 infection blocks the function of IFN, we first examined the effect of FCV 2280 infection on STAT1 and STAT2 phosphorylation. Both STAT1 and STAT2 are important adaptors of the JAK-STAT pathway [44], and many viruses can target one or both of these proteins to inhibit this pathway. CRFK cells were infected with FCV 2280 and were then treated with 100 U/mL IFN-β for 15 min. Total STAT1 and STAT2 proteins and both phosphorylated forms were detected by Western blotting. As shown in Fig 2A, FCV 2280 infection did not reduce the expression of total STAT1 and STAT2, but it significantly inhibited the levels of the phosphorylated STAT1 and STAT2 proteins in FCV-infected CRFK cells. Additionally, the inhibitory effect was in a virus dose-dependent manner. Next, we examined the phosphorylated levels of JAK1 and Tyk2, both of which are upstream adaptors of STAT1 and STAT2. The results showed that FCV 2280 infection contributed to the decrease of JAK1 and Tyk2 phosphorylation but did not reduce the expression levels of total JAK1 and Tyk2 (Fig 2A). Next, we explored whether FCV 2280 infection led to the decrease of interferon receptors IFNAR1 and IFNAR2, which inhibits downstream adaptor activation. As shown in Fig 2B, FCV 2280 infection reduced the level of IFNAR1 protein in a virus dose-dependent manner but did not affect the level of IFNAR2. IFNs bind to IFNAR1 on cytomembranes, and the interaction leads to the activation of downstream adaptors. To confirm that FCV 2280 infection reduced the level of cytomembrane IFNAR1, flow cytometry was used to detect the surface expression of IFNAR1 in the infected cells. The results showed the mean percentage of infected cells reached 87.2% upon 2280 infection at an MOI of 1 at 16 hpi (S1E Fig), and the surface expression of IFNAR1 but not IFNAR2 was significantly downregulated by nearly 24% upon FCV 2280 infection (Fig 2C). Secreted type I interferon functions by interacting with the IFNARs on the cell surface, but FCV 2280 infection blocks the activation of this signalling pathway by decreasing the expression level of IFNAR1.

### FCV 2280 infection contributes to the degradation of IFNAR1 mRNA

**Fig 3:**
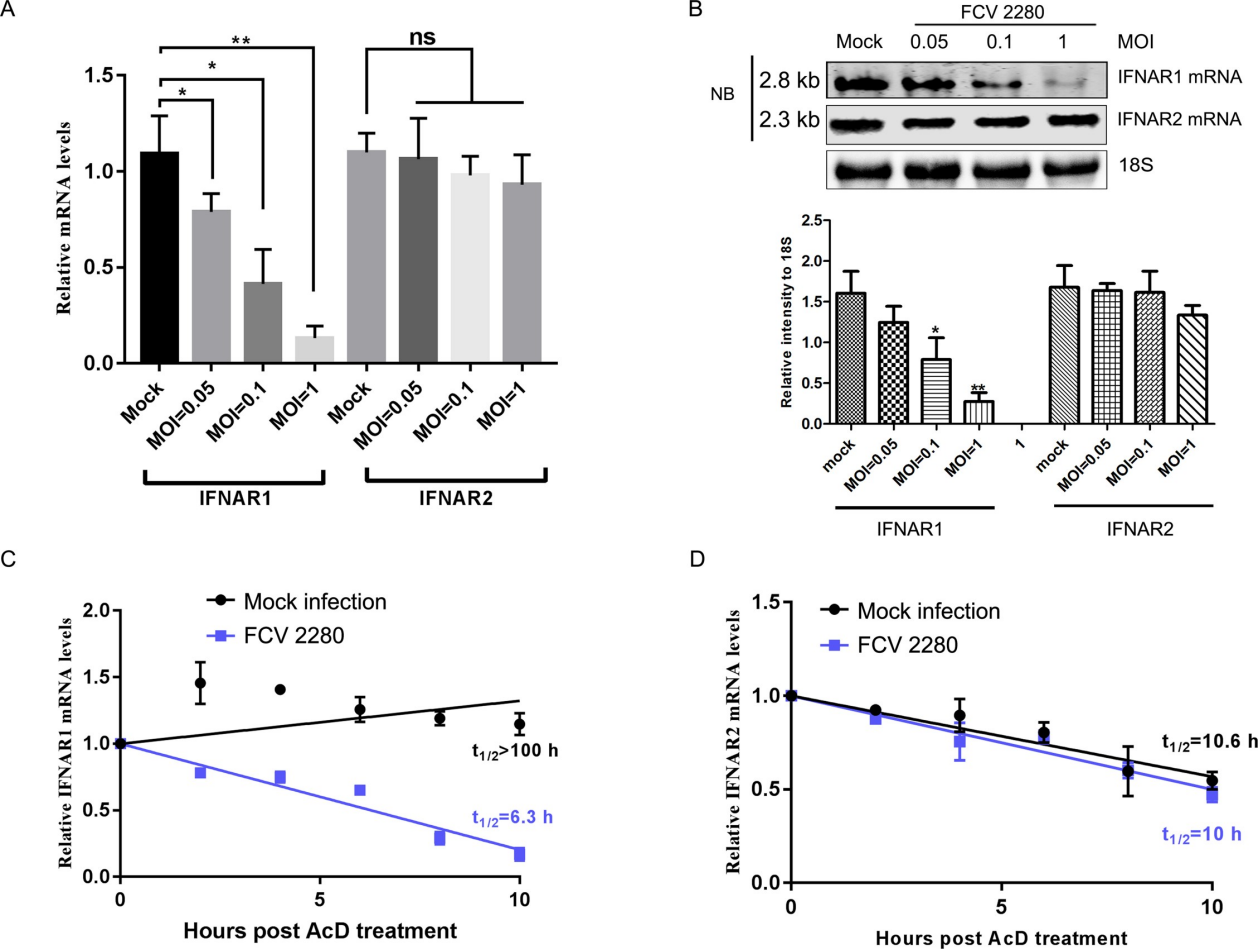
FCV 2280 infection contributes to IFNAR1 mRNA degradation. (A) CRFK cells were mock infected (Mock) or infected with FCV at an MOI of 0.05, 0.1 or 1 for 18 h. Total RNA was extracted. IFNAR1 and IFNAR2 mRNA levels relative to 18S rRNA were assessed by qRT-PCR. (B) CRFK cells were co-transfected with 1 μg of pcDNA3.1-IFNAR1 and -IFNAR2 for 12 h, and then mock infected (Mock) or infected with different doses of virus for 16 h. Total RNA was extracted for Northern blot (NB) assay. 18S RNA was used as a loading control. (C, D) CRFK cells were mock infected (Mock) or infected with FCV at an MOI of 1. At 30 min post-infection, intracellular RNA was extracted (0 h) or 5 μM Act.D was added to the culture. Total RNA was extracted at the indicated time points after Act.D addition. qRT-PCR was used to identify the levels of IFNAR1 (C) and IFNAR2 mRNA (D), and the mRNA half-life was calculated. The data in A, C, D represent the mean ± SD for three repeats, and all experiments A-D were repeated three times. Blots shown in B are representative of three independent experiments, and relative intensity levels were quantified using the ImageJ software. Differences (*p < 0.05, **p < 0.01, ***p < 0.001) between the experimental and control groups are noted.

To explore the mechanism by which FCV 2280 downregulates the expression of IFNAR1, we first analysed the level of IFNAR1 mRNA upon 2280 infection using qRT-PCR and Northern blotting (NB). CRFK cells were infected with FCV 2280, and total RNA was extracted. The IFNAR1 and IFNAR2 mRNA levels relative to 18S rRNA were assessed by qRT-PCR. As shown in Fig 3A, FCV 2280 infection significantly downregulated the expression of IFNAR1 mRNA in a virus dose-dependent manner, but it did not affect the level of IFNAR2 mRNA. To address any non-specificity of the qRT-PCR method, a Northern blot assay was conducted to further confirm the result. CRFK cells were co-transfected with 1 μg of pcDNA3.1-IFNAR1 and -IFNAR2 for 12 h and then infected with different doses of virus. Total RNA was extracted for NB assays. The NB results also confirmed that FCV 2280 infection led to the decrease of IFNAR1 mRNA in a virus dose-dependent manner (Fig 3B). Both results revealed that IFNAR1 mRNA expression was reduced during FCV 2280 infection.

Actinomycin D (Act.D) can block the DNA-dependent RNA polymerase activity [45] but cannot affect viral transcription and is used to evaluate whether viral protein reduces the level of host mRNA via degradation [37, 46]. To validate whether loss of IFNAR1 mRNA upon FCV 2280 infection can be attributed to mRNA degradation, the host gene transcription was first blocked by Act.D, and the cells were then inoculated with FCV 2280. qRT-PCR was used to determine the half-life of IFNAR1 and IFNAR2 mRNA. The half-life of IFNAR1 mRNA in FCV-infected cells was 6.3 hours (h), and that in mock cells was >100 h (Fig 3C). The levels of IFNAR1 mRNA were still reduced with Act.D treatment upon FCV 2280 infection, suggesting that the decreased IFNAR1 mRNA levels in FCV-infected cells were a result of enhanced mRNA degradation. The half-life of IFNAR2 mRNA in FCV-infected cells was 10 h, and that in mock cells was 10.6 h (Fig 3D). No significant difference was detected. These data demonstrated that FCV 2280 infection promotes the degradation of IFNAR1 mRNA.

### FCV 2280 p30 protein is attributed to the reduction of IFNAR1 mRNA

**Fig 4:**
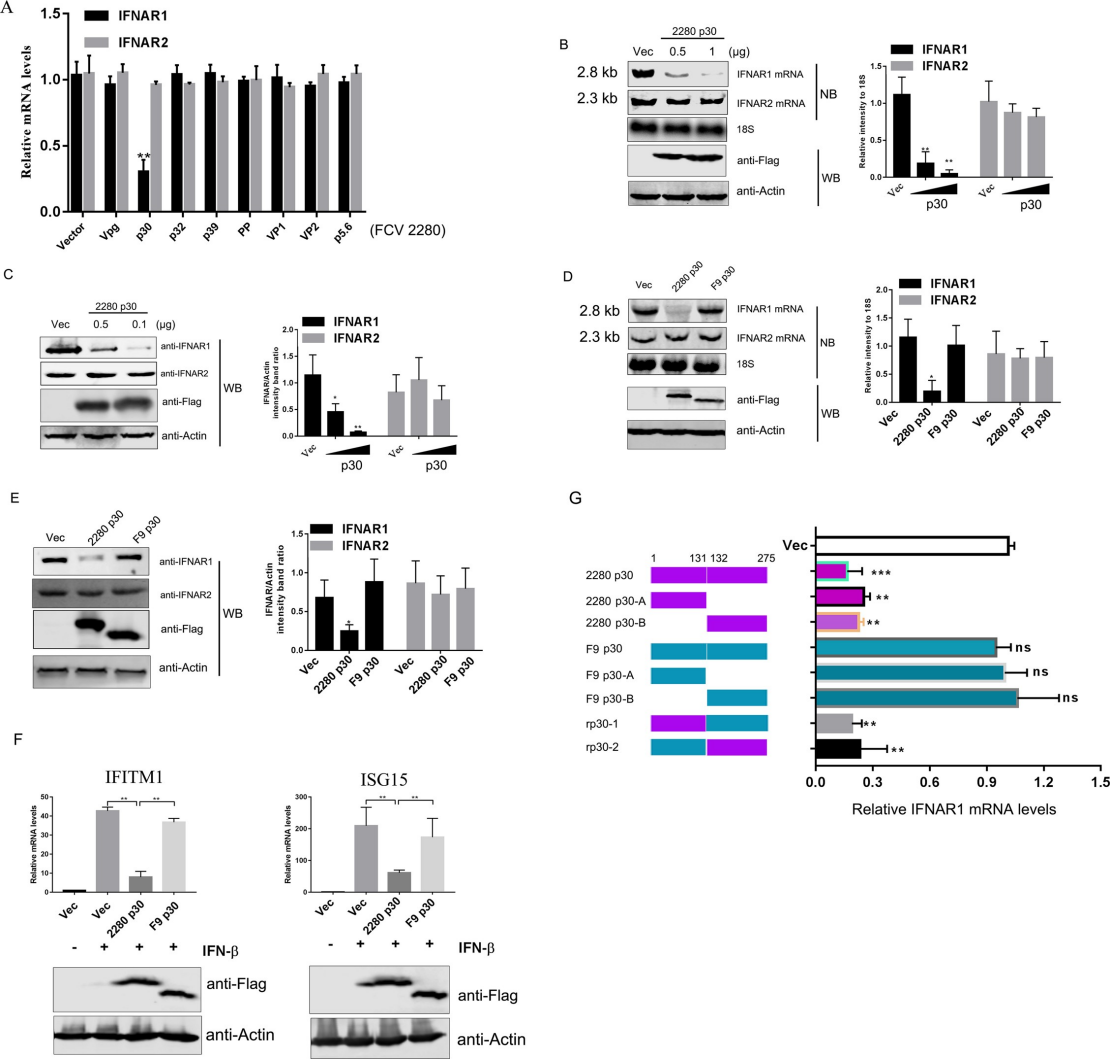
FCV 2280 p30 promotes IFNAR1 mRNA degradation. (A) Plasmids encoding FCV 2280 p5.6, p30, p32, p39, Vpg, PP, VP1, VP2 protein or empty vector (Vector) were transfected into CRFK cells for 24 h. The mRNA expression levels of IFNAR1/2 were determined by qRT-PCR. (B, C) Vector or different doses of plasmid encoding FCV 2280 p30, together with pcDNA3.1-IFNAR1 and pcDNA3.1-IFNAR2, were co-transfected into CRFK cells for 24 h. mRNA expression levels of IFNAR1/2 were then determined by NB assay; 18S rRNA was used as a loading control (B). The protein expression levels of IFNAR1/2 were evaluated by WB (C). (D, E) CRFK cells were co-transfected with plasmids encoding FCV strain 2280 and F9 p30 or Vec, as well as pcDNA3.1-IFNAR1 and pcDNA3.1-IFNAR2 for 24 h, and then the mRNA (D) and protein (E) expression levels of IFNAR1/2 were evaluated using NB and WB assay, respectively. (F) CRFK cells were transfected with plasmid encoding FCV strain 2280 and F9 p30 or Vec for 24 h, then the cells were incubated with IFN-β (1000 U/mL) for 10 h. Total RNA was extracted, and the mRNA expression levels of ISGs relative to 18S rRNA were measured by qRT-PCR. (G) CRFK cells were co-transfected with plasmids encoding FCV strain 2280 and F9 p30 as well as their truncated and chimeric mutants for 24 h, then the mRNA expression levels of IFNAR1 were determined by qRT-PCR. Blots shown in B to E are representative of three independent experiments, and relative intensity levels were quantified using the ImageJ software. The data in A, F, G represent the mean ± SD for three repeats, and all experiments A-G were repeated three times. Differences (*p < 0.05, **p < 0.01, ***p < 0.001) between the experimental and control groups are noted.

To explore which viral protein is responsible for the reduction of IFNAR1 mRNA upon FCV 2280 infection, CRFK cells were transfected with plasmids expressing each FCV 2280 protein (S2A Fig). IFNAR1 mRNA was then measured 24 hours post-transfection using qRT-PCR. As shown in Fig 4A, the presence of p5.6, p32, p39, Vpg, PP, VP1 and VP2 did not inhibit IFNAR1 mRNA expression; however, p30 expression led to a significant reduction. No proteins affected the expression of IFNAR2 mRNA. Moreover, a p30-induced reduction of IFNAR1 mRNA and protein as detected by NB (Fig 4B) and WB (Fig 4C), respectively displayed a dose-dependent relationship.

FCV strain F9 is a vaccine strain, and F9 *in vivo* replication is incompetent compared to 2280 (1). Moreover, F9 *in vitro* infection is sensitive to IFN treatment as shown in Fig 1A. We speculate that F9 p30 could not reduce the level of IFNAR1 mRNA. Next, we applied an NB assay to analyse the ability of F9 p30 to inhibit IFNAR1 mRNA expression. Compared with FCV 2280 p30, overexpression of F9 p30 did not affect IFNAR1 mRNA (Fig 4D) or protein (Fig 4E) expression. Moreover, we compared the effect of FCV 2280 and F9 p30 overexpression on ISG induction. The plasmids encoding FCV 2280 p30 and F9 p30 were separately transfected into CRFK cells. At 24 hours post-transfection, the cells were treated with IFN-β for 10 h, and the expression of ISG mRNA was then analysed by qRT-PCR. FCV 2280 p30 expression suppressed the IFITM1 and ISG15 mRNA expression induced by IFN-β, but F9 p30 failed to do so (Fig 4F). These data demonstrate that FCV strain 2280 p30 can promote the reduction of IFNAR1 mRNA.

To examine the key domain of 2280 p30, a series of deleted constructs were prepared (Fig 4G Left) and expressed (S2B Fig), then were evaluated regarding their effect on IFNAR1 mRNA expression using qRT-PCR. As shown in Fig 4G, both the N terminal (aa 1–131) and the C terminal (aa 132–275) of 2280 p30 contain a domain that induces the reduction of IFNAR1 mRNA expression. The two fragments were cloned into the corresponding domains of F9 p30, and both chimeric F9 p30 proteins promoted a reduction in IFNAR1 mRNA (Fig 4F), confirming that both the N and C terminals of 2280 p30 are the key domain for its ability.

### His-2280 p30 fusion protein directly degrades IFNAR1 RNA

**Fig 5:**
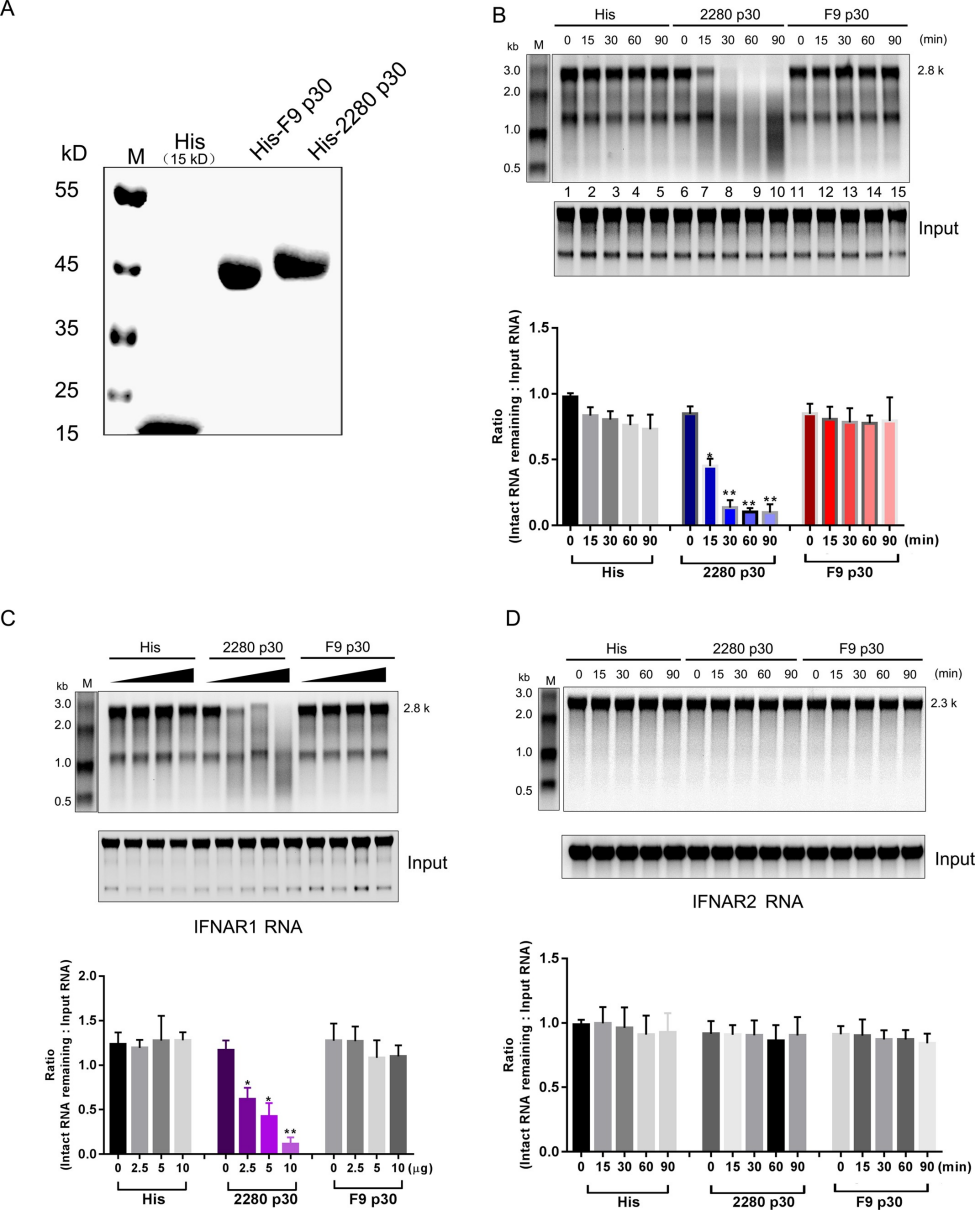
*In vitro* assay of p30-mediated degradation of IFNAR1 RNA. (A) Proteins are from *E*. *coli* expressing His-2280 p30 fusion protein, His-F9 p30 fusion protein, or His tag. A Coomassie blue-stained gel of recombinant proteins is shown. (B, D) His (10 μg), His-2280 p30 fusion protein or His-F9 p30 fusion protein (10 μg) was incubated at 30°C with 4 μg of IFNAR1 (B) or IFNAR2 RNA (D) transcribed *in vitro*. At the indicated times, the RNA was purified and resolved on an agarose-formaldehyde gel. (C) His (10 μg) or His-2280 p30 fusion protein or His-F9 p30 fusion protein (0–10 μg) were incubated at 30°C with IFNAR1 RNA (4 μg) transcribed *in vitro*. After 90 min of incubation, the RNA was purified and resolved on an agarose-formaldehyde gel. The gel electrophoresis results shown in B, C and D are representative of three independent experiments, and the relative intensity of intact RNA remaining was quantified using the ImageJ software. Differences (*p < 0.05, **p < 0.01, and ***p < 0.001) between the experimental and control groups are noted.

To explore whether 2280 p30 protein directly degrades IFNAR1 RNA but dose not target IFNAR2 RNA, the His-p30 fusion proteins of 2280 and F9 were purified from *E*. *coli* (Fig 5A), and IFNAR1 and IFNAR2 RNA containing the 5’ and 3’UTRs were used as the substrate. Equal amounts of His-p30 fusion protein or His alone were incubated at 30°C with the RNA for the indicated time points; samples were then withdrawn and analysed by agarose-formaldehyde gel electrophoresis. As shown in Fig 5B, IFNAR1 RNA mixed with His (Lanes 1–5) or His-F9 p30 fusion protein (Lanes 11–15) remained stable throughout the 90 min incubation time, but IFNAR1 RNA decayed in the presence of His-2280 p30 fusion protein (Lanes 6–10). Moreover, His-2280 p30 fusion protein induced degradation of IFNAR1 RNA in a dose-dependent manner (Fig 5C). Consistent with the cell-based results, an *in vitro* system demonstrated that the p30 proteins of F9 and 2280 did not induce degradation of IFNAR2 RNA containing a 5’ and 3’UTR (Fig 5D).

These data reveal that FCV 2280 p30 protein displays an RNase-like activity and promotes the degradation of IFNAR1 mRNA.

### The 5’ UTR of IFNAR1 mRNA induces selective cleavage by 2280 p30

**Fig 6:**
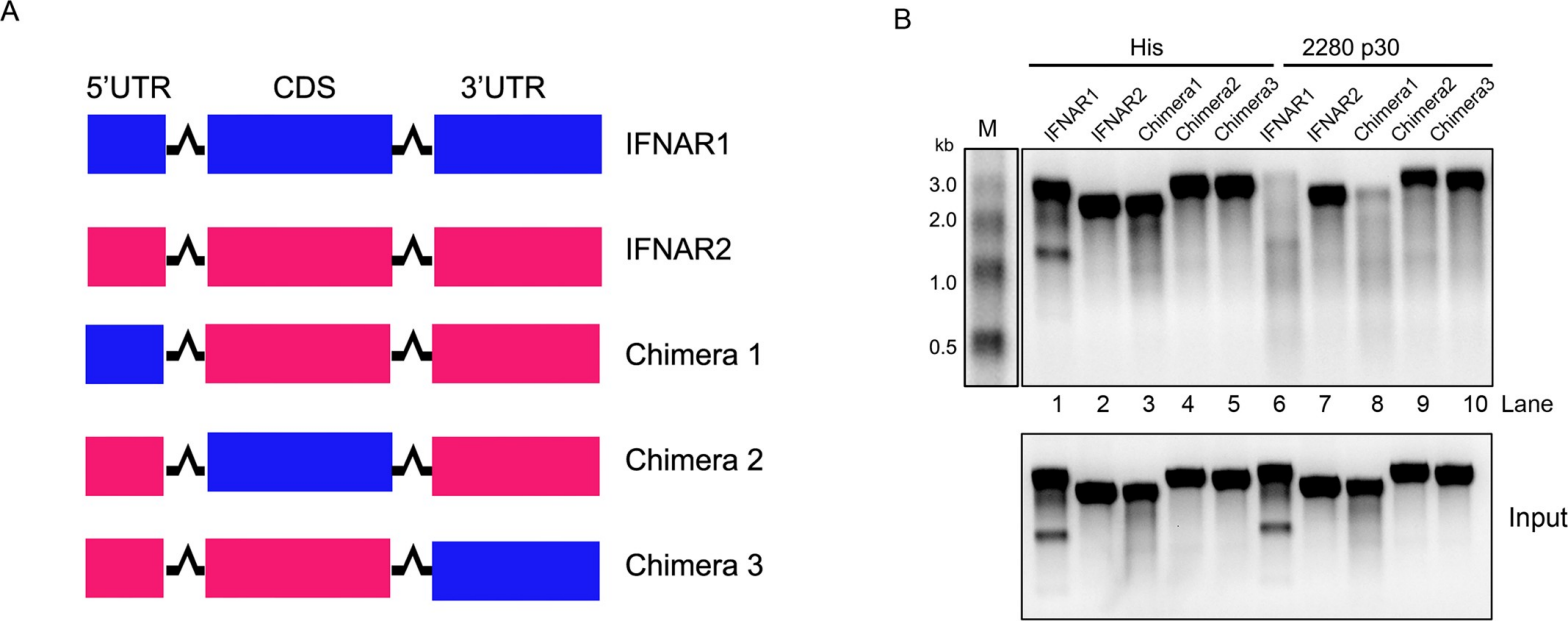
The 5’UTR of IFNAR1 confers selective degradation. (A) Schematic ofchimeric IFNAR2 fused with the 5’UTR, the coding sequence (CDS), or the 3’UTR of IFNAR1. (B) His (10 μg) or His-2280 p30 fusion protein (10 μg) were incubated at 30°C with 4 μg of IFNAR1 or IFNAR2 RNA or chimeric IFNAR2 transcribed *in vitro*. After 60 min of incubation, the RNA was purified and resolved on an agarose-formaldehyde gel. The gel electrophoresis result is representative of three independent experiments.

The ability of 2280 p30 to selectively target IFNAR1 mRNA may be due to the presence of a special element or structure that does not exist in IFNAR2 mRNA. To confirm this hypothesis, we fused the 5’ untranslated region (UTR), the coding sequences (CDS), or 3’ UTR of IFNAR1 mRNA to IFNAR2 mRNA (Fig 6A). The chimeric RNA was produced using a T7 *in vitro* synthesis kit, and the abundance of each RNA in the presence or absence of 2280 p30 was monitored using an *in vitro* degradation system. IFNAR1 RNA degradation was markedly increased in the presence of 2280 p30 (Fig 6B Lane 6), but IFNAR2 RNA was not affected (Fig 6B Lane 7). When the IFNAR2 5’UTR was substituted with IFNAR1 5’UTR, the chimeric RNA was readily degraded by 2280 p30 (Fig 6B Lane 8), but another two chimeric RNAs were not degraded (Fig 6B Lanes 9, 10). These results suggest that the 5’ UTR of IFNAR1 plays a role in selectively targeting mRNA for degradation by 2280 p30.

### 2280 p30 displays the shutoff phenotype

**Fig 7:**
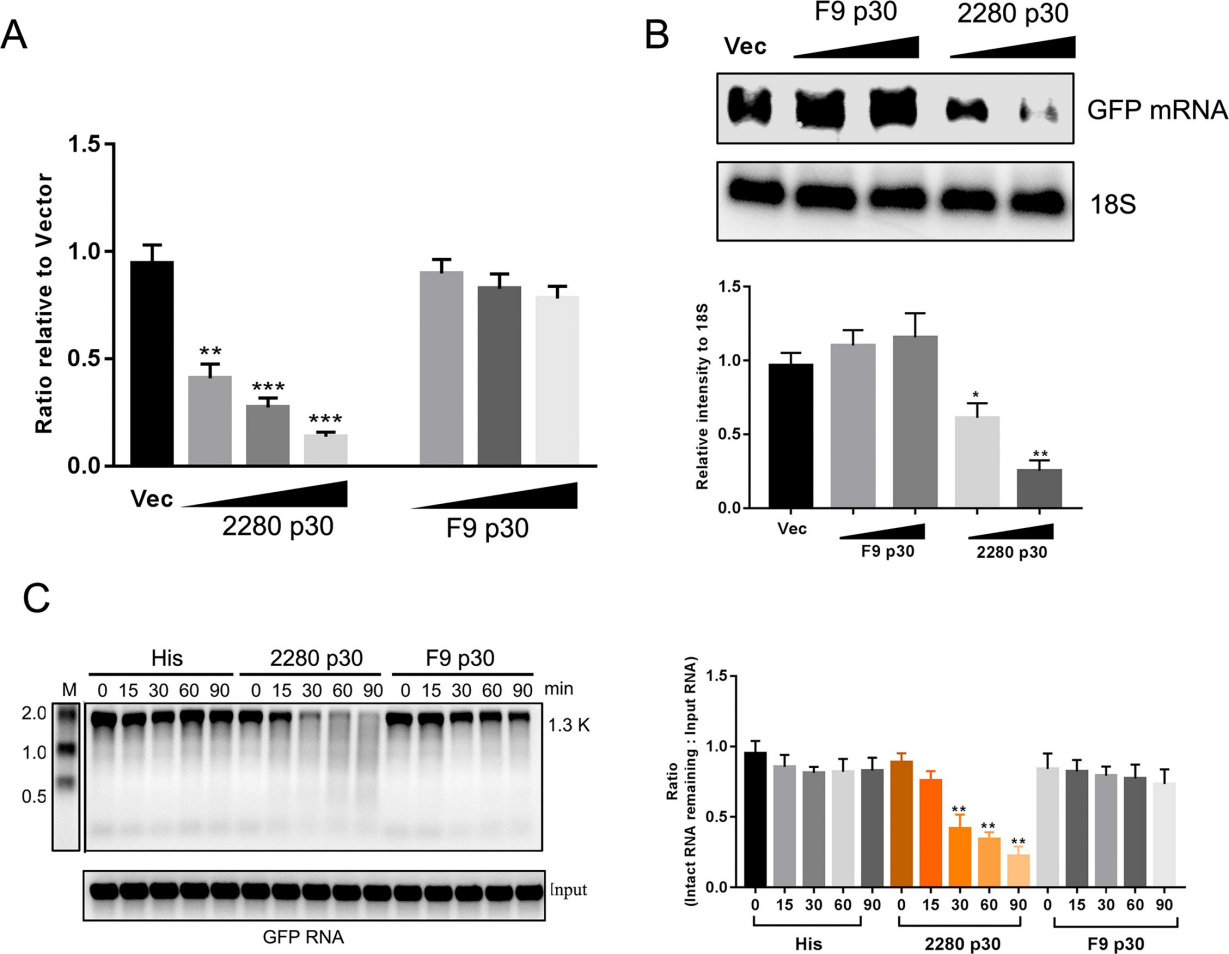
2280 p30 is a virion shutoff protein. (A) CRFK cells were cotransfected with the plasmid encoding 2280 p30 or F9 p30 and a reporter plasmid, pRL-TK. The luciferase activity was evaluated, and relative activity was normalized to the empty vector group. (B) CRFK cells were cotransfected with plasmid encoding 2280 p30 or F9 p30 and pEGFP-N1. At 24 h, cellular total RNA was extracted for NB assay. (C) His (10 μg) or His-2280 p30 fusion protein or His-F9 p30 fusion protein (10 μg) were incubated at 30°C with GFP RNA (4 μg) transcribed *in vitro*. At the indicated times, the RNA was purified and resolved on an agarose-formaldehyde gel. The gel electrophoresis result is representative of three independent experiments, and the relative intensity of intact RNA remaining was quantified using the ImageJ software. Differences (*p < 0.05, **p < 0.01, and ***p < 0.001) between the experimental and control groups are noted.

Because 2280 p30 selectively degraded host IFNAR1 mRNA in a direct manner, we suspected that p30 may be a virion shutoff protein that can selectively degrade host mRNA. These shutoff proteins can degrade mRNA in the absence of other viral proteins, as confirmed by the inhibition of reporter gene expression in cells transiently cotransfected with the virion shutoff gene [47]. To demonstrate whether 2280 p30 is a virion shutoff protein, CRFK cells were cotransfected with the reporter plasmid pRL-TK and the plasmid encoding 2280 p30 or F9 p30. 2280 p30 expression significantly inhibited luciferase activity, but F9 p30 expression did not (Fig 7A). Further, we evaluated the effect of p30 expression on another reporter gene, GFP mRNA. CRFK cells were cotransfected with pEGFP-N1 and the plasmid encoding either 2280 p30 or F9 p30. After 24 h, total cellular RNA was extracted for NB assay. As shown in Fig 7B, 2280 p30 expression significantly reduced the level of GFP mRNA, but F9 p30 expression did not. An *in vitro* degradation assay also showed that the 2280 p30 fusion protein could decay GFP RNA but that F9 p30 fusion protein could not (Fig 7C). These results revealed that 2280 p30 is a virion shutoff protein.

### p30 protein mediates interferon resistance in FCV 2280 infection

**Fig 8:**
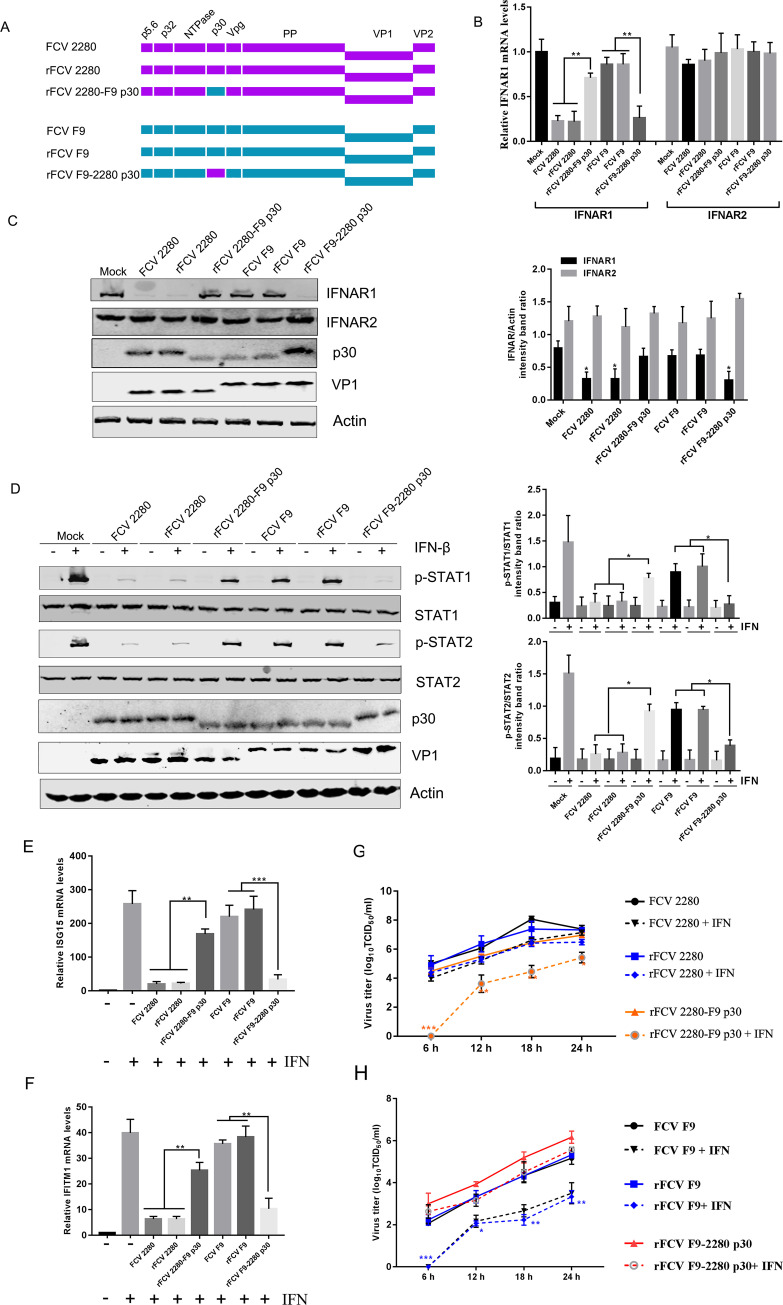
p30 confers resistance to the IFN-mediated antiviral response. (A) Diagram for construction of chimeric viruses. (B, C) CRFK cells were inoculated with FCV 2280, rFCV 2280 and rFCV 2280 F9-p30 or FCV F9, rFCV F9 and rFCV F9-2280-p30 at an MOI of 1, and the mRNA and protein levels of IFNAR1 were assayed using qRT-PCR (B) and WB (C) at 16 h post-infection. (D) CRFK cells were inoculated with FCV 2280, rFCV 2280 and rFCV 2280 F9-p30 or FCV F9, rFCV F9 and rFCV F9-2280-p30 at an MOI of 1, and at 16 h post-infection, the cells were treated with or without porcine IFN-β (100 U/mL) for 20 minutes. The cells were subjected to WB analysis. (E, F) CRFK cells were infected with FCV 2280, rFCV 2280 and rFCV 2280 F9-p30 or FCV F9, rFCV F9 and rFCV F9-2280-p30 at a MOI of 0.1 and 1 for 8 h, then were treated with IFN-β (1000 U/mL) for 10 h. The mRNA levels of ISG15 (E) and IFITM1 (F) relative to 18S rRNA were determined by qRT-PCR. (G, H) CRFK cells were pretreated with IFN-β (1000 U/mL) or medium only. After 8 h, the cells were infected with FCV 2280, rFCV 2280 and rFCV 2280 F9-p30 (G) or FCV F9, rFCV F9 and rFCV F9-2280-p30 (H) at an MOI of 1 and then harvested at the indicated time points. Viral titres were determined by TCID_50_. The blots shown in C, D are representative of three independent experiments, and relative intensity levels were quantified using the ImageJ software. The data in E, F, G, H shown represent the mean ± SD for three repeats, and all experiments were repeated three times. Differences (*p < 0.05, **p < 0.01, ***p < 0.001) between the experimental and control groups are noted.

To further investigate the role of p30 in blocking the activation of the interferon downstream signalling pathway, the gene sequence encoding FCV strain 2280 p30 was substituted with the gene sequence encoding F9 p30, generating recombinant rFCV 2280-F9 p30. Additionally, the gene sequence encoding F9 p30 was substituted with the gene sequence encoding 2280 p30, generating recombinant rFCV F9-2280 p30 (Fig 8A). Moreover, wild-type 2280 and F9 were also rescued using reverse genetic system, generating recombinant rFCV 2280 and rFCV F9 (Fig 8A).

We first analysed the effect of wild-type and recombinant FCV infection on IFNAR1 mRNA and protein expression. CRFK cells were inoculated with wild type 2280, rFCV 2280 and rFCV 2280-F9 p30 or wild type F9, rFCV F9 and rFCV F9-2280-p30, and the IFNAR1 mRNA and protein levels were assayed using qPCR and WB. As shown in Fig 8B, wild-type and rFCV 2280 infection contributed to a 70–80% reduction in IFNAR1 mRNA expression, but rFCV 2280 F9-p30 infection only contributed to a 20–30% reduction; wild-type and rFCV F9 infection contributed to a 10–15% reduction of IFNAR1 mRNA expression, but rFCV F9-2280 p30 infection contributed to a 70–80% reduction. Analysis of IFNAR1 protein levels upon infection also demonstrated that IFNAR1 expression in the rFCV 2280 F9-p30 infection group was higher than that in the wild type and rFCV 2280 infection groups (Fig 8C); the IFNAR1 protein level in the rFCV F9-2280 p30 infection group was lower than that in wild type and rFCV F9 infection groups (Fig 8C).

Next, to evaluate the role of p30 in inhibiting type I IFN signalling during FCV 2280 and F9 infection, CRFK cells were inoculated with wild type 2280, rFCV 2280 and rFCV 2280 F9-p30 or wild type F9, rFCV F9 and rFCV F9-2280-p30, and at 16 hpi, the cells were treated with or without IFN-β. The cells were subjected to Western blot analysis for p-STAT1, STAT1, p-STAT2 and STAT2, as well as viral protein p30 and VP1 for the viral loading control. Compared with the wild type and rFCV 2280 infection groups, infection with rFCV 2280 F9-p30 led to less of a reduction in the IFN-induced STAT1 and STAT2 phosphorylation (Fig 8D). Compared with the wild type and rFCV F9 infection groups, rFCV F9-2280 p30 infection contributed to a greater reduction in the IFN-induced STAT1 and STAT2 phosphorylation (Fig 8D). qRT-PCR analysis for ISG expression also showed that the expression of IFITM1 and ISG15 in the rFCV 2280 F9-p30 infection group was higher than that in the wild-type and rFCV 2280 infection groups, and the expression of both in the rFCV F9-2280 p30 group was lower than those in the wild-type and rFCV F9 infection groups (Fig 8E and 8F).

To further examine whether p30 antagonizes the anti-viral function of IFN during FCV infection, we pretreated CRFK cells with IFN-β and then infected the cells with the indicated viruses (Fig 8G and 8H). Compared with the replication of wild-type and rFCV 2280, rFCV 2280-F9 30 displayed lower growth kinetics. IFN-β treatment contributed to slightly impaired replication of wild-type and rFCV 2280 but led to an at least 100-fold reduction in the replication of rFCV 2280-F9 p30 (Fig 8G). When comparing the wild-type, rFCV F9 and rFCV F9-2280 p30 groups, the replication of rFCV F9-2280 p30 was faster than the replication of wild-type and rFCV F9 (Fig 8H). IFN-β treatment led to at least a 100-fold reduction in wild-type and rFCV F9 replication, but it only slightly impaired the replication of rFCV F9-2280 p30 (Fig 8H).

These data indicate that FCV 2280 p30 mediates evasion of the IFN-induced antiviral activity and promotes viral replication. Taken together, these results indicate that p30 indeed plays a role in antagonizing type I IFN signalling during FCV 2280 infection.

### FCV p30 affects clinical symptoms, replication and shedding in cats

Reports have indicated that viral proteins with anti-IFN activity can affect viral virulence. We examined the effect of p30 on the virulence of FCV. The clinical symptoms caused by the virus, including replication in the lung and trachea and shedding in the eye, nasal passage and throat, were evaluated after infection in cats.

**Fig 9:**
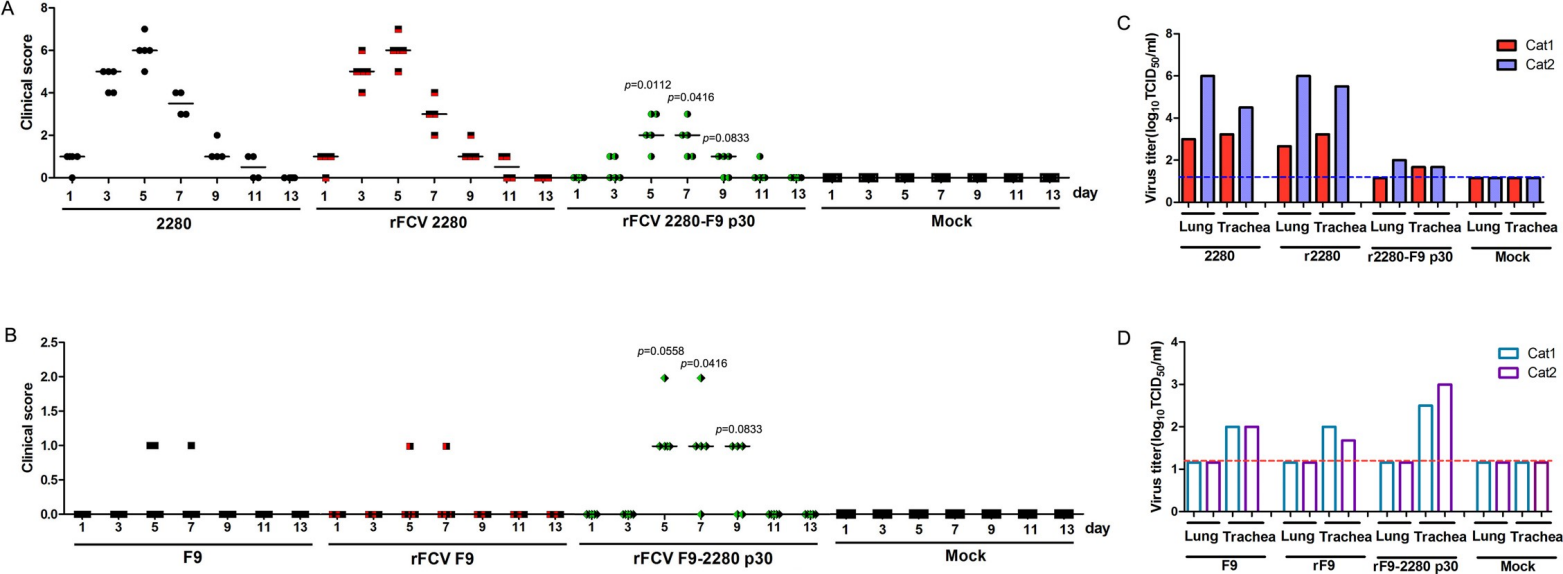
Examination of clinical symptoms and virus titres in tissues during infection. (A, B) After infection, the clinical signs were monitored daily and scored according to Table 2. Each score for each animal and the median were shown. Differences (Mann-Whitney U test, p < 0.05 as a significant difference) between the experimental and control groups are noted. (C, D) On day 5 post-infection, two cats from each group were euthanized and lung and trachea were harvested to assess the virus titre. Each lung lobe (1 g) was collected and mixed, then homogenized together. The horizontal dashed lines indicate the lower limit of detection.

The clinical score of wild-type and rFCV 2280 infection groups were significantly higher than that of the rFCV 2280-F9 p30 group from day 3 to day 7, suggesting that F9 p30 substitution in the backbone of 2280 attenuated the clinical manifestations (Fig 9A). In contrast, 2280 p30 substitution in the backbone of F9 enhanced the clinical manifestations from day 5 to day 9 (Fig 9B). These results indicated that p30 affects the severity of FCV clinical symptoms.

Viral shedding in eye swabs from the wild-type and rFCV 2280 infection groups could be detected from day 1 to day 9 but was not detected in the rFCV 2280-F9 p30 infection group (S3A Fig). Moreover, F9 p30 substitution in the backbone of 2280 decreased viral shedding in the nasal passage and throat from day 3 to day 11 (S3B Fig). For F9 and its recombinant viruses, no viral shedding was detected in the eyes, but 2280 p30 substitution in the backbone of F9 enhanced viral shedding in the nasal passage from day 7 to day 11 (S3B Fig) and throat from day 3 to day 7 (S3C Fig). These findings demonstrate that p30 affects FCV shedding.

Viral replication in the lung and trachea was evaluated on day 5. As shown in Fig 9C, the viral load in both tissues of the rFCV 2280-F9 p30-infected cats was lower than that of the wild-type- and rFCV 2280-infected cats, suggesting that F9 p30 substitution in the backbone of 2280 attenuated viral replication in the lung and trachea. In contrast, 2280 p30 substitution in the backbone of F9 enhanced viral replication only in the trachea (Fig 9D). These findings demonstrate that p30 affects FCV replication. Histology analysis of the lung in the mock- and virus-infected cats was performed (S4 Fig). Pathology of the rFCV 2280-F9 p30-infected cats lung was lower than that of the wild-type- and rFCV 2280-infected cats. While viral detection was negative in the lungs of F9- and its mutants-infected cats, their infection also caused a few lung epithelia cells hyperplasia, but 2280 p30 substitution in the backbone of F9 enhanced the lung epithelia cells hyperplasia.

Taken together, these findings show that FCV p30 is a virulence factor.

## Discussion

The innate immune system plays an essential role in the host first-line defence against microbial invasion. Once the host is infected by a pathogen, host pattern recognition receptor (PRR) can readily recognize the pathogen-associated molecular patterns (PAMPs), which activate the innate immune system and ultimately release a series of cytokines such as interferons and inflammatory factors. Type I interferon can activate the transcription of interferon-stimulated genes (ISGs) through the JAK-STAT pathway, and these ISGs exert an efficient antiviral effect [16, 48]. To survive, some viruses have also evolved many strategies to inhibit the host antiviral response. Yumiketa *et al*. reported that nonstructural protein p39 of feline calicivirus suppresses the host innate immune response by preventing IRF-3 activation [49]. Another group found a novel open reading frame (ORF4) encoded by murine norovirus (MNV) subgenomic RNA, which antagonizes the innate immune response to infection by delaying the upregulation of a number of cellular genes activated by the innate pathway, including IFN-β [50]. In the current study, we found that a virulent FCV strain 2280 [1] could inhibit the host type I IFN-mediated antiviral signalling pathway. 2280 infection blocked the activation of JAKs and STATs by degrading IFNAR1 mRNA. Screening of the FCV proteins revealed that nonstructural protein p30 contributed to the reduction of IFNAR1 mRNA. A comparative test between p30 from FCV vaccine strain F9 and strain 2280 showed that F9 p30 failed to degrade IFNAR1 mRNA. Moreover, *in vitro* degradation assays demonstrated that 2280 p30 could directly degrade IFNAR1 RNA but that F9 p30 could not. Importantly, we constructed recombinant viruses expressing chimeric p30 using a reverse genetic system and found that p30 led to interferon resistance and could affect FCV virulence. Our findings demonstrated that p30 helps FCVs to evade the host antiviral response.

The JAK-STAT signalling pathway is responsible for transmitting extracellular chemical signals to the nucleus and plays an important role in Type I IFN signal transmission. Thus, it is logical that the JAK-STAT pathway is a target for viruses to subvert host antiviral immunity [48]. West Nile Virus can downregulate the host IFNAR1 protein level [51, 52]. Porcine epidemic diarrhoea virus inhibits interferon signalling via the targeted degradation of STAT1 [27]. Porcine reproductive and respiratory syndrome virus inhibits type I interferon signalling by blocking STAT1/STAT2 nuclear translocation [53]. Porcine Deltacoronavirus nsp5 antagonizes type I interferon signalling by cleaving STAT2 [26]. In this study, we found that FCV 2280 infection blocks the IFN-β-induced activation of the JAK-STAT pathway by directly and selectively degrading IFNAR1 mRNA.

FCV has developed strategies to inhibit host protein synthesis and promote viral protein synthesis [54]. FCV infection inhibits host protein synthesis by cleaving eukaryotic initiation factor eIF4G and poly (A)-binding protein (PABP) [55, 56]. The reported inhibitory effect occurred at translation steps of eukaryotic protein expression [55, 56], but we found that FCV infection inhibited IFNAR1 expression through the direct degradation of IFNAR1 mRNA by p30. Our previous work demonstrated that FCV strain 2280 proteinase-polymerase (Pro-Pol) protein can suppress luciferase reporter gene expression driven by endogenous and exogenous promoters, thus inhibiting the host gene expression [2]. However, the overexpression of Pro-Pol did not inhibit the expression of IFNAR1 mRNA.

Infection with gammaherpesviruses, alphaherpesviruses, or betacoronaviruses can lead to widespread and selective mRNA degradation by a single viral factor [34]. Subsets of cellular proteins are resistant to host shutoff and may facilitate viral replication [57]. However, no precise mechanism has been found to explain the action of selective mRNA degradation. This selectivity may be due to particular elements within mRNA. During lytic gammaherpesvirus infection, viral SOX protein induces widespread mRNA degradation, but some mRNA, such as IL-6, can escape this fate [58]. The IL-6 mRNA contains an ~100-nucleotide element within its 3’UTR that renders it refractory to decay by SOX [58]. In this study, we found that the 5’UTR of IFNAR1 mRNA renders it susceptible to cleavage by 2280 p30 because substitution of the 5’UTR of IFNAR2 mRNA by that of IFNAR1 mRNA conferred susceptibility to 2280 p30-induced mRNA degradation. Variable nucleotide sequences between the 5’UTR of IFNAR1 and IFNAR2 (S5A Fig) produces two different RNA secondary structures (S5A Fig). The variability in the sequences and RN secondary structures may lead to 2280 p30 binding IFNAR1 mRNA, then cleaving it.

A recent study demonstrated that MHV68-induced mRNA decay during lytic infection also leads to a genome-wide reduction of Pol II occupancy at mammalian promoters, which accelerates host mRNA decay [59]. Whether FCV-induced mRNA decay also contains a similar mechanism need to be further investigated. MHV68 gene expression is resistant to the effects of mRNA degradation, and viral genes are robustly transcribed during the stage of infection when host transcription is reduced [59]. Sequences located on the viral genome are both necessary and sufficient to escape the transcriptional repression effects of mRNA decay [59]. We also found that FCV-induced mRNA decay does not affect the abundance of viral RNA. The host RNA abundance decreases, but instead viral RNA abundance increases, which may be attributed to viral sequence or host factors.

The HSV-1 vhs is an endoribonuclease and selectively degrades host mRNA [34]. It shows a strong preference for constitutively expressed mRNAs and some inducible mRNAs [47]. It blocks the host innate antiviral response by reducing the level of ISG mRNA [60, 61]. In this study, RNA-seq revealed that more than two thousand genes are downregulated upon FCV infection. Among these, IFNAR1 mRNA was selectively downregulated, which may block the upregulation expression of ISGs. Moerover, p30 is a viral shutoff protein which could also inhibit some inducible mRNA, so p30 may also inhibit the upregulation expression of ISGs that belong to inducible expression genes.

Purified GST-vsh fusion protein exhibits RNase activity [47]. FCV 2280 p30 fusion protein also displayed RNase-like activity and directly cleaved IFNAR1 RNA. Interestingly, the F9 p30 fusion protein displayed no degradation activity. Strain F9 is a less virulent virus, but the substitution of 2280 p30 in the backbone of F9 increased the virulence of the chimeric virus. FCV strains generally present low virulence in cats; however, a number of FCV strains with different levels of virulence have been isolated in recent years [4, 62, 63]. Our previous study demonstrated that FCV strain 2280 can infect the lung and trachea of cats, and the mortality rate reached to 20~40% [1]. FCV strain 2280 p30 evades IFN-induced antiviral activity and promotes viral replication, which may be a cause of the difference in virulence between F9 and 2280. Comparison among the amino acid sequences of different strains p30 revealed that the identity ranges from 86.1 to 95.6%. The sequence identity between strain 2280 and F9 p30 is 90.2% and a total of 25 amino acids are different. Since both the N and C terminals of 2280 p30 are the key domain for its ability, we could not identify the key amino acids for its shutoff activity. So, the sequence characteristics could not differentiate which FCV strains exhibit the shutoff activity or are resistant to IFN treatment.

In conclusion, we have shown that FCV 2280 infection blocks the JAK-STAT pathway by promoting the degradation of IFNAR1 mRNA. We have also provided evidence that FCV nonstructural protein p30 is able to directly degrade IFNAR1 RNA and is a key virulence factor. Our findings reveal a new mechanism by which some strains of FCV subvert host antiviral immunity.

## Materials and methods

### Cells, viruses and reagents

Crandell Rees feline kidney (CRFK) cells and F81 cells were maintained in Dulbecco’s modified minimum essential medium (DMEM) (Gibco) containing 10% foetal bovine serum (Gibco), 100 U/mL penicillin, and 100 μg/ml streptomycin at 37°C under an atmosphere containing 5% CO_2_. FCV strain 2280 and F9 were acquired from ATCC and propagated in CRFK cells. Vesicular stomatitis virus expressing green fluorescent protein (VSV-EGFP) was grown and titered in 293T cells and stored at -80°C. The mouse anti-p30 polyclonal antibodies were prepared by our lab. Universal Type I interferon beta (11415) was purchased from PBL Assay Science. Actinomycin D (HY-17559) was purchased from MedChem Express (MCE, Shanghai, China).

### Plasmid construction

The p3xFlag-p30, p3xFlag-p32, p3xFlag-p39, p3xFlag-Vpg, p3xFlag-PP, p3xFlag-VP1, and p3xFlag-VP2 plasmids were described previously [42]. The pRL-TK plasmid expressing the Renilla luciferase protein was purchased from Promega; pISRE-TA-Luc plasmid was purchased from Clontech. The 5’ and 3’UTRs of IFNAR1 and IFNAR2 were obtained using 5’ and 3’RACE kits (Clontech), and the full-length IFNAR1 and IFNAR2 genes were cloned into NheI/KpnI sites of pCDNA3.1(+). The chimeric IFNAR2s were cloned into NheI/KpnI sites of pCDNA3.1(+) using overlap PCR. The T7 promoter-derived GFP gene was cloned into pJet1.2 vector (ThermoFisher).

### Comparative transcriptome analysis upon FCV 2280 infection

CRFK cells were mock infected or infected with FCV 2280 at an MOI of 0.1 for 16 h, then performed RNA extraction, cDNA library construction, Illumina deep sequencing and data analysis [64, 65], which was completed by the Shanghai Majorbio Bio-pharm Biotechnology Co. (Shanghai, China). Each group contained two samples. All the raw read counts have been normalized by the principle of “reads per kilo bases per million reads” and ratio between average read counts from FCV infection group and mock group was shown. All the downregulated genes upon infection were shown in S1 Table. RNA seq data analysis is based on DESeq2 software (p-adjust < 0.05 && |log_2_Fold Change| ≥ 1) and EdgeR software is also used for differentially expressed analysis (p-adjust < 0.05 && | log_2_Fold Change | ≥ 1).

### Luciferase assay

The protocol used for the luciferase assay has been previously described [42]. Briefly, CRFK cells (2×10^5^/well) grown in 48-well plates were cotransfected with 200 ng/well reporter plasmid pISRE-TA-Luc and 20 ng/well pRL-TK plasmid (Promega) (as an internal control for normalization of the transfection efficiency). Luciferase activities were determined with the Dual-Luciferase Reporter Assay System (Promega) according to the manufacturer’s protocol. The relative luciferase activity in each sample was determined using the ratio between the activities of firefly and Renilla luciferases. The data are expressed as the mean±standard error, and at least 3 separate experiments were performed in triplicate.

### Virus titration

The TCID_50_ assay for virus titration has been previously described [42]. The viral titres are expressed as the median tissue culture infective dose Log_10_(TCID_50_/ml) according to the method of Reed and Müench [66].

### Quantitative real-time PCR (qRT-PCR)

Total RNA was prepared with an RNeasy Mini Kit (Qiagen, Valencia, CA, USA), and cDNA was obtained using the FastKing-RT SuperMix containing DNase (TIANGEN, China) according to the manufacturer’s protocol. qRT-PCR was performed by a qTOWER 2.0 (Analytik Jena AG, Jena, Germany). The relative mRNA expression levels were calculated by the 2^-ΔΔCT^ method using 18S rRNA as an internal control for normalization. The specific primers used are listed in Table 1.

**Table 1:** Primers for qRT-PCR and production of probes.

Primer name	Primer sequence (5’-3’)	Use
fe-IFN-β-F	GAAGGAGGAAGCCATATTGGT	qRT-PCR
fe-IFN-β-R	CTCCATGATTTCCTCCAGGAT	
q18S rRNA-F	CGGCTACCACATCCAAGGAA	
q18S rRNA-R	GCTGGAATTACCGCGGCT	
qIFNAR1-F	TTGCCTGGGTGTCAATCT	
qIFNAR1-R	GCCTTATCTTCGGCTTCT	
qIFNAR2-F	TGTCTTTGGAACCACCCG	
qIFNAR2-R	ATCTTCCCTGACTGTTCTTCG	
qIFITM1 F	CACCACCGTGATCAACATCCA	
qIFITM1 R	GACTTCACGGAGTAGGCAAAG	
qViperin F	CATGACCGGGGCGAGTACCTG	
qViperin R	GCAAGGATGTCCAAATATTCACC	
qISG15 F	TCCTGGTGAGGAACCACAAGGG	
qISG15 R	TTCAGCCAGAACAGGTCGTC	
Probe-IFNAR1-F	AAATCTAAAATCTCCTGAAAA	Production of probes
Probe-IFNAR1-R	AAACAGTAAGTCGTCTCTGGT	
Probe-IFNAR2-F	AAGATGCTTTGGAGCCAGAAT	
Probe-IFNAR2-R	AATGACTGGTGGAAATTTCAC	
Probe-GFP-F	AAGTTCATCTGCACCACCGGCAAG	
Probe-GFP-R	ACCATGTGATCGCGCTTCTCGTTG	

### Western blot analysis

Western blotting (WB) was performed as previously described [67]. Rabbit anti-IFNAR1 mAb (ab45172), rabbit anti-STAT1 alpha mAb (ab92506), rabbit anti-phospho-STAT1 alpha (Tyr701) mAb (ab109457), rabbit anti-STAT2 mAb (72604), rabbit anti-phospho-STAT2 (Tyr690) mAb (88410), rabbit anti-JAK1 mAb (ab133666), rabbit anti-phospho-JAK1 (Y1022+Y1023) mAb (ab138005), rabbit anti-beta actin mAb (ab8227), rabbit anti-Flag-tag polyclonal antibody (ab1162) and mouse anti-VP1 mAb (ab33990) against FCV VP1 protein were purchased from Abcam. Rabbit anti-Tyk2 mAb (9312) and rabbit anti-phospho-Tyk2 (Tyr1054/1055) mAb (68790) were purchased from CST.

### Cell viability assay

For trypan blue test, a cell suspension (3~5×10^4^/mL) is prepared. 90 μL of cell suspension is mixed with 10 μL of 0.4% trypan blue (VWR Life Science) for 3 min, and then visually examined to determine whether cells take up or exclude dye using cell counter (Cellometer, Nexcelon Bioscience). 400~600 cells were examined every time, and at least three tests were performed for a sample.

For CCK8 test, after washing with 1×PBS, a CCK8 solution (Dojindo) (20 μL) and DMEM (80 μL) were added to the cells, and the plate was incubated at 37°C for two hours. The optical density (OD) was determined by an EnSpire Multimode Plate Reader (PE, USA) under a 450 nm excitation filter.

### Indirect immunofluorescence assay (IFA) and flow cytometry analysis

The procedure of IFA had been described previously [68]. The virus was identified using a mouse anti-VP1 pAb.

After infection, the cell suspension (2×10^6^/mL) was prepared and stained by PI (BD). Then viable cells (no staining) were separated using flow cytometer. Next, surface expression of IFNAR1/2 was detected by flow cytometry analysis. A total of 1 × 10^6^ viable cells were collected and washed in PBS and incubated with 2 μL of rabbit anti-IFNAR1/2 mAb and mouse anti-VP1 mAb or isotype antibody in 100 μL of PBS for 30 min at 4°C. The cells were then washed and diluted in 200 μL of PBS containing Alexa Fluor 488-conjugated goat anti-rabbit IgG (H+L) antibody (Abcam, ab96883) and Alexa Fluor 647-conjugated goat anti-mouse IgG (H+L) antibody (Abcam, ab150115) for 30 min at 4°C. The cells were washed and diluted in 500 μL of PBS and analysed using a BD Cytomics TM FC 500 instrument. FlowJo software was used for data analysis.

### Northern blot assay

Briefly, RNA for Northern blotting (NB) was extracted using TRIzol (Life Technologies) according to the manufacturer’s instructions. RNA (20 μg) was mixed with 2×RNA loading buffer (TAKARA) and EB, denatured at 65°C for 15 minutes and then run on a 1.2% agarose/formaldehyde gel and transferred by capillary action onto a nitrocellulose membrane (Millipore). The nitrocellulose membrane was prehybridized with ExpressHyb solution (Clontech) at 42°C for 2 hours. Probes were produced using a North2South Biotin Random Prime DNA Labeling Kit (Thermo Scientific). Primers used for the production of probes are shown in Table 1. The membrane was hybridized at 42°C overnight with fresh solution containing the corresponding probe and then washed twice at room temperature for 30 min with wash solution 3 (2×SSC, 0.1% SDS) and once at 42°C for 30 min with wash solution 2 (0.1×SSC, 0.1% SDS). The membrane was then blocked with blocking buffer (catalogue #89880A; Thermo Scientific) at room temperature for 30 min. Finally, the membrane was incubated with IRDye 800-conjugated streptavidin diluted in TBST (1:2500) and imaged on an Odyssey CLx infrared imaging system (Li-COR Biosciences).

### Expression and purification of His-p30 fusion proteins

The F9 and 2280 p30 genes were cloned into the pE-SUMO vector, and the recombination plasmid was transformed into *E*. *coli* BL21 (DE3). The expression and purification procedures were performed according to a previously described method [69].

### *In vitro* degradation assay

IFNAR1 and IFNAR2 RNA containing the 5’ and 3’UTRs were synthesized by *in vitro* transcription of linearized T7 promoter-IFNAR1, -IFNAR2 and GFP using T7 *in vitro* synthesis of RNA (NEB) according to the manual. An *in vitro* assay of RNA decay was performed according to a previously described method [47]. Briefly, His-p30 fusion protein or His (10 μg) was incubated at 30°C with RNA (4 μg) in a 50 μL reaction mixture containing 25 mM Tris-HCl (pH 8.0), 80 mM potassium acetate, 1.5 mM magnesium acetate, 2 mM DTT, and 0.1 mM EDTA [47]. After the reaction was finished, the samples were extracted with phenol-chloroform-isoamylic alcohol (pH 8.0; Ambion), precipitated with ethanol, and analysed by 1.2% agarose-formaldehyde gel electrophoresis.

### Construction of recombinant full-length FCV cDNA clones

Briefly, to assemble the full-length cDNA of 2280 and F9, the genome was divided into 3 fragments (A: nt 1–2195 or 1–2799, B: nt 2196–5356 or 2800–5649, C: nt 5357–7683 or 5650–7690). In addition, the T7 promoter was added to the 5’end of fragment A by overlap PCR, and the polyadenylation signal and hepatitis delta ribozyme (HdvRz) were added to the 3’ end of fragment C. Each of the above fragments was cloned into the plasmid pOK12, which contained a new restriction enzyme linker (KpnI-ApaI-BamhI-XhoI or SalI-SacI-PstI-KpnI) to facilitate the assembly of the FCV full-length cDNA clone. Overlapping PCR was used to replace the FCV 2280 p30 gene with F9 p30. The correct fragments were cloned into BssHII/AatII-double-digested pOK-FCV 2280. A similar strategy was used to replace the FCV F9 p30 gene with 2280 p30.

### Recovery of viruses

Recovery of the recombinant viruses was carried out according to a previous report [70]. Recombinant pOK-2280 or F9, as well as their chimeric plasmids, were prepared by using the SanPrep Column Plasmid Mini-Prep Kit (Sangon Biotech) and linearized with restriction enzymes XhoI or NotI. The linearized plasmids were then transcribed into capped RNA using the HiScribe T7 High-yield RNA Synthesis Kit (NEB), and Cap analogues (Promega) and the RNeasy Mini Kit (Qiagen) were used to purify the capped RNA. The capped RNA was transfected into F81 cells (90% confluence) in 12-well plates with Lipofectamine 2000 reagent (Thermo Fisher) following the manufacturer’s protocol, and the cytopathic effect (CPE) was monitored daily. The rescued viruses were passaged once in F81 cells and harvested by freezing and thawing. Whole-genome sequencing of the recombinant viruses was performed. They were then titred and stored at -80°C.

### Analysis of pathogenicity in cats

The animal experiments were performed according to a previously described procedure [1]. Briefly, the experimental cats were negative for FCV, parvovirus, herpes virus and infectious peritonitis virus, as examined by RT-PCR or PCR as well as indirect immunofluorescence assay (IFA). Three-month-old domestic cats (n = 49) weighing from 1.5 to 1.8 kg were randomly divided into seven groups, and groups of seven cats lived in a single animal house (3 m×3 m). The cats were anaesthetized subcutaneously with Quan Mian Bao (10 mg/kg) (QFM mixture) including lidocaine, ketamine, and haloperidol. The anaesthesia SOP was carried out according to the AAHA Anesthesia Guidelines for Dogs and Cats [71]. The cats were mock infected with DMEM or inoculated with 0.5 ml (0.2 ml for each nasal passage and 0.05 ml for each eye) of 10^7^ TCID_50_ /0.5 ml via the intranasal and ocular routes. The clinical symptoms were recorded daily, and the clinical score, which included respiratory, oral cavity and eye score, were assessed on a scale of 0 to 3 (Table 2). During the clinical scoring process, we performed a double-blind (participant and assessor) manner to avoid significant bias introduced to the clinical scoring. Eye, nasal and throat swabs were collected to determine viral shedding at indicated time points. On day 5, two cats from each challenged group were euthanized by i.v. with 20% sodium pentobarbital (0.3 ml/kg) according to the protocol suggested by the World Society for the Protection of Animals, Methods for the Euthanasia of Dogs and Cats [72]. The lung samples and trachea samples were harvested for histology analysis or immediately stored at -80°C for the virus titre analysis. During the experiment, challenged cats were humanely euthanized when they were observed to suffer from pain and were not moving and lost the ability to eat and drink.

**Table 2:** Standard for assessing clinic signs.

Score	Depression and anorexia	Oral cavity symptoms	Respiratory symptoms	Ocular discharges	Lameness
0	no symptoms	no symptoms	no symptoms	no symptoms	no symptoms
1	depression	one little ulcer spot (diameter<0.5 cm)	sneezing (1–2 times per 10 min)	clear secretion (one eye)	walking posture deformation and able to bear weight on the affected foot
2	1/3-1/2 food intake	2–3 little ulcer spots (diameter<0.5 cm)	sneezing (1–3 time per 5 min)	clear secretion (two eyes)	reluctance to bear weight on the affected foot and unwillingness to place weight on the affected limb, sitting with the limb off the ground
3	apastia	big ulcer spots (diameter>1 cm)	mouth breathing and wheezing	purulent secretion	disable to bear weight on the affected foot, and trouble walking and rising

### Sequence analysis and prediction of RNA secondary structure

The alignment of sequences was performed by MEGA software, and the RNA secondary structures were produced using RNAfold WebServer (http://rna.tbi.univie.ac.at/cgi-bin/RNAWebSuite/RNAfold.cgi) by the principle of the minimum free energy.

### Ethics statement

All animal experiments were conducted according to the Guide for the Care and Use of Laboratory Animals of Harbin Veterinary Research Institute, CAAS, China. The cats were provided by the National Engineering Research Center of Veterinary Biologics CORP (Harbin, China).

### Statistics

The data are presented as the mean ± standard deviation (SD). Statistical significance was determined using unpaired t-tests in Prism 5.0 software (GraphPad Software) and a value of *p<0*.*05* was considered to indicate a significant difference. The Mann-Whitney test was used to compare clinical score values and the critical probability was taken as a *p* value of ≤0.05 for a two-sided alternative hypothesis.

## Supporting information

S1 Fig Comparison of IFN induction by FCV infection and examination of cell viability upon FCV infection. (A) CRFK cells (2×10^5^) were transfected with 200 ng/well of the reporter plasmid pIFN-Luc and with 20 ng/well of the pRLTK plasmid for 12 h. After transfection, the cells were infected with FCV 2280 or F9 at an MOI of 0.1 or 1 for 10 h, and SeV (100 HA units) was inoculated as a positive control. Luciferase assays were performed. (B) CRFK cells were infected with FCV 2280 or F9 at an MOI of 0.1 or 1 for 10 h, and SeV (100 HA units) was inoculated as a positive control. The levels of IFN-β mRNA were evaluated using qRT-PCR method. (C, D) CRFK cells infected with FCV 2280 at an MOI of 0.01, 0.1 or 1 for 18 h, then a cell suspension is prepared for the trypan blue assay (C) or the cells in the 96 well plate were mixed with CCK8 solution for the CCK8 test (D). (E) CRFK cells were mock infected (Mock) or infected with FCV 2280 at an MOI of 1 for 16 h, then the cells were fixed and the expression of FCV VP1 was analyzed by flow cytometry. The ratio of infected cells was shown. The data shown represent the mean ± SD, and all experiments were repeated three times.
(TIF)

S2 Fig Expression assay for the plasmids encoding FCV 2280 each protein as well as 2280 and F9 p30 together with their mutants. (TIF)

S3 Fig Virus titres in the swabs during infection. The data shown represent the mean ± SD.
(TIF)

S4 Fig Histology of the lung in the mock-infected and virus-infected cats. (TIF)

S5 Fig Alignment of the sequences and the predicted RNA secondary structures from the 5’UTR of IFNAR1/2. (TIF)

S1 Table Downregulated genes upon FCV 2280 infection. (DOCX)

## References

[1] Tian J, Liu D, Liu Y, Wu H, Jiang Y, et al (2016) Molecular characterization of a feline calicivirus isolated from tiger and its pathogenesis in cats. Vet Microbiol 192: 110–117. 10.1016/j.vetmic.2016.07.005 27527772

[2] Wu H, Zu S, Sun X, Liu Y, Tian J, et al (2016) N-Terminal Domain of Feline Calicivirus (FCV) Proteinase-Polymerase Contributes to the Inhibition of Host Cell Transcription. Viruses 8: 199. 10.3390/v8070199 PMC4974534 27447663

[3] Radford AD, Coyne KP, Dawson S, Porter CJ, Gaskell RM (2007) Feline calicivirus. Vet Res 38: 319–335. 10.1051/vetres:2006056 17296159

[4] Hurley KE, Pesavento PA, Pedersen NC, Poland AM, Wilson E, et al (2004) An outbreak of virulent systemic feline calicivirus disease. J Am Vet Med Assoc 224: 241–249. 10.2460/javma.2004.224.241 14736069

[5] Reynolds BS, Poulet H, Pingret JL, Jas D, Brunet S, et al (2009) A nosocomial outbreak of feline calicivirus associated virulent systemic disease in France. J Feline Med Surg 11: 633–644. 10.1016/j.jfms.2008.12.005 19201637 PMC11132575

[6] Vinje J, Estes MK, Esteves P, Green KY, Katayama K, et al (2019) ICTV Virus Taxonomy Profile: Caliciviridae. Journal of General Virology 100: 1469–1470. 10.1099/jgv.0.001332 31573467 PMC7011698

[7] Jones MK, Watanabe M, Zhu S, Graves CL, Keyes LR, et al (2014) Enteric bacteria promote human and mouse norovirus infection of B cells. Science 346: 755–759. 10.1126/science.1257147 25378626 PMC4401463

[8] Duizer E, Schwab KJ, Neill FH, Atmar RL, Koopmans MP, et al (2004) Laboratory efforts to cultivate noroviruses. J Gen Virol 85: 79–87. 10.1099/vir.0.19478-0 14718622

[9] Jones MK, Grau KR, Costantini V, Kolawole AO, de Graaf M, et al (2015) Human norovirus culture in B cells. Nat Protoc 10: 1939–1947. 10.1038/nprot.2015.121 26513671 PMC4689599

[10] Ettayebi K, Crawford SE, Murakami K, Broughman JR, Karandikar U, et al (2016) Replication of human noroviruses in stem cell-derived human enteroids. Science 353: 1387–1393. 10.1126/science.aaf5211 27562956 PMC5305121

[11] Passalacqua KD, Lu J, Goodfellow I, Kolawole AO, Arche JR, et al (2019) Glycolysis Is an Intrinsic Factor for Optimal Replication of a Norovirus. Mbio 10. 10.1128/mBio.02175-18 PMC6414699 30862747

[12] Van Dycke J, Ny A, Conceicao-Neto N, Maes J, Hosmillo M, et al (2019) A robust human norovirus replication model in zebrafish larvae. Plos Pathogens 15. 10.1371/journal.ppat.1008009 PMC6752765 31536612

[13] Wobus CE (2018) The Dual Tropism of Noroviruses. Journal of Virology 92. 10.1128/JVI.01010-17 PMC6069179 29848591

[14] Vashist S, Bailey D, Putics A, Goodfellow I (2009) Model systems for the study of human norovirus Biology. Future Virol 4: 353–367. 10.2217/fvl.09.18 21516251 PMC3079900

[15] Conley MJ, McElwee M, Azmi L, Gabrielsen M, Byron O, et al (2019) Calicivirus VP2 forms a portal-like assembly following receptor engagement. Nature 565: 377–381. 10.1038/s41586-018-0852-1 30626974

[16] Stark GR, Darnell JE (2012) The JAK-STAT Pathway at Twenty. Immunity 36: 503–514. 10.1016/j.immuni.2012.03.013 22520844 PMC3909993

[17] Brierley MM, Fish EN (2002) IFN-alpha/beta receptor interactions to biologic outcomes: Understanding the circuitry. Journal of Interferon and Cytokine Research 22: 835–845. 10.1089/107999002760274845 12396722

[18] Samuel CE (2001) Antiviral actions of interferons. Clin Microbiol Rev 14: 778–809, table of contents. 10.1128/CMR.14.4.778-809.2001 11585785 PMC89003

[19] Schindler C, Levy DE, Decker T (2007) JAK-STAT signaling: from interferons to cytokines. J Biol Chem 282: 20059–20063. 10.1074/jbc.R700016200 17502367

[20] Portugal R, Leitao A, Martins C (2018) Modulation of type I interferon signaling by African swine fever virus (ASFV) of different virulence L60 and NHV in macrophage host cells. Vet Microbiol 216: 132–141. 10.1016/j.vetmic.2018.02.008 29519508

[21] Ding Z, Fang LR, Jing HY, Zeng SL, Wang D, et al (2014) Porcine Epidemic Diarrhea Virus Nucleocapsid Protein Antagonizes Beta Interferon Production by Sequestering the Interaction between IRF3 and TBK1. Journal of Virology 88: 8936–8945. 10.1128/JVI.00700-14 24872591 PMC4136253

[22] Georgana I, Sumner RP, Towers GJ, Maluquer de Motes C (2018) Virulent poxviruses inhibit DNA sensing by preventing STING activation. J Virol. 10.1128/JVI.02145-17 PMC5923072 29491158

[23] Yi C, Zhao Z, Wang S, Sun X, Zhang D, et al (2017) Influenza A Virus PA Antagonizes Interferon-β by Interacting with Interferon Regulatory Factor 3. Frontiers in Immunology 8: 1051 10.3389/fimmu.2017.01051 28955326 PMC5600993

[24] Wang J, Lei CQ, Ji YH, Zhou HB, Ren YJ, et al (2016) Duck Tembusu Virus Nonstructural Protein 1 Antagonizes IFN-beta Signaling Pathways by Targeting VISA. Journal of Immunology 197: 4704–4713. 10.4049/jimmunol.1502317 27821666

[25] Fleming SB (2016) Viral Inhibition of the IFN-Induced JAK/STAT Signalling Pathway: Development of Live Attenuated Vaccines by Mutation of Viral-Encoded IFN-Antagonists. Vaccines 4. 10.3390/vaccines4030023 PMC5041017 27367734

[26] Zhu X, Wang D, Zhou J, Pan T, Chen J, et al (2017) Porcine Deltacoronavirus nsp5 Antagonizes Type I Interferon Signaling by Cleaving STAT2. J Virol 91. 10.1128/JVI.00003-17 PMC5411617 28250121

[27] Guo LJ, Luo XL, Li R, Xu YF, Zhang J, et al (2016) Porcine Epidemic Diarrhea Virus Infection Inhibits Interferon Signaling by Targeted Degradation of STAT1. Journal of Virology 90: 8281–8292. 10.1128/JVI.01091-16 27384656 PMC5008104

[28] Hung HC, Wang HC, Shih SR, Teng IF, Tseng CP, et al (2011) Synergistic Inhibition of Enterovirus 71 Replication by Interferon and Rupintrivir. Journal of Infectious Diseases 203: 1784–1790. 10.1093/infdis/jir174 21536800

[29] Lin RJ, Liao CL, Lin E, Lin YL (2004) Blocking of the alpha interferon-induced Jak-Stat signaling pathway by Japanese encephalitis virus infection. Journal of Virology 78: 9285–9294. 10.1128/JVI.78.17.9285-9294.2004 15308723 PMC506928

[30] Symons JA, Alcami A, Smith GL (1995) Vaccinia Virus Encodes a Soluble Type-I Interferon Receptor of Novel Structure and Broad Species-Specificity. Cell 81: 551–560. 10.1016/0092-8674(95)90076-4 7758109

[31] Upton C, Mossman K, McFadden G (1992) Encoding of a homolog of the IFN-gamma receptor by myxoma virus. Science 258: 1369–1372. 10.1126/science.1455233 1455233

[32] Yuan H, You J, You H, Zheng C (2018) Herpes Simplex Virus 1 UL36USP Antagonizes Type I Interferon-Mediated Antiviral Innate Immunity. J Virol 92. 10.1128/JVI.01161-18 PMC6146802 29997210

[33] Zhang R, Xu AT, Qin C, Zhang Q, Chen SF, et al (2017) Pseudorabies Virus dUTPase UL50 Induces Lysosomal Degradation of Type I Interferon Receptor 1 and Antagonizes the Alpha Interferon Response. Journal of Virology 91. 10.1128/JVI.01148-17 PMC5640830 28794045

[34] Gaglia MM, Covarrubias S, Wong W, Glaunsinger BA (2012) A Common Strategy for Host RNA Degradation by Divergent Viruses. Journal of Virology 86: 9527–9530. 10.1128/JVI.01230-12 22740404 PMC3416159

[35] Covarrubias S, Gaglia MM, Kumar GR, Wong W, Jackson AO, et al (2011) Coordinated destruction of cellular messages in translation complexes by the gammaherpesvirus host shutoff factor and the mammalian exonuclease Xrn1. PLoS Pathog 7: e1002339 10.1371/journal.ppat.1002339 22046136 PMC3203186

[36] Rowe M, Glaunsinger B, van Leeuwen D, Zuo JM, Sweetman D, et al (2007) Host shutoff during productive Epstein-Barr virus infection is mediated by BGLF5 and may contribute to immune evasion. Proceedings of the National Academy of Sciences of the United States of America 104: 3366–3371. 10.1073/pnas.0611128104 17360652 PMC1805610

[37] Kamitani W, Narayanan K, Huang C, Lokugamage K, Ikegami T, et al (2006) Severe acute respiratory syndrome coronavirus nsp1 protein suppresses host gene expression by promoting host mRNA degradation. Proceedings of the National Academy of Sciences of the United States of America 103: 12885–12890. 10.1073/pnas.0603144103 16912115 PMC1568942

[38] Kwong AD, Kruper JA, Frenkel N (1988) Herpes simplex virus virion host shutoff function. J Virol 62: 912–921. 10.1128/JVI.62.3.912-921.1988 2828686 PMC253650

[39] Bidawid S, Malik N, Adegbunrin O, Sattar SA, Farber JM (2003) A feline kidney cell line-based plaque assay for feline calicivirus, a surrogate for Norwalk virus. Journal of Virological Methods 107: 163–167. 10.1016/s0166-0934(02)00214-8 12505630

[40] Coyne KP, Gaskell RM, Dawson S, Porter CJ, Radford AD (2007) Evolutionary mechanisms of persistence and diversification of a calicivirus within endemically infected natural host populations. Journal of Virology 81: 1961–1971. 10.1128/JVI.01981-06 17151126 PMC1797550

[41] Radford AD, Addie D, Belak S, Boucraut-Baralon C, Egberink H, et al (2009) FELINE CALICIVIRUS INFECTION ABCD guidelines on prevention and management. Journal of Feline Medicine and Surgery 11: 556–564. 10.1016/j.jfms.2009.05.004 19481035 PMC11132273

[42] Tian J, Zhang X, Wu H, Liu C, Liu J, et al (2015) Assessment of the IFN-beta response to four feline caliciviruses: Infection in CRFK cells. Infect Genet Evol 34: 352–360. 10.1016/j.meegid.2015.06.003 26051884

[43] Aaronson DS, Horvath CM (2002) A road map for those who don't know JAK-STAT. Science 296: 1653–1655. 10.1126/science.1071545 12040185

[44] Platanias LC (2005) Mechanisms of type-I- and type-II-interferon-mediated signalling. Nature Reviews Immunology 5: 375–386. 10.1038/nri1604 15864272

[45] Sobell HM (1985) Actinomycin and DNA transcription. Proceedings of the National Academy of Sciences of the United States of America 82: 5328–5331. 10.1073/pnas.82.16.5328 2410919 PMC390561

[46] Covarrubias S, Richner JM, Clyde K, Lee YJ, Glaunsinger BA (2009) Host Shutoff Is a Conserved Phenotype of Gammaherpesvirus Infection and Is Orchestrated Exclusively from the Cytoplasm. Journal of Virology 83: 9554–9566. 10.1128/JVI.01051-09 19587049 PMC2738246

[47] Taddeo B, Zhang W, Roizman B (2006) The U(L)41 protein of herpes simplex virus 1 degrades RNA by endonucleolytic cleavage in absence of other cellular or viral proteins. Proc Natl Acad Sci U S A 103: 2827–2832. 10.1073/pnas.0510712103 16477041 PMC1413801

[48] Raftery N, Stevenson NJ (2017) Advances in anti-viral immune defence: revealing the importance of the IFN JAK/STAT pathway. Cellular and Molecular Life Sciences 74: 2525–2535. 10.1007/s00018-017-2520-2 28432378 PMC7079803

[49] Yumiketa Y, Narita T, Inoue Y, Sato G, Kamitani W, et al (2016) Nonstructural protein p39 of feline calicivirus suppresses host innate immune response by preventing IRF-3 activation. Vet Microbiol 185: 62–67. 10.1016/j.vetmic.2016.02.005 26931393

[50] McFadden N, Bailey D, Carrara G, Benson A, Chaudhry Y, et al (2011) Norovirus regulation of the innate immune response and apoptosis occurs via the product of the alternative open reading frame 4. PLoS Pathog 7: e1002413 10.1371/journal.ppat.1002413 22174679 PMC3234229

[51] Lubick Kirk, nbsp Robertson, Shelly, et al (2015) Flavivirus Antagonism of Type I Interferon Signaling Reveals Prolidase as a Regulator of IFNAR1 Surface Expression. Cell Host & Microbe 18: 61–74. 26159719 10.1016/j.chom.2015.06.007 PMC4505794

[52] Evans JD, Crown RA, Sohn JA, Seeger C (2011) West Nile virus infection induces depletion of IFNAR1 protein levels. Viral Immunol 24: 253–263. 10.1089/vim.2010.0126 21830897 PMC3154464

[53] Patel D, Nan YC, Shen MY, Ritthipichai K, Zhu XP, et al (2011) Porcine Reproductive and Respiratory Syndrome Virus Inhibits Type I Interferon Signaling by Blocking STAT1/STAT2 Nuclear Translocation (vol 84, pg 11045, 2010). Journal of Virology 85: 5705–5705. 10.1128/JVI.00655-10 PMC2953160 20739522

[54] Royall E, Locker N (2016) Translational Control during Calicivirus Infection. Viruses-Basel 8. 10.3390/v8040104 PMC4848598 27104553

[55] Willcocks MM, Carter MJ, Roberts LO (2004) Cleavage of eukaryotic initiation factor eIF4G and inhibition of host-cell protein synthesis during feline calicivirus infection. J Gen Virol 85: 1125–1130. 10.1099/vir.0.19564-0 15105529

[56] Kuyumcu-Martinez M, Belliot G, Sosnovtsev SV, Chang KO, Green KY, et al (2004) Calicivirus 3C-like proteinase inhibits cellular translation by cleavage of poly(A)-binding protein. J Virol 78: 8172–8182. 10.1128/JVI.78.15.8172-8182.2004 15254188 PMC446144

[57] Cao S, Dhungel P, Yang ZL (2017) Going against the Tide: Selective Cellular Protein Synthesis during Virally Induced Host Shutoff. Journal of Virology 91. 10.1128/JVI.00071-17 PMC5553178 28637757

[58] Hutin S, Lee Y, Glaunsinger BA (2013) An RNA element in human interleukin 6 confers escape from degradation by the gammaherpesvirus SOX protein. J Virol 87: 4672–4682. 10.1128/JVI.00159-13 23408619 PMC3624381

[59] Hartenian E, Gilbertson S, Federspiel JD, Cristea IM, Glaunsinger BA (2020) RNA decay during gammaherpesvirus infection reduces RNA polymerase II occupancy of host promoters but spares viral promoters. PLoS Pathog 16: e1008269 10.1371/journal.ppat.1008269 32032393 PMC7032723

[60] Jiang ZT, Su CH, Zheng CF (2016) Herpes Simplex Virus 1 Tegument Protein UL41 Counteracts IFIT3 Antiviral Innate Immunity. Journal of Virology 90: 11056–11061. 10.1128/JVI.01672-16 27681138 PMC5126364

[61] Shen GH, Wang KZ, Wang S, Cai MS, Li ML, et al (2014) Herpes Simplex Virus 1 Counteracts Viperin via Its Virion Host Shutoff Protein UL41. Journal of Virology 88: 12163–12166. 10.1128/JVI.01380-14 25078699 PMC4178720

[62] Kadoi K, Kiryu M, Iwabuchi M, Kamata H, Yukawa M, et al (1997) A strain of calicivirus isolated from lions with vesicular lesions on tongue and snout. New Microbiologica 20: 141–148. 9208424

[63] Pedersen NC, Elliott JB, Glasgow A, Poland A, Keel K (2000) An isolated epizootic of hemorrhagic-like fever in cats caused by a novel and highly virulent strain of feline calicivirus. Veterinary Microbiology 73: 281 10.1016/s0378-1135(00)00183-8 10781727 PMC7117377

[64] Sun Y, Luo G, Zhao L, Huang L, Qin Y, et al (2018) Integration of RNAi and RNA-seq Reveals the Immune Responses of Epinephelus coioides to sigX Gene of Pseudomonas plecoglossicida. Front Immunol 9: 1624 10.3389/fimmu.2018.01624 30061893 PMC6054955

[65] Wang Y, Tao X, Tang XM, Xiao L, Sun JL, et al (2013) Comparative transcriptome analysis of tomato (Solanum lycopersicum) in response to exogenous abscisic acid. BMC Genomics 14: 841 10.1186/1471-2164-14-841 24289302 PMC4046761

[66] Reed LJ, Muench H (1938) A SIMPLE METHOD OF ESTIMATING FIFTY PER CENT ENDPOINTS. Amjhyg 27.

[67] Tian J, Liu Y, Liu X, Sun X, Zhang J, et al (2018) Feline Herpesvirus 1 US3 Blocks the Type I Interferon Signal Pathway by Targeting Interferon Regulatory Factor 3 Dimerization in a Kinase-Independent Manner. J Virol 92. 10.1128/JVI.00047-18 PMC5974484 29618645

[68] Wu H, Zhang X, Liu C, Liu D, Liu J, et al (2015) Antiviral effect of lithium chloride on feline calicivirus in vitro. Arch Virol 160: 2935–2943. 10.1007/s00705-015-2534-8 26239340 PMC7086906

[69] Zuo X, Mattern MR, Tan R, Li SS, Hall J, et al (2005) Expression and purification of SARS coronavirus proteins using SUMO-fusions. Protein Expression and Purification 42: 100–110. 10.1016/j.pep.2005.02.004 15939295 PMC7129641

[70] Abente EJ, Sosnovtsev SV, Sandoval-Jaime C, Parra GI, Bok K, et al (2013) The feline calicivirus leader of the capsid protein is associated with cytopathic effect. J Virol 87: 3003–3017. 10.1128/JVI.02480-12 23269802 PMC3592120

[71] Bednarski R, Grimm K, Harvey R, Lukasik VM, Penn WS, et al (2011) AAHA anesthesia guidelines for dogs and cats. J Am Anim Hosp Assoc 47: 377–385. 10.5326/JAAHA-MS-5846 22058343

[72] World Society for the Protection of Animals Methods for the euthanasia of dogs and cats: comparision and recommendations. http://www.icam-coalition.org/downloads/Methods%20for%20the%20euthanasia%20of%20dogs%20and%20cats-%20English.pdf.

