# Supplementary figures and images for: Feline calicivirus strain 2280 p30 antagonizes type I interferon-mediated antiviral innate immunity through directly degrading IFNAR1 mRNA
Source: PLoS Pathog. 2020 Oct 19;16(10):e1008944. doi: 10.1371/journal.ppat.1008944 (PMC7571719; doi:10.1371/journal.ppat.1008944)

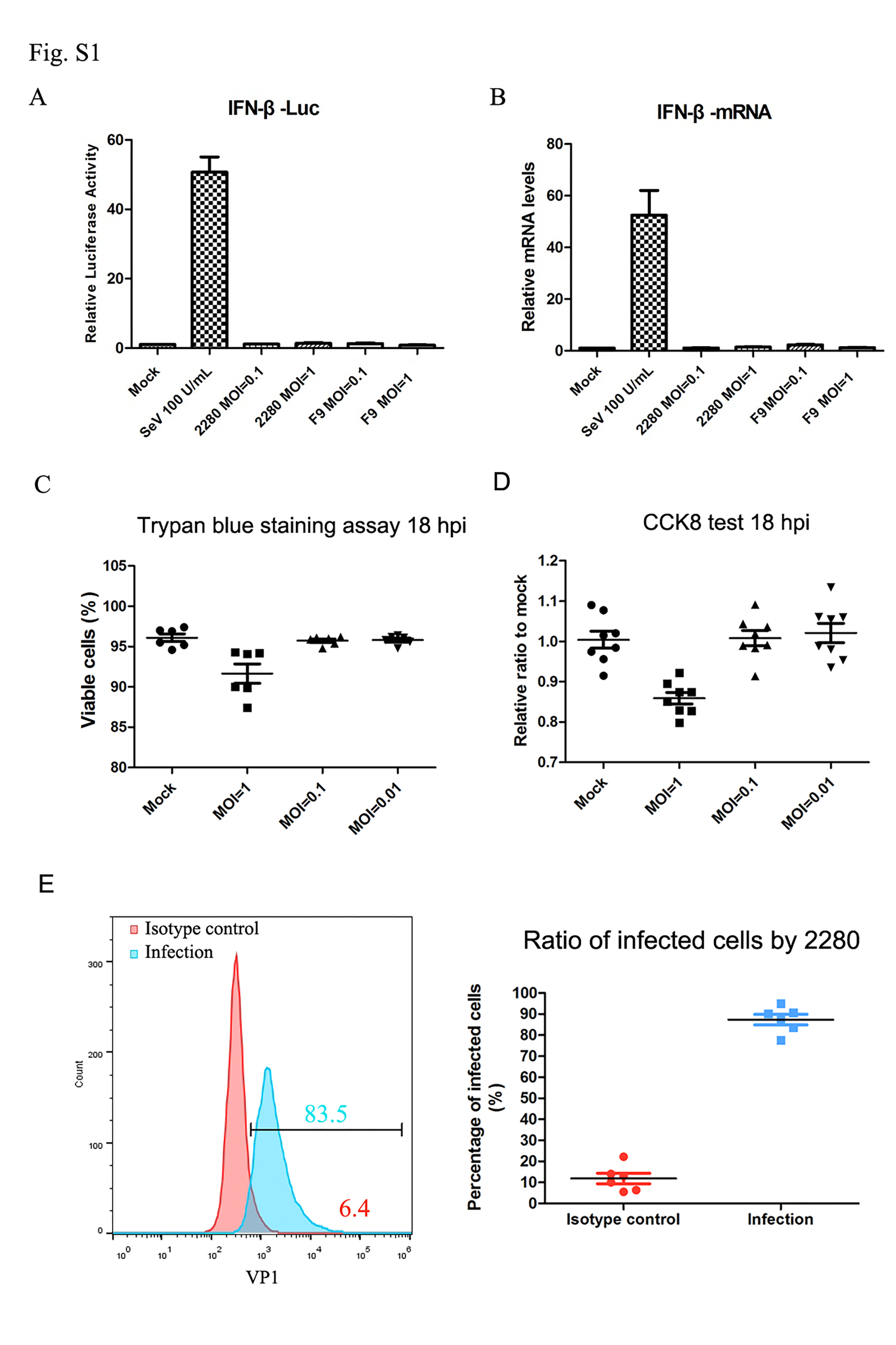

Supplement: S1 Fig — (A) CRFK cells (2×10^5^) were transfected with 200 ng/well of the reporter plasmid pIFN-Luc and with 20 ng/well of the pRLTK plasmid for 12 h. After transfection, the cells were infected with FCV 2280 or F9 at an MOI of 0.1 or 1 for 10 h, and SeV (100 HA units) was inoculated as a positive control. Luciferase assays were performed. (B) CRFK cells were infected with FCV 2280 or F9 at an MOI of 0.1 or 1 for 10 h, and SeV (100 HA units) was inoculated as a positive control. The levels of IFN-β mRNA were evaluated using qRT-PCR method. (C, D) CRFK cells infected with FCV 2280 at an MOI of 0.01, 0.1 or 1 for 18 h, then a cell suspension is prepared for the trypan blue assay (C) or the cells in the 96 well plate were mixed with CCK8 solution for the CCK8 test (D). (E) CRFK cells were mock infected (Mock) or infected with FCV 2280 at an MOI of 1 for 16 h, then the cells were fixed and the expression of FCV VP1 was analyzed by flow cytometry. The ratio of infected cells was shown. The data shown represent the mean ± SD, and all experiments were repeated three times. (TIF) [file ppat.1008944.s001.tif]

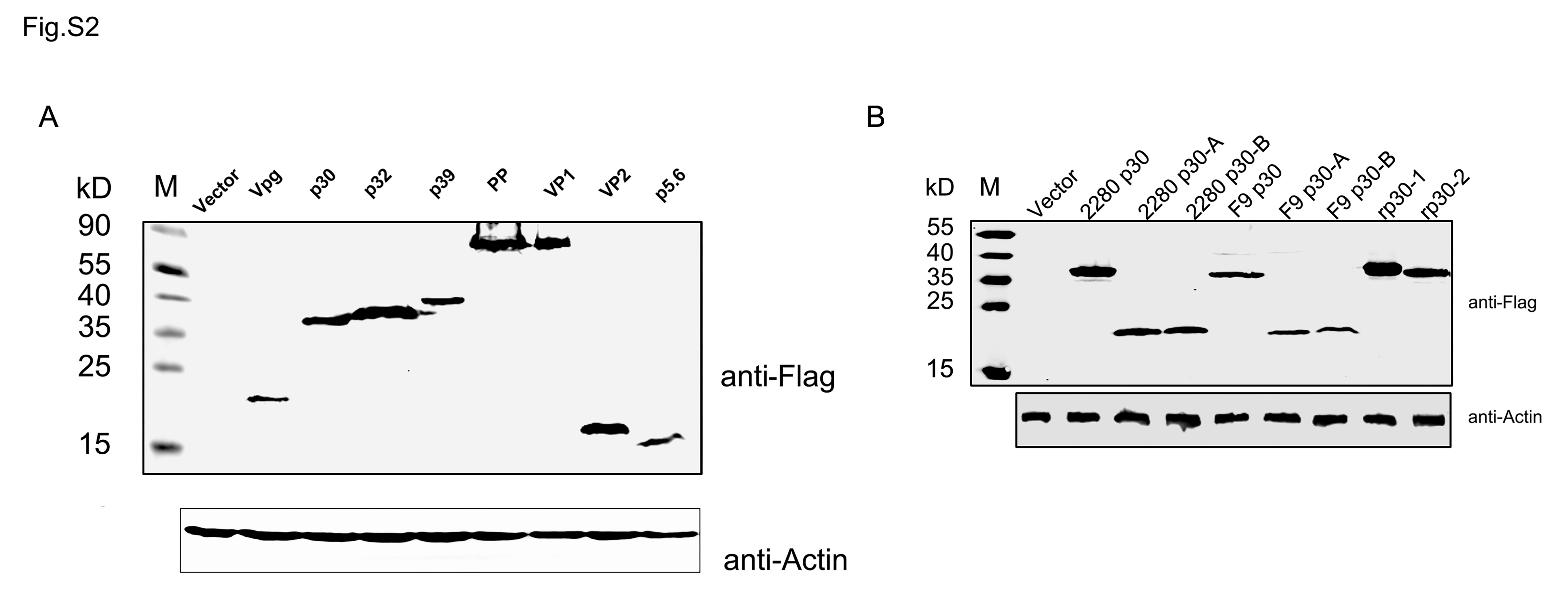

Supplement: S2 Fig — (TIF) [file ppat.1008944.s002.tif]

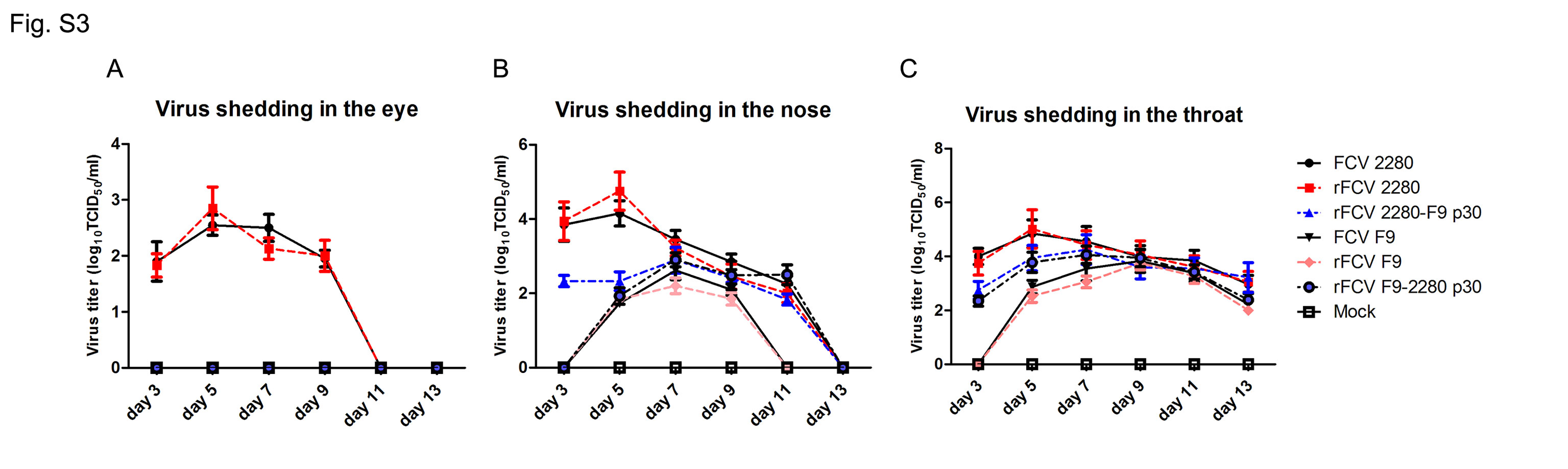

Supplement: S3 Fig — The data shown represent the mean ± SD. (TIF) [file ppat.1008944.s003.tif]

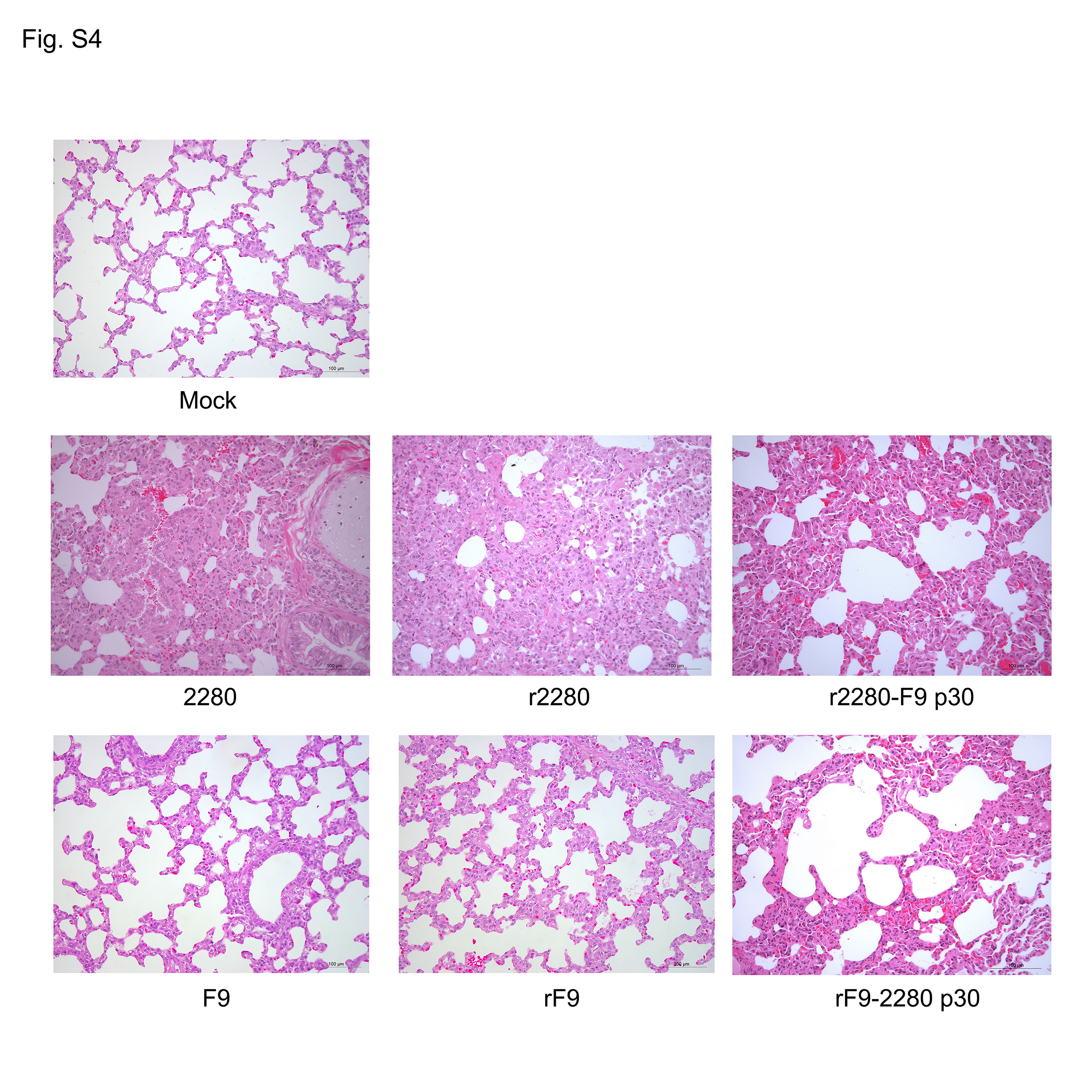

Supplement: S4 Fig — (TIF) [file ppat.1008944.s004.tif]

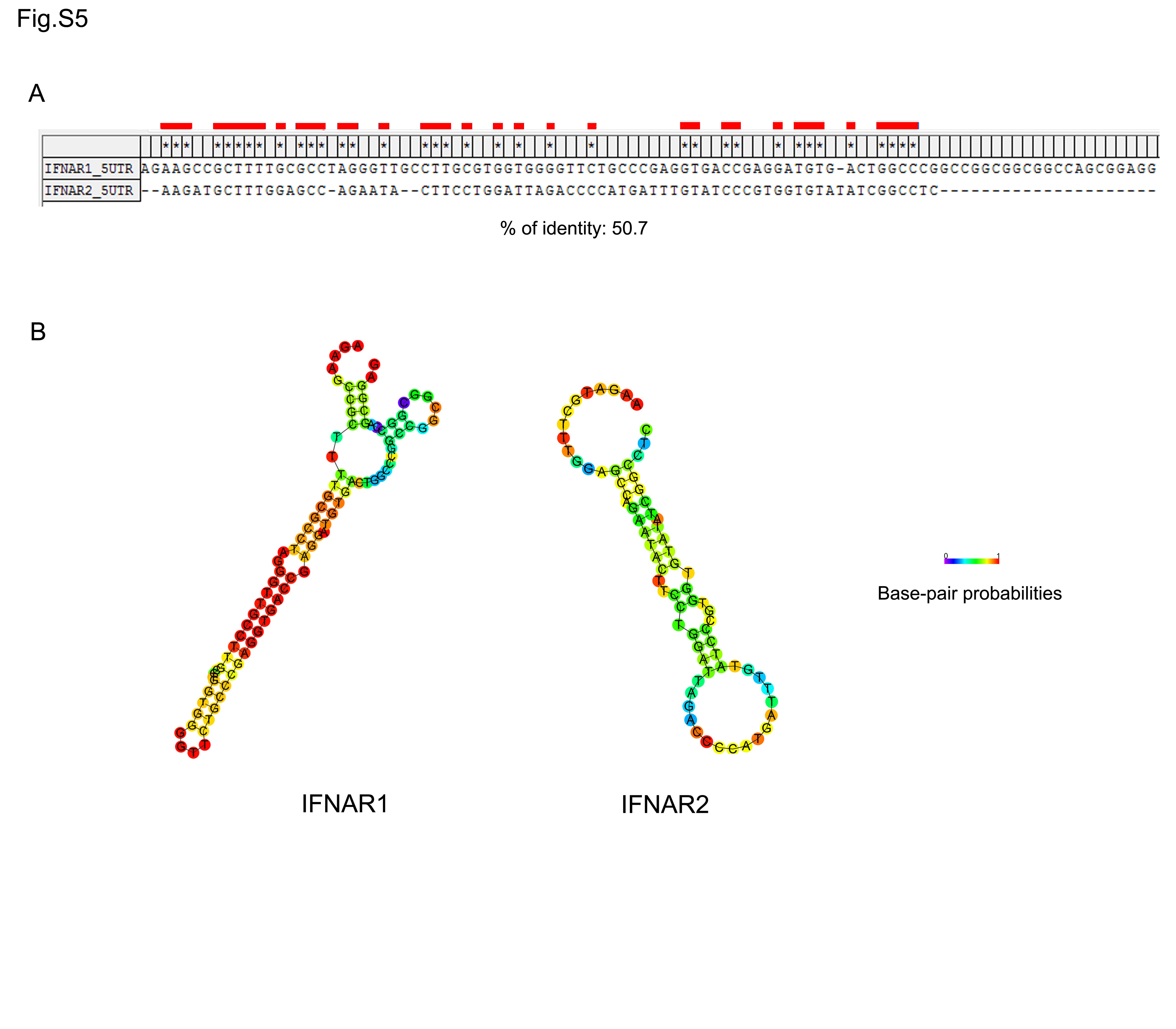

Supplement: S5 Fig — (TIF) [file ppat.1008944.s005.tif]
