# Supplementary material for: Feline calicivirus strain 2280 p30 antagonizes type I interferon-mediated antiviral innate immunity through directly degrading IFNAR1 mRNA
Source: PLoS Pathog. 2020 Oct 19;16(10):e1008944. doi: 10.1371/journal.ppat.1008944 (PMC7571719; doi:10.1371/journal.ppat.1008944)
Supplement: S1 Table — (DOCX) [file ppat.1008944.s006.docx]

Table. S1 Downregulated genes upon FCV 2280 infection

| Gene  name | Mock_1  Read count^a^ | Mock_2  Read count^a^ | FCV_12hpi_1  Read count^a^ | FCV_12hpi_2  Read count^a^ | Mock_Aver  Read count | FCV_12hpi_Aver  Read count | FC ^b^ (FCV_12hpi/Mock) | Log_2_FC(FCV_12hpi/Mock) | P-value | P-adjust | Significant^c^ |
| --- | --- | --- | --- | --- | --- | --- | --- | --- | --- | --- | --- |
|  |  |  |  |  |  |  |  |  |  |  |  |
| CYP1A1 | 745.398 | 705.418 | 65.378 | 68.578 | 725.408 | 66.978 | 0.095 | -3.397 | 0 | 0 | yes |
| CHST8 | 16.175 | 13.15 | 1.817 | 2.399 | 14.663 | 2.108 | 0.212 | -2.24 | 1.486E-47 | 2.801E-45 | yes |
| ACTC1 | 298.91 | 268.98 | 65.32 | 65.59 | 283.945 | 65.455 | 0.234 | -2.096 | 0 | 0 | yes |
| FRMD3 | 3.437 | 2.969 | 0.752 | 0.55 | 3.203 | 0.651 | 0.287 | -1.803 | 5.189E-30 | 5.373E-28 | yes |
| ANKH | 68.227 | 63.249 | 21.659 | 20.421 | 65.738 | 21.04 | 0.321 | -1.638 | 2.219E-291 | 8.5E-288 | yes |
| SRSF6 | 58.113 | 54.547 | 22.412 | 21.28 | 56.33 | 21.846 | 0.385 | -1.377 | 4.249E-172 | 4.44E-169 | yes |
| PDE9A | 30.487 | 23.984 | 9.975 | 10.105 | 27.236 | 10.04 | 0.4 | -1.322 | 3.739E-34 | 4.671E-32 | yes |
| SERPINE1 | 260.573 | 226.191 | 101.262 | 99.554 | 243.382 | 100.408 | 0.414 | -1.274 | 4.462E-185 | 5.129E-182 | yes |
| IFNAR1 | 69.238 | 64.445 | 27.186 | 28.117 | 66.8415 | 27.6515 | 0.414 | -1.273 | 2.92E-71 | 1.35E-69 | yes |
| MAT2A | 68.279 | 61.4 | 28.586 | 28.437 | 64.84 | 28.511 | 0.447 | -1.163 | 3.908E-73 | 1.453E-70 | yes |
| CRISPLD2 | 2.83 | 2.307 | 0.576 | 0.67 | 2.569 | 0.623 | 0.453 | -1.141 | 3.02E-09 | 6.134E-08 | yes |
| BSPRY | 3.221 | 2.871 | 0.742 | 0.96 | 3.046 | 0.851 | 0.46 | -1.12 | 5.556E-09 | 1.082E-07 | yes |
| ESM1 | 127.318 | 114.147 | 58.755 | 55.275 | 120.733 | 57.015 | 0.475 | -1.075 | 1.595E-94 | 9.165E-92 | yes |
| KIF26B | 4.116 | 3.631 | 1.915 | 1.619 | 3.873 | 1.767 | 0.497 | -1.008 | 8.053E-18 | 4.305E-16 | yes |
| AREG | 165.892 | 143.494 | 73.36 | 77.284 | 154.693 | 75.322 | 0.498 | -1.007 | 2.564E-53 | 5.668E-51 | yes |
| TMEM200A | 270.173 | 261.222 | 133.981 | 132.149 | 265.697 | 133.065 | 0.504 | -0.9896 | 2.04E-109 | 1.465E-106 | no |
| GTPBP4 | 32.853 | 29.23 | 14.547 | 16.153 | 31.041 | 15.35 | 0.509 | -0.9747 | 7.514E-29 | 7.138E-27 | no |
| DKK1 | 9.651 | 8.079 | 3.185 | 3.288 | 8.865 | 3.236 | 0.511 | -0.9686 | 1.098E-07 | 0.000001705 | no |
| STC1 | 55.253 | 49.291 | 26.681 | 26.738 | 52.272 | 26.709 | 0.512 | -0.9661 | 4.96E-73 | 1.782E-70 | no |
| CARD10 | 156.364 | 141.304 | 73.79 | 80.193 | 148.834 | 76.992 | 0.516 | -0.9537 | 1.11E-100 | 7.085E-98 | no |
| CYP1B1 | 122.595 | 108.53 | 50.88 | 52.316 | 115.563 | 51.598 | 0.522 | -0.9373 | 9.329E-137 | 8.935E-134 | no |
| TXNRD1 | 64.266 | 59.726 | 33.236 | 31.895 | 61.996 | 32.566 | 0.525 | -0.9298 | 5.262E-78 | 2.24E-75 | no |
| RELB | 44.439 | 31.644 | 19.256 | 16.283 | 38.041 | 17.77 | 0.525 | -0.9308 | 1.102E-13 | 3.922E-12 | no |
| TFRC | 89.063 | 85.754 | 45.009 | 47.638 | 87.409 | 46.323 | 0.527 | -0.9251 | 8.229E-122 | 6.756E-119 | no |
| HBEGF | 51.754 | 45.349 | 24.043 | 26.278 | 48.551 | 25.16 | 0.527 | -0.9254 | 1.9E-35 | 2.569E-33 | no |
| EGR1 | 13.479 | 10.532 | 5.803 | 6.127 | 12.005 | 5.965 | 0.528 | -0.9208 | 1.492E-15 | 6.569E-14 | no |
| PEG10 | 20.002 | 19.613 | 9.985 | 10.865 | 19.807 | 10.425 | 0.53 | -0.917 | 6.649E-45 | 1.194E-42 | no |
| TRAF1 | 4.229 | 3.845 | 1.768 | 1.909 | 4.037 | 1.839 | 0.531 | -0.9125 | 5.865E-09 | 1.141E-07 | no |
| CLDND1 | 709.448 | 665.88 | 373.483 | 377.546 | 687.664 | 375.514 | 0.532 | -0.9108 | 3.757E-237 | 1.08E-233 | no |
| FNDC3B | 39.438 | 39.451 | 20.848 | 21.55 | 39.445 | 21.199 | 0.534 | -0.904 | 1.71E-80 | 8.19E-78 | no |
| KLF10 | 20.002 | 18.338 | 10.248 | 10.125 | 19.17 | 10.186 | 0.547 | -0.8694 | 9.722E-21 | 6.278E-19 | no |
| GEMIN4 | 3.756 | 4.497 | 1.964 | 1.959 | 4.127 | 1.962 | 0.548 | -0.8668 | 3.045E-08 | 5.155E-07 | no |
| IFRD1 | 23.408 | 20.197 | 11.714 | 11.695 | 21.803 | 11.704 | 0.549 | -0.8662 | 1.824E-23 | 1.379E-21 | no |
| GNB1L | 82.385 | 73.324 | 40.534 | 42.091 | 77.855 | 41.313 | 0.559 | -0.8385 | 1.028E-69 | 3.475E-67 | no |
| NOP2 | 49.522 | 42.896 | 26.261 | 24.379 | 46.209 | 25.32 | 0.56 | -0.8371 | 1.945E-29 | 1.944E-27 | no |
| PPP1R18 | 4.867 | 4.215 | 2.413 | 2.089 | 4.541 | 2.251 | 0.564 | -0.8261 | 6.291E-08 | 0.000001014 | no |
| DIEXF | 10.042 | 9.675 | 5.158 | 5.477 | 9.858 | 5.318 | 0.564 | -0.8257 | 1.13E-13 | 4.01E-12 | no |
| SH2B3 | 14.991 | 13.54 | 8.402 | 7.167 | 14.265 | 7.784 | 0.566 | -0.8222 | 9.849E-22 | 6.699E-20 | no |
| RIN1 | 19.292 | 17.024 | 10.258 | 10.105 | 18.158 | 10.181 | 0.566 | -0.8212 | 9.666E-30 | 9.832E-28 | no |
| DNAJA1 | 172.57 | 163.749 | 94.042 | 96.865 | 168.159 | 95.453 | 0.566 | -0.8217 | 1.556E-94 | 9.165E-92 | no |
| ID1 | 38.595 | 36.628 | 21.933 | 16.992 | 37.611 | 19.462 | 0.567 | -0.8177 | 3.204E-10 | 7.531E-09 | no |
| ACTB | 1975.056 | 1788.25 | 1076.955 | 1079.953 | 1881.653 | 1078.454 | 0.571 | -0.8088 | 2.828E-185 | 3.611E-182 | no |
| GMPR | 92.726 | 84.78 | 48.78 | 51.506 | 88.753 | 50.143 | 0.572 | -0.8049 | 4.988E-33 | 5.91E-31 | no |
| ZNF335 | 11.359 | 10.172 | 6.292 | 5.837 | 10.765 | 6.064 | 0.574 | -0.7997 | 1.173E-19 | 7.134E-18 | no |
| MFSD2A | 20.619 | 18.591 | 10.522 | 11.115 | 19.605 | 10.819 | 0.575 | -0.7984 | 1.033E-13 | 3.688E-12 | no |
| ADGRF4 | 16.792 | 18.075 | 9.633 | 9.266 | 17.434 | 9.45 | 0.575 | -0.799 | 3.16E-15 | 1.335E-13 | no |
| TIMP3 | 25.116 | 24.062 | 14 | 14.253 | 24.589 | 14.127 | 0.577 | -0.7936 | 1.471E-33 | 1.799E-31 | no |
| ZNF423 | 5.278 | 4.896 | 2.843 | 2.739 | 5.087 | 2.791 | 0.578 | -0.792 | 3.776E-11 | 9.863E-10 | no |
| TM4SF1 | 50.746 | 39.626 | 25.841 | 24.819 | 45.186 | 25.33 | 0.578 | -0.7898 | 1.403E-17 | 7.43E-16 | no |
| RBM6 | 9.61 | 8.332 | 4.787 | 5.088 | 8.971 | 4.938 | 0.579 | -0.7895 | 3.871E-12 | 1.141E-10 | no |
| BDKRB2 | 11.802 | 9.5 | 5.608 | 6.037 | 10.651 | 5.822 | 0.58 | -0.7851 | 5.801E-11 | 1.463E-09 | no |
| SRXN1 | 96.718 | 89.589 | 54.475 | 52.686 | 93.154 | 53.581 | 0.582 | -0.7812 | 5.456E-48 | 1.045E-45 | no |
| TULP4 | 21.885 | 23.916 | 13.355 | 13.214 | 22.901 | 13.285 | 0.583 | -0.7784 | 1.679E-27 | 1.544E-25 | no |
| PIM3 | 24.55 | 20.528 | 12.534 | 12.264 | 22.539 | 12.399 | 0.583 | -0.7789 | 1.154E-10 | 2.847E-09 | no |
| PLAUR | 59.348 | 52.61 | 32.005 | 31.196 | 55.979 | 31.601 | 0.584 | -0.7755 | 1.871E-19 | 1.114E-17 | no |
| DDX21 | 68.546 | 64.641 | 39.899 | 38.642 | 66.594 | 39.27 | 0.587 | -0.769 | 3.904E-67 | 1.181E-64 | no |
| SLC35F4 | 32.174 | 29.045 | 18.396 | 19.561 | 30.61 | 18.979 | 0.589 | -0.7638 | 1.45E-36 | 2.008E-34 | no |
| C15orf39 | 7.83 | 7.68 | 4.387 | 4.518 | 7.755 | 4.452 | 0.59 | -0.7624 | 1.85E-12 | 5.611E-11 | no |
| PFKFB3 | 5.721 | 6.045 | 3.498 | 2.999 | 5.883 | 3.248 | 0.592 | -0.7575 | 1.015E-08 | 0.000000187 | no |
| ZYX | 184.979 | 162.085 | 103.353 | 102.443 | 173.532 | 102.898 | 0.593 | -0.7527 | 2.678E-55 | 6.034E-53 | no |
| CD200 | 11.318 | 9.743 | 5.832 | 5.677 | 10.53 | 5.755 | 0.593 | -0.7539 | 1.547E-08 | 2.766E-07 | no |
| NMD3 | 13.438 | 12.118 | 6.497 | 8.096 | 12.778 | 7.296 | 0.594 | -0.7515 | 1.949E-11 | 5.208E-10 | no |
| NRG1 | 4.836 | 5.198 | 2.833 | 2.109 | 5.017 | 2.471 | 0.595 | -0.7498 | 0.00002306 | 0.0002152 | no |
| TNIP3 | 2.428 | 2.258 | 0.899 | 1.369 | 2.343 | 1.134 | 0.596 | -0.7462 | 0.00002902 | 0.0002653 | no |
| SLC7A11 | 5.083 | 4.945 | 2.833 | 3.029 | 5.014 | 2.931 | 0.597 | -0.7442 | 7.792E-15 | 3.143E-13 | no |
| NAB2 | 11.874 | 12.05 | 7.122 | 6.707 | 11.962 | 6.915 | 0.597 | -0.7453 | 7E-12 | 2.006E-10 | no |
| HSPA8 | 2631.34 | 2417.414 | 1501.026 | 1530.906 | 2524.377 | 1515.966 | 0.597 | -0.7451 | 6.444E-200 | 1.235E-196 | no |
| SPOUT1 | 9.744 | 8.945 | 5.608 | 5.168 | 9.345 | 5.388 | 0.598 | -0.7413 | 3.53E-12 | 1.048E-10 | no |
| SERTAD1 | 52.608 | 43.422 | 31.996 | 28.757 | 48.015 | 30.377 | 0.598 | -0.741 | 1.901E-13 | 6.562E-12 | no |
| HSPB1 | 587.017 | 441.266 | 277.751 | 215.341 | 514.142 | 246.546 | 0.598 | -0.7425 | 0.00002447 | 0.0002273 | no |
| MRPS34 | 40.406 | 27.546 | 18.709 | 18.372 | 33.976 | 18.541 | 0.599 | -0.7394 | 6.377E-08 | 0.000001027 | no |
| FGFRL1 | 23.912 | 21.463 | 13.492 | 13.074 | 22.688 | 13.283 | 0.599 | -0.7399 | 4.467E-17 | 2.223E-15 | no |
| CTPS1 | 69.678 | 64.826 | 38.834 | 36.693 | 67.252 | 37.764 | 0.599 | -0.7403 | 3.477E-17 | 1.761E-15 | no |
| TMEM132D | 56.035 | 48.97 | 31.243 | 30.996 | 52.502 | 31.119 | 0.601 | -0.7348 | 8.894E-22 | 6.085E-20 | no |
| TLE3 | 6.348 | 5.032 | 3.244 | 3.049 | 5.69 | 3.147 | 0.602 | -0.7333 | 2.445E-07 | 0.000003599 | no |
| PLIN2 | 10.032 | 8.965 | 5.491 | 5.507 | 9.498 | 5.499 | 0.602 | -0.7326 | 2.869E-11 | 7.582E-10 | no |
| ABCA1 | 12.789 | 12.956 | 7.611 | 7.946 | 12.872 | 7.778 | 0.602 | -0.7317 | 4.107E-35 | 5.365E-33 | no |
| RDH13 | 11.04 | 9.062 | 6.106 | 5.278 | 10.051 | 5.692 | 0.603 | -0.7301 | 8.761E-09 | 1.659E-07 | no |
| CCDC86 | 9.939 | 9.812 | 4.924 | 6.147 | 9.875 | 5.536 | 0.603 | -0.7294 | 8.506E-08 | 0.000001343 | no |
| EREG | 4.733 | 4.546 | 2.54 | 2.639 | 4.639 | 2.59 | 0.604 | -0.7271 | 1.751E-07 | 0.000002641 | no |
| PPIF | 39.438 | 33.348 | 21.024 | 21.49 | 36.393 | 21.257 | 0.606 | -0.7215 | 1.349E-12 | 4.145E-11 | no |
| MINDY3 | 9.229 | 8.916 | 5.295 | 5.198 | 9.072 | 5.247 | 0.607 | -0.7198 | 2.871E-09 | 5.871E-08 | no |
| PUS1 | 23.953 | 18.221 | 12.407 | 11.195 | 21.087 | 11.801 | 0.608 | -0.7178 | 8.133E-08 | 0.000001289 | no |
| LPAR5 | 12.491 | 11.534 | 7.054 | 7.327 | 12.012 | 7.191 | 0.612 | -0.7092 | 2.242E-13 | 7.694E-12 | no |
| ABCG1 | 26.042 | 22.387 | 14.576 | 27.957 | 24.215 | 21.267 | 0.612 | -0.7094 | 3.961E-31 | 4.295E-29 | no |
| TNFRSF12A | 159.112 | 140.505 | 90.848 | 91.798 | 149.808 | 91.323 | 0.613 | -0.7049 | 3.984E-38 | 6.026E-36 | no |
| TRA2B | 132.761 | 122.917 | 78.782 | 78.504 | 127.839 | 78.643 | 0.615 | -0.7012 | 2.907E-49 | 5.861E-47 | no |
| THBS1 | 13.808 | 12.43 | 8.119 | 7.936 | 13.119 | 8.027 | 0.617 | -0.6976 | 1.866E-20 | 1.185E-18 | no |
| LIN7A | 19.086 | 17.073 | 10.952 | 10.615 | 18.079 | 10.784 | 0.617 | -0.6971 | 5.004E-24 | 3.835E-22 | no |
| WDR43 | 81.408 | 79.651 | 49.6 | 50.617 | 80.529 | 50.108 | 0.62 | -0.6892 | 2.164E-21 | 1.463E-19 | no |
| SLC23A2 | 25.054 | 23.896 | 14.967 | 15.693 | 24.475 | 15.33 | 0.623 | -0.6818 | 2.46E-35 | 3.288E-33 | no |
| PPRC1 | 22.77 | 19.458 | 12.632 | 13.004 | 21.114 | 12.818 | 0.624 | -0.6806 | 8.45E-21 | 5.487E-19 | no |
| FRMD8 | 29.633 | 25.843 | 16.706 | 17.592 | 27.738 | 17.149 | 0.624 | -0.6801 | 9.155E-19 | 5.288E-17 | no |
| RRP8 | 15.002 | 15.564 | 8.881 | 9.835 | 15.283 | 9.358 | 0.626 | -0.6763 | 1.659E-14 | 6.486E-13 | no |
| RGS3 | 50.839 | 45.787 | 28.879 | 27.148 | 48.313 | 28.014 | 0.626 | -0.6762 | 6.032E-16 | 2.751E-14 | no |
| GNL3 | 51.58 | 46.936 | 28.879 | 30.966 | 49.258 | 29.922 | 0.626 | -0.6749 | 1.125E-19 | 6.912E-18 | no |
| FTSJ3 | 58.792 | 53.944 | 35.317 | 35.234 | 56.368 | 35.276 | 0.626 | -0.6748 | 1.792E-31 | 1.961E-29 | no |
| SLC37A1 | 18.335 | 16.537 | 10.424 | 10.985 | 17.436 | 10.704 | 0.627 | -0.6732 | 1.111E-12 | 3.46E-11 | no |
| GRWD1 | 58.278 | 50.615 | 32.943 | 34.494 | 54.447 | 33.718 | 0.628 | -0.6703 | 1.728E-18 | 9.643E-17 | no |
| TGS1 | 12.635 | 11.184 | 7.435 | 7.267 | 11.909 | 7.351 | 0.629 | -0.6684 | 1.835E-12 | 5.578E-11 | no |
| RPF2 | 10.608 | 10.795 | 6.77 | 6.757 | 10.701 | 6.763 | 0.632 | -0.663 | 9.571E-09 | 1.785E-07 | no |
| ADAMTSL3 | 3.653 | 3.592 | 2.208 | 2.259 | 3.623 | 2.234 | 0.632 | -0.6621 | 3.587E-10 | 8.295E-09 | no |
| DAPK3 | 93.384 | 82.269 | 46.64 | 55.175 | 87.827 | 50.907 | 0.633 | -0.6602 | 1.46E-19 | 8.785E-18 | no |
| GABPB1 | 19.529 | 16.625 | 11.587 | 11.215 | 18.077 | 11.401 | 0.634 | -0.657 | 1.385E-11 | 3.808E-10 | no |
| GJA1 | 138.05 | 143.026 | 86.5 | 89.599 | 140.538 | 88.049 | 0.635 | -0.6545 | 1.695E-52 | 3.608E-50 | no |
| SAMD4A | 19.436 | 19.253 | 12.153 | 12.254 | 19.345 | 12.204 | 0.636 | -0.6532 | 1.61E-24 | 1.268E-22 | no |
| PTGES | 58.916 | 49.671 | 31.507 | 31.705 | 54.293 | 31.606 | 0.637 | -0.6515 | 3.183E-17 | 1.619E-15 | no |
| NDEL1 | 44.48 | 38.536 | 24.873 | 27.178 | 41.508 | 26.026 | 0.637 | -0.6505 | 9.097E-17 | 4.339E-15 | no |
| GPN2 | 7.058 | 5.315 | 3.791 | 3.548 | 6.187 | 3.67 | 0.638 | -0.6479 | 0.000003703 | 0.00004262 | no |
| SH3BP1 | 83.898 | 76.516 | 49.62 | 52.266 | 80.207 | 50.943 | 0.639 | -0.647 | 4.715E-25 | 3.871E-23 | no |
| EXOG | 6.492 | 5.538 | 3.81 | 3.598 | 6.015 | 3.704 | 0.639 | -0.6459 | 5.32E-08 | 8.698E-07 | no |
| CLCF1 | 9.353 | 10.24 | 5.715 | 5.428 | 9.796 | 5.572 | 0.639 | -0.6464 | 0.00000523 | 0.00005764 | no |
| IPO13 | 26.165 | 23.964 | 15.778 | 16.103 | 25.064 | 15.941 | 0.64 | -0.644 | 1.252E-18 | 7.126E-17 | no |
| TAF4B | 1.729 | 1.334 | 0.684 | 0.89 | 1.532 | 0.787 | 0.641 | -0.6426 | 0.0005126 | 0.003336 | no |
| GCOM1 | 2.799 | 2.813 | 1.182 | 1.339 | 2.806 | 1.26 | 0.643 | -0.6372 | 0.0009819 | 0.005818 | no |
| F3 | 45.458 | 42.108 | 26.71 | 28.877 | 43.783 | 27.794 | 0.644 | -0.6352 | 1.005E-15 | 4.51E-14 | no |
| TRA2A | 26.958 | 24.879 | 16.804 | 15.383 | 25.919 | 16.093 | 0.645 | -0.6317 | 1.167E-10 | 2.872E-09 | no |
| FERMT2 | 76.294 | 72.856 | 48.291 | 48.398 | 74.575 | 48.344 | 0.646 | -0.6313 | 9.284E-40 | 1.462E-37 | no |
| ALAS1 | 37.144 | 34.224 | 21.669 | 23.989 | 35.684 | 22.829 | 0.647 | -0.6285 | 8.234E-15 | 3.309E-13 | no |
| TRAK2 | 8.766 | 9.13 | 5.735 | 5.757 | 8.948 | 5.746 | 0.649 | -0.6236 | 4.874E-13 | 1.605E-11 | no |
| HIP1 | 28.614 | 28.013 | 18.484 | 18.581 | 28.314 | 18.532 | 0.651 | -0.6202 | 2.341E-33 | 2.832E-31 | no |
| SNORA71 | 21.144 | 16.43 | 11.352 | 10.935 | 18.787 | 11.143 | 0.653 | -0.6156 | 0.00002981 | 0.0002719 | no |
| PLEKHA6 | 6.287 | 5.947 | 3.752 | 4.168 | 6.117 | 3.96 | 0.653 | -0.6138 | 2.319E-10 | 5.52E-09 | no |
| NIPAL4 | 7.213 | 5.743 | 3.41 | 3.648 | 6.478 | 3.529 | 0.653 | -0.6148 | 3.577E-07 | 0.000005018 | no |
| HERPUD1 | 27.225 | 28.714 | 16.335 | 19.311 | 27.97 | 17.823 | 0.653 | -0.6155 | 1.639E-09 | 3.502E-08 | no |
| RRP1 | 12.347 | 11.7 | 8.089 | 7.477 | 12.023 | 7.783 | 0.654 | -0.6121 | 5.502E-13 | 1.802E-11 | no |
| IPPK | 9.898 | 8.838 | 5.754 | 6.127 | 9.368 | 5.941 | 0.654 | -0.6125 | 3.042E-08 | 5.155E-07 | no |
| CHST9 | 17.996 | 17.199 | 10.62 | 11.265 | 17.598 | 10.942 | 0.656 | -0.6084 | 1.146E-17 | 6.097E-16 | no |
| CLK1 | 20.383 | 18.27 | 12.271 | 12.184 | 19.326 | 12.227 | 0.657 | -0.6069 | 1.267E-07 | 0.000001944 | no |
| CDC42EP2 | 21.247 | 19.876 | 13.345 | 11.455 | 20.562 | 12.4 | 0.657 | -0.6064 | 0.00001781 | 0.00017 | no |
| TWISTNB | 4.98 | 4.283 | 3.009 | 2.629 | 4.632 | 2.819 | 0.658 | -0.6037 | 0.00003881 | 0.0003447 | no |
| SUPT6H | 24.715 | 22.757 | 15.368 | 15.983 | 23.736 | 15.675 | 0.658 | -0.6031 | 9.72E-25 | 7.813E-23 | no |
| MIER2 | 4.692 | 4.867 | 3.048 | 2.839 | 4.78 | 2.944 | 0.658 | -0.6049 | 0.0001323 | 0.001018 | no |
| DUSP5 | 36.578 | 33.678 | 22.636 | 22.839 | 35.128 | 22.737 | 0.658 | -0.6049 | 8.792E-13 | 2.807E-11 | no |
| TNFRSF21 | 37.689 | 35.645 | 24.727 | 23.869 | 36.667 | 24.298 | 0.659 | -0.6014 | 1.1E-32 | 1.29E-30 | no |
| OTUD6B | 19.488 | 17.91 | 11.616 | 12.684 | 18.699 | 12.15 | 0.659 | -0.6025 | 8.593E-12 | 2.439E-10 | no |
| UAP1 | 82.385 | 77.003 | 53.665 | 51.396 | 79.694 | 52.531 | 0.661 | -0.5969 | 2.01E-25 | 1.674E-23 | no |
| ST3GAL1 | 31.464 | 27.858 | 19.784 | 18.571 | 29.661 | 19.178 | 0.661 | -0.5983 | 5.076E-13 | 1.667E-11 | no |
| CD3EAP | 3.056 | 2.803 | 1.934 | 1.659 | 2.929 | 1.796 | 0.661 | -0.5982 | 0.00005179 | 0.0004452 | no |
| TOE1 | 13.705 | 12.654 | 8.304 | 7.766 | 13.18 | 8.035 | 0.662 | -0.5948 | 0.000007211 | 0.00007646 | no |
| STRIP1 | 10.721 | 10.425 | 6.526 | 7.367 | 10.573 | 6.947 | 0.662 | -0.5941 | 4.718E-12 | 1.374E-10 | no |
| SPRY4 | 18.911 | 17.199 | 10.883 | 11.765 | 18.055 | 11.324 | 0.662 | -0.5948 | 5.642E-13 | 1.842E-11 | no |
| SEMA3A | 89.619 | 88.43 | 59.517 | 59.763 | 89.025 | 59.64 | 0.662 | -0.5946 | 9.892E-79 | 4.373E-76 | no |
| GPRC5A | 208.098 | 185.699 | 130.327 | 133.239 | 196.899 | 131.783 | 0.662 | -0.596 | 7.495E-66 | 2.154E-63 | no |
| ZNF697 | 5.813 | 5.159 | 3.537 | 2.959 | 5.486 | 3.248 | 0.663 | -0.5919 | 0.0002098 | 0.001537 | no |
| PTCD3 | 28.48 | 26.699 | 17.566 | 18.202 | 27.59 | 17.884 | 0.663 | -0.594 | 3.706E-14 | 1.392E-12 | no |
| NOV | 8.808 | 5.422 | 4.396 | 4.138 | 7.115 | 4.267 | 0.663 | -0.5933 | 0.0001564 | 0.001181 | no |
| KIAA0040 | 19.519 | 17.268 | 12.017 | 12.184 | 18.393 | 12.101 | 0.663 | -0.5919 | 2.779E-13 | 9.423E-12 | no |
| BRIX1 | 62.424 | 54.168 | 36.509 | 38.912 | 58.296 | 37.71 | 0.663 | -0.5936 | 3.08E-11 | 8.12E-10 | no |
| ARF6 | 332.731 | 312.879 | 231.491 | 234.442 | 322.805 | 232.966 | 0.663 | -0.5935 | 2.397E-40 | 3.993E-38 | no |
| NOC3L | 13.818 | 14.026 | 8.754 | 9.596 | 13.922 | 9.175 | 0.664 | -0.5908 | 4.906E-12 | 1.424E-10 | no |
| FAM207A | 24.519 | 21.064 | 14.625 | 14.863 | 22.791 | 14.744 | 0.664 | -0.5908 | 5.803E-11 | 1.463E-09 | no |
| URB2 | 31.135 | 27.459 | 18.846 | 20.241 | 29.297 | 19.544 | 0.666 | -0.5867 | 2.62E-20 | 1.654E-18 | no |
| RRP7A | 68.742 | 60.835 | 42.732 | 42.301 | 64.788 | 42.517 | 0.666 | -0.5855 | 7.96E-14 | 2.895E-12 | no |
| PAK1IP1 | 18.397 | 16.791 | 11.968 | 11.245 | 17.594 | 11.607 | 0.666 | -0.5854 | 1.307E-12 | 4.05E-11 | no |
| YIPF4 | 7.881 | 8.449 | 5.647 | 4.858 | 8.165 | 5.252 | 0.667 | -0.5852 | 9.115E-07 | 0.00001185 | no |
| VCL | 92.201 | 89.413 | 60.943 | 61.572 | 90.807 | 61.258 | 0.667 | -0.5833 | 6.611E-68 | 2.054E-65 | no |
| PRPF39 | 13.53 | 12.975 | 8.685 | 8.486 | 13.252 | 8.585 | 0.668 | -0.5813 | 9.518E-09 | 1.779E-07 | no |
| IER2 | 55.448 | 47.267 | 33.148 | 29.147 | 51.358 | 31.148 | 0.668 | -0.5815 | 0.00002652 | 0.0002448 | no |
| ARHGAP17 | 38.718 | 36.462 | 23.613 | 26.398 | 37.59 | 25.005 | 0.668 | -0.5815 | 1.36E-18 | 7.699E-17 | no |
| TUBB6 | 227.967 | 202.957 | 141.103 | 146.103 | 215.462 | 143.603 | 0.669 | -0.5806 | 7.759E-42 | 1.331E-39 | no |
| ST6GAL2 | 6.297 | 5.782 | 3.898 | 4.018 | 6.04 | 3.958 | 0.669 | -0.5793 | 1.055E-08 | 1.931E-07 | no |
| OSGIN1 | 18.181 | 14.961 | 10.629 | 12.134 | 16.571 | 11.381 | 0.669 | -0.5789 | 1.815E-07 | 0.000002728 | no |
| NOP56 | 173.907 | 154.035 | 113.035 | 118.705 | 163.971 | 115.87 | 0.67 | -0.5768 | 7.425E-32 | 8.367E-30 | no |
| MICAL2 | 20.115 | 19.097 | 13.336 | 13.684 | 19.606 | 13.51 | 0.67 | -0.578 | 2.458E-21 | 1.647E-19 | no |
| PHLDB2 | 23.552 | 25.113 | 19.295 | 16.213 | 24.332 | 17.754 | 0.671 | -0.5751 | 4.799E-33 | 5.746E-31 | no |
| ELP6 | 7.058 | 6.901 | 4.504 | 4.368 | 6.979 | 4.436 | 0.671 | -0.575 | 0.0000462 | 0.000401 | no |
| SERPINB2 | 2.624 | 1.606 | 0.977 | 0.82 | 2.115 | 0.898 | 0.672 | -0.5724 | 0.003203 | 0.01581 | no |
| ITIH4 | 18.418 | 18.397 | 12.339 | 11.955 | 18.407 | 12.147 | 0.672 | -0.5739 | 1.663E-10 | 4.008E-09 | no |
| EFHD2 | 383.323 | 355.931 | 247.435 | 255.133 | 369.627 | 251.284 | 0.672 | -0.5741 | 7.671E-38 | 1.116E-35 | no |
| MAST4 | 8.2 | 7.836 | 5.276 | 5.358 | 8.018 | 5.317 | 0.673 | -0.5711 | 2.317E-14 | 8.818E-13 | no |
| FST | 272.2 | 234.63 | 170.656 | 167.653 | 253.415 | 169.154 | 0.673 | -0.5709 | 5.317E-15 | 2.19E-13 | no |
| MKNK2 | 61.632 | 56.085 | 39.919 | 40.241 | 58.858 | 40.08 | 0.675 | -0.5679 | 7.371E-27 | 6.38E-25 | no |
| HIF1AN | 8.643 | 8.303 | 5.705 | 5.657 | 8.473 | 5.681 | 0.675 | -0.5677 | 5.71E-12 | 1.649E-10 | no |
| AZIN1 | 62.26 | 61.127 | 41.628 | 42.171 | 61.694 | 41.9 | 0.675 | -0.5679 | 1.505E-38 | 2.338E-36 | no |
| SERPINB9 | 153.062 | 146.248 | 100.637 | 101.983 | 149.655 | 101.31 | 0.676 | -0.5659 | 1.425E-37 | 2.048E-35 | no |
| LLPH | 90.925 | 79.3 | 54.974 | 57.104 | 85.112 | 56.039 | 0.676 | -0.5642 | 6.452E-11 | 1.619E-09 | no |
| DLGAP4 | 38.152 | 38.399 | 32.455 | 29.496 | 38.276 | 30.975 | 0.677 | -0.562 | 1.447E-17 | 7.629E-16 | no |
| ARL4D | 7.161 | 8.478 | 3.664 | 3.139 | 7.819 | 3.401 | 0.677 | -0.562 | 0.002336 | 0.01204 | no |
| URB1 | 7.244 | 6.619 | 4.035 | 5.248 | 6.931 | 4.642 | 0.68 | -0.5566 | 0.000006362 | 0.0000685 | no |
| ZFP69 | 9.271 | 8.955 | 6.194 | 6.297 | 9.113 | 6.245 | 0.683 | -0.5494 | 4.007E-07 | 0.000005562 | no |
| PTBP3 | 57.465 | 53.973 | 39.049 | 37.783 | 55.719 | 38.416 | 0.683 | -0.5499 | 2.688E-40 | 4.413E-38 | no |
| NOB1 | 84.659 | 75.085 | 54.358 | 54.975 | 79.872 | 54.666 | 0.683 | -0.5499 | 3.557E-24 | 2.781E-22 | no |
| DDX51 | 7.079 | 6.054 | 4.24 | 4.358 | 6.566 | 4.299 | 0.683 | -0.551 | 7.166E-07 | 0.000009555 | no |
| CSPG4 | 20.938 | 19.146 | 12.026 | 13.794 | 20.042 | 12.91 | 0.683 | -0.5492 | 6.518E-29 | 6.243E-27 | no |
| TMEM151A | 7.13 | 5.889 | 3.654 | 3.858 | 6.51 | 3.756 | 0.684 | -0.5473 | 0.002331 | 0.01203 | no |
| SEC24A | 8.231 | 7.66 | 5.305 | 5.418 | 7.946 | 5.361 | 0.684 | -0.5488 | 1.084E-09 | 2.393E-08 | no |
| NFKBIE | 17.286 | 15.613 | 10.62 | 11.495 | 16.45 | 11.057 | 0.685 | -0.5464 | 2.472E-08 | 4.273E-07 | no |
| NOL6 | 4.887 | 4.838 | 3.175 | 3.378 | 4.862 | 3.276 | 0.687 | -0.542 | 0.000000384 | 0.00000535 | no |
| NEPRO | 4.61 | 4.565 | 2.794 | 3.049 | 4.588 | 2.921 | 0.687 | -0.5408 | 0.000388 | 0.002602 | no |
| LARP4 | 22.729 | 21.21 | 15.553 | 14.783 | 21.97 | 15.168 | 0.688 | -0.5388 | 4.532E-19 | 2.658E-17 | no |
| ERRFI1 | 36.321 | 29.902 | 22.929 | 21.5 | 33.111 | 22.215 | 0.688 | -0.5389 | 0.000001108 | 0.00001424 | no |
| DPH1 | 18.5 | 16.791 | 11.753 | 12.504 | 17.645 | 12.128 | 0.688 | -0.5399 | 3.723E-09 | 7.43E-08 | no |
| CRY1 | 4.085 | 3.816 | 2.833 | 2.439 | 3.95 | 2.636 | 0.688 | -0.5403 | 0.000004789 | 0.00005323 | no |
| ZNF408 | 5.937 | 5.928 | 3.693 | 3.668 | 5.933 | 3.681 | 0.689 | -0.537 | 0.00006313 | 0.0005324 | no |
| SYNE3 | 21.504 | 20.762 | 14.098 | 14.863 | 21.133 | 14.48 | 0.689 | -0.5377 | 1.347E-13 | 4.707E-12 | no |
| SRRM1 | 56.158 | 53.886 | 38.17 | 38.182 | 55.022 | 38.176 | 0.689 | -0.5373 | 9.4E-27 | 8.063E-25 | no |
| SRF | 15.526 | 15.116 | 10.532 | 10.435 | 15.321 | 10.483 | 0.689 | -0.5383 | 1.189E-09 | 2.608E-08 | no |
| NFKB2 | 49.244 | 46.186 | 33.275 | 32.585 | 47.715 | 32.93 | 0.689 | -0.5373 | 1.603E-20 | 1.029E-18 | no |
| SMG5 | 23.408 | 20.713 | 15.241 | 15.163 | 22.061 | 15.202 | 0.69 | -0.5355 | 1.761E-13 | 6.134E-12 | no |
| FHL2 | 170.913 | 148.954 | 110.68 | 106.881 | 159.934 | 108.781 | 0.69 | -0.5357 | 5.994E-23 | 4.388E-21 | no |
| PLAU | 9.332 | 8.254 | 5.412 | 6.127 | 8.793 | 5.769 | 0.691 | -0.5329 | 0.00008665 | 0.0007034 | no |
| NOM1 | 7.058 | 6.015 | 4.543 | 4.238 | 6.537 | 4.391 | 0.692 | -0.5318 | 0.000004533 | 0.00005068 | no |
| EFNB2 | 24.581 | 22.933 | 15.807 | 17.072 | 23.757 | 16.439 | 0.692 | -0.5321 | 6.014E-15 | 2.46E-13 | no |
| RBM34 | 21.021 | 19.759 | 14.039 | 14.044 | 20.39 | 14.041 | 0.693 | -0.5284 | 3.308E-10 | 7.744E-09 | no |
| AKT2 | 34.273 | 32.452 | 22.871 | 23.579 | 33.362 | 23.225 | 0.693 | -0.5288 | 4.642E-17 | 2.29E-15 | no |
| QTRT2 | 5.587 | 4.76 | 3.077 | 3.708 | 5.173 | 3.393 | 0.694 | -0.5269 | 0.0002139 | 0.001565 | no |
| PLK3 | 2.963 | 2.151 | 1.407 | 1.529 | 2.557 | 1.468 | 0.694 | -0.5262 | 0.004498 | 0.02081 | no |
| CRY2 | 3.21 | 3.008 | 2.032 | 1.939 | 3.109 | 1.986 | 0.694 | -0.5268 | 0.0007446 | 0.004569 | no |
| C15orf48 | 1.729 | 1.84 | 0.85 | 0.74 | 1.784 | 0.795 | 0.694 | -0.5268 | 0.006601 | 0.02884 | no |
| UTP6 | 29.767 | 28.237 | 19.608 | 20.67 | 29.002 | 20.139 | 0.695 | -0.5258 | 1.321E-14 | 5.218E-13 | no |
| LRRC8C | 14.199 | 12.839 | 9.135 | 9.646 | 13.519 | 9.39 | 0.696 | -0.5235 | 2.904E-13 | 9.816E-12 | no |
| FAAP100 | 8.797 | 8.118 | 5.373 | 5.957 | 8.457 | 5.665 | 0.696 | -0.522 | 0.00005844 | 0.0004961 | no |
| WDR74 | 17.45 | 15.788 | 11.88 | 10.405 | 16.619 | 11.143 | 0.697 | -0.52 | 0.000007146 | 0.00007585 | no |
| POLR1C | 31.999 | 28.422 | 21.093 | 19.831 | 30.21 | 20.462 | 0.698 | -0.5194 | 0.000001257 | 0.00001597 | no |
| LUC7L3 | 41.434 | 39.09 | 28.146 | 28.677 | 40.262 | 28.412 | 0.698 | -0.519 | 2.312E-12 | 6.955E-11 | no |
| ZC3H18 | 39.922 | 35.051 | 26.251 | 24.129 | 37.486 | 25.19 | 0.699 | -0.5167 | 9.334E-14 | 3.342E-12 | no |
| RPUSD2 | 4.27 | 3.485 | 2.521 | 2.589 | 3.877 | 2.555 | 0.7 | -0.5141 | 0.0004594 | 0.003024 | no |
| DDX27 | 58.669 | 51.481 | 38.522 | 38.592 | 55.075 | 38.557 | 0.7 | -0.5147 | 2.916E-17 | 1.49E-15 | no |
| UGDH | 83.208 | 77.237 | 56.234 | 56.744 | 80.222 | 56.489 | 0.701 | -0.5131 | 2.109E-27 | 1.924E-25 | no |
| ZNHIT6 | 14.384 | 15.185 | 10.795 | 12.674 | 14.785 | 11.735 | 0.702 | -0.5104 | 2.447E-09 | 5.068E-08 | no |
| TEAD1 | 45.746 | 46.576 | 34.057 | 32.725 | 46.161 | 33.391 | 0.702 | -0.5106 | 5.003E-29 | 4.915E-27 | no |
| DHX33 | 25.435 | 21.56 | 16.306 | 16.342 | 23.497 | 16.324 | 0.702 | -0.5113 | 2.131E-09 | 4.462E-08 | no |
| TMTC2 | 7.84 | 8.235 | 5.432 | 5.847 | 8.037 | 5.639 | 0.703 | -0.5075 | 5.291E-09 | 1.036E-07 | no |
| RBPMS | 14.611 | 13.452 | 9.418 | 9.266 | 14.032 | 9.342 | 0.703 | -0.5078 | 0.000000742 | 0.000009882 | no |
| SF1 | 83.558 | 78.998 | 56.459 | 57.763 | 81.278 | 57.111 | 0.704 | -0.5066 | 5.438E-26 | 4.596E-24 | no |
| SERPINB8 | 8.787 | 8.497 | 5.881 | 5.388 | 8.642 | 5.635 | 0.704 | -0.5058 | 6.046E-08 | 9.801E-07 | no |
| RRS1 | 61.591 | 57.205 | 40.984 | 37.673 | 59.398 | 39.329 | 0.704 | -0.5054 | 1.657E-08 | 2.934E-07 | no |
| EPHA4 | 4.507 | 4.049 | 2.872 | 3.009 | 4.278 | 2.941 | 0.704 | -0.5056 | 0.00001601 | 0.0001547 | no |
| NOL9 | 22.132 | 21.093 | 15.631 | 14.893 | 21.613 | 15.262 | 0.705 | -0.5045 | 1.307E-13 | 4.581E-12 | no |
| ZFR | 43.173 | 43.033 | 30.423 | 31.006 | 43.103 | 30.715 | 0.707 | -0.5009 | 9.263E-28 | 8.656E-26 | no |
| ZBTB2 | 8.571 | 8.682 | 6.077 | 5.847 | 8.627 | 5.962 | 0.707 | -0.4994 | 0.00002633 | 0.0002433 | no |
| UBASH3B | 3.344 | 3.037 | 2.286 | 2.059 | 3.191 | 2.173 | 0.707 | -0.5005 | 0.00009815 | 0.000785 | no |
| FOSL1 | 59.06 | 51.54 | 39.313 | 38.752 | 55.3 | 39.032 | 0.707 | -0.5002 | 4.406E-15 | 1.835E-13 | no |
| RPIA | 6.091 | 5.704 | 3.771 | 4.028 | 5.897 | 3.899 | 0.708 | -0.4979 | 0.0009457 | 0.005629 | no |
| NOLC1 | 90.298 | 81.101 | 60.816 | 62.101 | 85.7 | 61.459 | 0.708 | -0.4989 | 9.787E-23 | 7.075E-21 | no |
| LFNG | 5.052 | 3.981 | 2.755 | 3.099 | 4.516 | 2.927 | 0.708 | -0.4977 | 0.001979 | 0.01047 | no |
| TRMT6 | 17.224 | 15.438 | 11.938 | 10.995 | 16.331 | 11.466 | 0.709 | -0.4956 | 1.923E-08 | 0.000000337 | no |
| RAI14 | 19.971 | 19.526 | 14.166 | 13.824 | 19.748 | 13.995 | 0.709 | -0.4965 | 9.314E-13 | 2.949E-11 | no |
| DUSP7 | 20.167 | 15.525 | 12.105 | 12.854 | 17.846 | 12.479 | 0.709 | -0.4961 | 0.000001192 | 0.00001521 | no |
| ZC3HC1 | 22.893 | 22.095 | 17.097 | 13.984 | 22.494 | 15.541 | 0.71 | -0.494 | 0.000007267 | 0.0000769 | no |
| TMEM138 | 24.601 | 19.925 | 15.211 | 15.593 | 22.263 | 15.402 | 0.71 | -0.4946 | 0.000006157 | 0.00006645 | no |
| SRSF5 | 197.542 | 178.097 | 112.771 | 111.959 | 187.82 | 112.365 | 0.71 | -0.4945 | 1.656E-18 | 9.284E-17 | no |
| OSBPL6 | 2.953 | 3.008 | 1.886 | 2.099 | 2.981 | 1.993 | 0.71 | -0.4946 | 0.001627 | 0.008869 | no |
| NOCT | 13.335 | 14.026 | 9.447 | 9.246 | 13.681 | 9.346 | 0.71 | -0.4935 | 0.00001909 | 0.0001806 | no |
| KCTD5 | 15.495 | 13.627 | 10.366 | 9.706 | 14.561 | 10.036 | 0.71 | -0.4935 | 0.00001841 | 0.0001755 | no |
| TMEM39A | 10.238 | 9.597 | 6.858 | 6.877 | 9.918 | 6.867 | 0.711 | -0.4913 | 0.00001456 | 0.000142 | no |
| SH3BP5 | 28.696 | 29.182 | 20.067 | 20.96 | 28.939 | 20.514 | 0.711 | -0.4915 | 1.601E-10 | 3.874E-09 | no |
| NUP153 | 12.378 | 11.641 | 8.773 | 8.576 | 12.009 | 8.675 | 0.711 | -0.4928 | 1.391E-18 | 7.837E-17 | no |
| ERI1 | 19.241 | 17.881 | 13.179 | 13.054 | 18.561 | 13.117 | 0.711 | -0.4914 | 3.608E-09 | 7.238E-08 | no |
| CDCP1 | 22.019 | 20.48 | 15.368 | 14.953 | 21.249 | 15.16 | 0.711 | -0.4915 | 2.825E-14 | 1.068E-12 | no |
| SSTR2 | 6.143 | 5.762 | 3.351 | 3.398 | 5.952 | 3.375 | 0.712 | -0.4893 | 0.009909 | 0.04047 | no |
| RGP1 | 9.785 | 10.697 | 7.142 | 6.857 | 10.241 | 7 | 0.712 | -0.491 | 0.0009163 | 0.005474 | no |
| NDUFAF4 | 11.071 | 9.938 | 7.278 | 7.147 | 10.505 | 7.213 | 0.712 | -0.4892 | 0.000103 | 0.0008201 | no |
| DSN1 | 25.332 | 21.249 | 16.745 | 15.613 | 23.291 | 16.179 | 0.712 | -0.4899 | 0.000005056 | 0.00005588 | no |
| OSER1 | 17.636 | 16.518 | 12.007 | 12.414 | 17.077 | 12.21 | 0.713 | -0.4872 | 1.033E-12 | 3.234E-11 | no |
| PUM3 | 36.496 | 32.141 | 23.672 | 25.348 | 34.319 | 24.51 | 0.714 | -0.4863 | 4.035E-09 | 8.01E-08 | no |
| MYL9 | 65.974 | 58.441 | 46.836 | 48.298 | 62.208 | 47.567 | 0.714 | -0.4857 | 5.615E-29 | 5.469E-27 | no |
| DNTTIP2 | 18.057 | 16.314 | 12.153 | 12.344 | 17.185 | 12.248 | 0.714 | -0.4865 | 4.824E-11 | 1.235E-09 | no |
| BRD2 | 77.56 | 71.611 | 53.137 | 53.375 | 74.585 | 53.256 | 0.714 | -0.4867 | 1.272E-30 | 1.367E-28 | no |
| WFS1 | 61.416 | 54.148 | 41.619 | 41.201 | 57.782 | 41.41 | 0.715 | -0.4846 | 3.003E-18 | 1.643E-16 | no |
| CHFR | 47.917 | 42.186 | 32.113 | 32.075 | 45.052 | 32.094 | 0.715 | -0.4845 | 7.081E-09 | 1.356E-07 | no |
| TRIM38 | 12.512 | 11.749 | 8.607 | 8.496 | 12.131 | 8.552 | 0.716 | -0.4821 | 0.000003528 | 0.00004104 | no |
| PLA2G7 | 2.284 | 3.28 | 1.426 | 1.719 | 2.782 | 1.573 | 0.717 | -0.4795 | 0.01269 | 0.04925 | no |
| SRSF4 | 43.163 | 39.266 | 30.051 | 28.877 | 41.215 | 29.464 | 0.718 | -0.4777 | 3.656E-11 | 9.594E-10 | no |
| PITPNB | 46.301 | 43.023 | 32.777 | 31.366 | 44.662 | 32.072 | 0.718 | -0.4784 | 1.924E-14 | 7.422E-13 | no |
| FAM111B | 19.014 | 16.654 | 12.788 | 12.584 | 17.834 | 12.686 | 0.718 | -0.4785 | 4.77E-08 | 7.843E-07 | no |
| TMEM185B | 27.75 | 24.217 | 19.598 | 17.142 | 25.983 | 18.37 | 0.719 | -0.4754 | 0.00002187 | 0.0002049 | no |
| PINX1 | 24.807 | 22.144 | 15.407 | 16.932 | 23.475 | 16.169 | 0.719 | -0.4765 | 0.0001176 | 0.0009229 | no |
| FLNC | 36.619 | 34.243 | 25.391 | 26.108 | 35.431 | 25.749 | 0.719 | -0.4753 | 2.965E-30 | 3.155E-28 | no |
| FGL2 | 0.854 | 0.886 | 0.459 | 0.41 | 0.87 | 0.434 | 0.719 | -0.4762 | 0.01415 | 0.05385 | no |
| FGFR2 | 1.821 | 1.694 | 1.124 | 1.06 | 1.757 | 1.092 | 0.719 | -0.4752 | 0.007189 | 0.03092 | no |
| RBM28 | 16.71 | 15.759 | 11.938 | 11.365 | 16.235 | 11.652 | 0.72 | -0.4742 | 7.976E-09 | 0.000000152 | no |
| TGIF2 | 2.274 | 1.752 | 1.29 | 1.149 | 2.013 | 1.22 | 0.721 | -0.4714 | 0.0114 | 0.04536 | no |
| NOL10 | 27.133 | 25.278 | 18.367 | 19.271 | 26.206 | 18.819 | 0.721 | -0.4711 | 1.779E-09 | 3.772E-08 | no |
| HIVEP3 | 0.473 | 0.38 | 0.254 | 0.26 | 0.426 | 0.257 | 0.721 | -0.4717 | 0.01217 | 0.04759 | no |
| FAM53C | 9.116 | 8.517 | 6.516 | 5.977 | 8.816 | 6.247 | 0.721 | -0.4713 | 0.000009543 | 0.00009811 | no |
| RGS20 | 2.305 | 1.956 | 1.319 | 1.08 | 2.131 | 1.2 | 0.722 | -0.4693 | 0.0134 | 0.0515 | no |
| PABPC4 | 56.024 | 54.051 | 39.098 | 40.481 | 55.038 | 39.79 | 0.722 | -0.4693 | 3.447E-16 | 1.604E-14 | no |
| NAP1L4 | 68.845 | 64.057 | 48.194 | 47.788 | 66.451 | 47.991 | 0.722 | -0.4708 | 6.709E-17 | 3.254E-15 | no |
| EXOC3 | 48.462 | 45.933 | 33.529 | 35.374 | 47.198 | 34.452 | 0.722 | -0.4708 | 2.33E-15 | 1.003E-13 | no |
| THRB | 3.91 | 4.351 | 2.608 | 3.139 | 4.13 | 2.873 | 0.723 | -0.4675 | 0.00018 | 0.00134 | no |
| RGS12 | 33.028 | 29.113 | 21.278 | 20.86 | 31.07 | 21.069 | 0.723 | -0.4679 | 1.453E-12 | 4.453E-11 | no |
| JUNB | 20.702 | 18.893 | 13.541 | 13.164 | 19.797 | 13.352 | 0.723 | -0.4687 | 0.001181 | 0.006777 | no |
| ABCC9 | 2.377 | 2.443 | 1.534 | 1.769 | 2.41 | 1.651 | 0.723 | -0.4672 | 0.001516 | 0.008371 | no |
| LTBP1 | 63.494 | 57.614 | 43.358 | 43.78 | 60.554 | 43.569 | 0.724 | -0.4664 | 2.26E-25 | 1.869E-23 | no |
| DOHH | 20.424 | 17.014 | 13.375 | 13.874 | 18.719 | 13.625 | 0.724 | -0.4657 | 0.0001525 | 0.001156 | no |
| ACTN1 | 199.157 | 178.311 | 139.061 | 137.217 | 188.734 | 138.139 | 0.724 | -0.4653 | 2.248E-43 | 3.975E-41 | no |
| SLC19A2 | 15.3 | 12.011 | 9.74 | 9.726 | 13.655 | 9.733 | 0.725 | -0.4643 | 0.000005776 | 0.00006281 | no |
| SECISBP2 | 6.739 | 7.135 | 4.689 | 5.058 | 6.937 | 4.873 | 0.725 | -0.464 | 0.0002963 | 0.002067 | no |
| NIP7 | 28.46 | 24.256 | 19.451 | 18.032 | 26.358 | 18.742 | 0.725 | -0.4646 | 0.000002715 | 0.00003251 | no |
| ZBTB9 | 3.498 | 2.784 | 2.022 | 1.309 | 3.141 | 1.665 | 0.726 | -0.4614 | 0.01764 | 0.06405 | no |
| SRRM2 | 78.897 | 60.855 | 42.439 | 44.939 | 69.876 | 43.689 | 0.726 | -0.4626 | 2.735E-38 | 4.191E-36 | no |
| SLC7A5 | 59.759 | 51.978 | 41.443 | 39.852 | 55.868 | 40.647 | 0.726 | -0.4624 | 7.768E-16 | 3.529E-14 | no |
| MED9 | 28.244 | 24.354 | 19.178 | 21.41 | 26.299 | 20.294 | 0.726 | -0.462 | 3.335E-07 | 0.00000472 | no |
| BHLHE40 | 14.106 | 13.033 | 9.213 | 10.285 | 13.569 | 9.749 | 0.726 | -0.4612 | 0.000003644 | 0.00004213 | no |
| VEGFA | 6.729 | 6.288 | 4.748 | 4.368 | 6.508 | 4.558 | 0.728 | -0.4584 | 0.0003596 | 0.002439 | no |
| HSPH1 | 47.793 | 44.824 | 33.901 | 33.924 | 46.308 | 33.913 | 0.728 | -0.4584 | 1.165E-19 | 7.123E-18 | no |
| GDF15 | 75.039 | 61.118 | 49.991 | 48.088 | 68.079 | 49.04 | 0.728 | -0.4589 | 2.333E-09 | 4.849E-08 | no |
| CCBE1 | 5.474 | 5.013 | 4.015 | 3.738 | 5.244 | 3.877 | 0.728 | -0.4586 | 0.000004058 | 0.00004609 | no |
| ASTE1 | 2.819 | 2.492 | 1.749 | 1.679 | 2.655 | 1.714 | 0.728 | -0.4578 | 0.009478 | 0.03903 | no |
| IL11 | 6.4 | 4.546 | 3.585 | 3.328 | 5.473 | 3.457 | 0.729 | -0.4553 | 0.0116 | 0.04598 | no |
| DDX18 | 40.364 | 37.806 | 29.26 | 27.977 | 39.085 | 28.619 | 0.729 | -0.4567 | 9.26E-17 | 4.398E-15 | no |
| CC2D1B | 9.075 | 8.536 | 6.331 | 6.537 | 8.805 | 6.434 | 0.729 | -0.4555 | 9.636E-10 | 2.155E-08 | no |
| BCAR3 | 28.182 | 23.332 | 18.484 | 18.811 | 25.757 | 18.648 | 0.729 | -0.4563 | 6.743E-08 | 0.000001081 | no |
| BAZ1A | 35.734 | 33.26 | 25.772 | 24.869 | 34.497 | 25.32 | 0.729 | -0.4552 | 1.906E-18 | 1.058E-16 | no |
| ZSWIM3 | 2.83 | 2.784 | 2.032 | 1.899 | 2.807 | 1.966 | 0.73 | -0.4531 | 0.0008245 | 0.004982 | no |
| RRN3 | 29.458 | 29.912 | 22.46 | 20.351 | 29.685 | 21.405 | 0.73 | -0.4533 | 3.864E-11 | 1.003E-09 | no |
| PNN | 52.228 | 50.829 | 37.545 | 37.703 | 51.529 | 37.624 | 0.73 | -0.4539 | 3.191E-09 | 6.468E-08 | no |
| GEMIN5 | 14.497 | 14.153 | 10.366 | 10.685 | 14.325 | 10.526 | 0.731 | -0.4516 | 1.926E-12 | 5.825E-11 | no |
| FARSA | 35.93 | 35.411 | 25.538 | 26.688 | 35.671 | 26.113 | 0.731 | -0.452 | 8.842E-13 | 2.815E-11 | no |
| YTHDF3 | 39.109 | 38.789 | 28.615 | 30.026 | 38.949 | 29.32 | 0.732 | -0.4506 | 7.576E-22 | 5.278E-20 | no |
| TGM3 | 185.678 | 159.613 | 126.321 | 125.612 | 172.645 | 125.966 | 0.732 | -0.4509 | 1.058E-09 | 2.346E-08 | no |
| NAMPT | 41.352 | 38.088 | 28.977 | 29.586 | 39.72 | 29.282 | 0.732 | -0.45 | 2.526E-18 | 1.396E-16 | no |
| GNL2 | 40.735 | 36.258 | 28.078 | 27.947 | 38.496 | 28.012 | 0.732 | -0.4505 | 1.338E-09 | 2.923E-08 | no |
| DNAJA2 | 30.363 | 30.739 | 22.46 | 22.12 | 30.551 | 22.29 | 0.732 | -0.4506 | 6.029E-09 | 0.000000117 | no |
| TXNDC9 | 20.743 | 19.205 | 14.508 | 14.853 | 19.974 | 14.68 | 0.733 | -0.4489 | 4.585E-09 | 8.993E-08 | no |
| SRRD | 9.6 | 9.208 | 6.018 | 5.967 | 9.404 | 5.992 | 0.733 | -0.4473 | 0.01157 | 0.04592 | no |
| NOP58 | 32.102 | 29.22 | 22.285 | 22.8 | 30.661 | 22.543 | 0.733 | -0.4482 | 8.188E-12 | 2.335E-10 | no |
| CFLAR | 4.826 | 5.967 | 3.498 | 3.858 | 5.396 | 3.678 | 0.733 | -0.4482 | 0.002193 | 0.01145 | no |
| ATF4 | 462.498 | 422.159 | 317.093 | 331.717 | 442.328 | 324.405 | 0.733 | -0.4474 | 1.409E-33 | 1.741E-31 | no |
| PHRF1 | 15.094 | 14.308 | 10.668 | 11.065 | 14.701 | 10.866 | 0.734 | -0.4458 | 9.284E-14 | 3.335E-12 | no |
| NSRP1 | 19.261 | 19.711 | 12.378 | 12.704 | 19.486 | 12.541 | 0.734 | -0.4463 | 0.000004629 | 0.00005165 | no |
| EIF4A1 | 432.464 | 387.215 | 300.338 | 307.219 | 409.839 | 303.779 | 0.734 | -0.4464 | 8.176E-46 | 1.516E-43 | no |
| CXorf56 | 6.266 | 3.806 | 3.117 | 3.628 | 5.036 | 3.373 | 0.734 | -0.446 | 0.006742 | 0.02935 | no |
| SPECC1 | 54.162 | 50.489 | 38.297 | 39.192 | 52.325 | 38.745 | 0.735 | -0.4436 | 4.027E-21 | 2.645E-19 | no |
| SNORD88 | 131.938 | 111.558 | 86.549 | 90.179 | 121.748 | 88.364 | 0.735 | -0.4436 | 6.269E-14 | 2.324E-12 | no |
| BRD9 | 27.565 | 26.008 | 18.934 | 20.361 | 26.787 | 19.648 | 0.735 | -0.4435 | 6.617E-10 | 0.000000015 | no |
| SLC25A38 | 11.051 | 8.225 | 6.946 | 7.756 | 9.638 | 7.351 | 0.736 | -0.4422 | 0.00258 | 0.01309 | no |
| RIN3 | 4.826 | 4.419 | 3.625 | 3.009 | 4.622 | 3.317 | 0.736 | -0.4427 | 0.0005633 | 0.0036 | no |
| NKRF | 6.801 | 6.084 | 4.406 | 4.838 | 6.442 | 4.622 | 0.736 | -0.4426 | 0.0002691 | 0.001908 | no |
| LACTB | 48.277 | 48.639 | 32.513 | 35.134 | 48.458 | 33.823 | 0.736 | -0.4415 | 1.627E-08 | 2.893E-07 | no |
| DNAJB6 | 69.143 | 63.454 | 45.185 | 50.547 | 66.299 | 47.866 | 0.736 | -0.442 | 3.282E-09 | 6.63E-08 | no |
| CNOT3 | 20.774 | 19.876 | 14.996 | 14.953 | 20.325 | 14.974 | 0.736 | -0.4413 | 4.261E-09 | 8.424E-08 | no |
| ARHGAP27 | 26.988 | 21.794 | 14.781 | 17.742 | 24.391 | 16.262 | 0.736 | -0.4414 | 2.662E-09 | 5.473E-08 | no |
| TSSC4 | 15.032 | 13.588 | 10.229 | 10.455 | 14.31 | 10.342 | 0.737 | -0.4411 | 0.000498 | 0.003253 | no |
| RHOT2 | 8.612 | 8.342 | 5.891 | 6.487 | 8.477 | 6.189 | 0.737 | -0.4399 | 0.000006758 | 0.00007204 | no |
| PRKRIP1 | 23.511 | 22.008 | 16.853 | 15.293 | 22.759 | 16.073 | 0.737 | -0.4412 | 0.001632 | 0.008886 | no |
| FBXO31 | 20.815 | 18.397 | 15.788 | 14.014 | 19.606 | 14.901 | 0.737 | -0.4412 | 6.144E-08 | 9.919E-07 | no |
| CHAF1B | 7.861 | 7.067 | 5.08 | 5.587 | 7.464 | 5.333 | 0.737 | -0.4407 | 0.000501 | 0.003268 | no |
| ZFP36L1 | 181.943 | 169.366 | 130.464 | 130.68 | 175.655 | 130.572 | 0.738 | -0.4387 | 3.093E-35 | 4.087E-33 | no |
| FBXL3 | 3.591 | 3.339 | 2.394 | 2.399 | 3.465 | 2.397 | 0.738 | -0.4391 | 0.004716 | 0.02163 | no |
| FAM208B | 3.828 | 3.679 | 2.735 | 2.769 | 3.753 | 2.752 | 0.738 | -0.4387 | 0.00001352 | 0.0001326 | no |
| CISH | 3.941 | 2.784 | 1.817 | 2.369 | 3.362 | 2.093 | 0.738 | -0.4375 | 0.01962 | 0.06966 | no |
| ARRDC2 | 8.808 | 6.784 | 5.715 | 5.248 | 7.796 | 5.482 | 0.738 | -0.4382 | 0.002293 | 0.01188 | no |
| TMEM248 | 93.035 | 89.501 | 67.577 | 68.379 | 91.268 | 67.978 | 0.739 | -0.4362 | 5.31E-27 | 4.731E-25 | no |
| TIMM44 | 17.625 | 13.403 | 10.952 | 11.285 | 15.514 | 11.119 | 0.739 | -0.436 | 0.0003232 | 0.002221 | no |
| TGFB1 | 47.052 | 42.731 | 33.5 | 32.525 | 44.892 | 33.013 | 0.739 | -0.4372 | 1.843E-09 | 3.894E-08 | no |
| RPUSD4 | 12.079 | 9.987 | 8.011 | 7.686 | 11.033 | 7.848 | 0.739 | -0.4357 | 0.0008459 | 0.005093 | no |
| PER2 | 0.268 | 0.37 | 0.147 | 0.14 | 0.319 | 0.144 | 0.739 | -0.4359 | 0.02124 | 0.07414 | no |
| WDR4 | 42.124 | 36.326 | 26.427 | 26.238 | 39.225 | 26.332 | 0.74 | -0.4347 | 3.424E-10 | 7.967E-09 | no |
| SLC16A7 | 4.177 | 4.263 | 2.784 | 3.029 | 4.22 | 2.906 | 0.74 | -0.435 | 0.0002742 | 0.001936 | no |
| RRP1B | 35.045 | 33.62 | 24.825 | 24.069 | 34.332 | 24.447 | 0.74 | -0.4347 | 4.205E-11 | 1.086E-09 | no |
| GRHL3 | 1.502 | 1.012 | 0.772 | 0.54 | 1.257 | 0.656 | 0.74 | -0.4348 | 0.02495 | 0.08389 | no |
| DKC1 | 71.602 | 63.142 | 50.626 | 49.037 | 67.372 | 49.831 | 0.74 | -0.4347 | 1.341E-12 | 4.133E-11 | no |
| PPP1R37 | 42.947 | 46.177 | 37.76 | 38.852 | 44.562 | 38.306 | 0.741 | -0.4326 | 9.909E-09 | 1.829E-07 | no |
| POLR2C | 50.993 | 44.483 | 34.545 | 35.893 | 47.738 | 35.219 | 0.741 | -0.433 | 2.785E-09 | 5.706E-08 | no |
| CADM1 | 17.348 | 14.24 | 11.743 | 11.495 | 15.794 | 11.619 | 0.741 | -0.4329 | 0.000001435 | 0.00001801 | no |
| ZNF202 | 2.048 | 1.869 | 1.329 | 1.449 | 1.958 | 1.389 | 0.742 | -0.4296 | 0.002943 | 0.01469 | no |
| RIOK1 | 7.161 | 6.599 | 5.1 | 5.098 | 6.88 | 5.099 | 0.742 | -0.4312 | 4.216E-07 | 0.000005831 | no |
| PGGT1B | 6.204 | 5.772 | 4.435 | 4.028 | 5.988 | 4.231 | 0.742 | -0.4306 | 0.001577 | 0.008657 | no |
| NFKB1 | 8.705 | 8.128 | 6.213 | 6.067 | 8.416 | 6.14 | 0.742 | -0.4313 | 0.00006971 | 0.0005823 | no |
| CPNE1 | 57.475 | 56.202 | 46.337 | 47.358 | 56.838 | 46.847 | 0.742 | -0.4309 | 1.627E-23 | 1.238E-21 | no |
| TAF13 | 16.74 | 15.992 | 11.518 | 12.084 | 16.366 | 11.801 | 0.743 | -0.4289 | 0.0005338 | 0.003451 | no |
| PEAR1 | 48.925 | 44.541 | 34.35 | 35.404 | 46.733 | 34.877 | 0.743 | -0.4287 | 1.044E-11 | 2.927E-10 | no |
| PALB2 | 8.55 | 7.651 | 5.979 | 5.847 | 8.101 | 5.913 | 0.743 | -0.4282 | 0.00009806 | 0.0007849 | no |
| NECAP2 | 25.27 | 23.643 | 17.761 | 18.721 | 24.456 | 18.241 | 0.743 | -0.4291 | 1.194E-11 | 3.307E-10 | no |
| MPHOSPH10 | 45.211 | 42.663 | 31.966 | 33.085 | 43.937 | 32.526 | 0.743 | -0.4289 | 2.324E-09 | 4.838E-08 | no |
| FBLIM1 | 27.205 | 25.21 | 19.227 | 19.211 | 26.207 | 19.219 | 0.743 | -0.4283 | 1.038E-08 | 1.905E-07 | no |
| ELK3 | 15.135 | 13.997 | 10.327 | 11.345 | 14.566 | 10.836 | 0.743 | -0.4293 | 0.000002316 | 0.00002811 | no |
| CSRNP1 | 15.249 | 11.671 | 9.525 | 8.726 | 13.46 | 9.126 | 0.743 | -0.429 | 0.0003217 | 0.002213 | no |
| BAG4 | 2.48 | 3.056 | 1.915 | 1.879 | 2.768 | 1.897 | 0.743 | -0.4291 | 0.009418 | 0.03888 | no |
| TNFAIP2 | 24.025 | 24.091 | 20.692 | 17.102 | 24.058 | 18.897 | 0.744 | -0.4258 | 0.00001156 | 0.0001162 | no |
| SFN | 37.329 | 31.119 | 23.818 | 24.359 | 34.224 | 24.089 | 0.744 | -0.4267 | 0.0006426 | 0.004045 | no |
| RSRC2 | 38.512 | 37.961 | 28.381 | 28.247 | 38.236 | 28.314 | 0.744 | -0.4272 | 9.323E-09 | 1.745E-07 | no |
| PARD6B | 14.755 | 13.695 | 9.017 | 10.775 | 14.225 | 9.896 | 0.744 | -0.4268 | 0.00002035 | 0.0001919 | no |
| NT5DC3 | 2.706 | 2.463 | 1.905 | 1.759 | 2.585 | 1.832 | 0.744 | -0.4266 | 0.003453 | 0.01676 | no |
| FOXN1 | 3.941 | 3.855 | 2.814 | 2.649 | 3.898 | 2.732 | 0.744 | -0.426 | 0.005683 | 0.02543 | no |
| E2F4 | 25.97 | 24.509 | 18.963 | 18.192 | 25.239 | 18.578 | 0.744 | -0.4263 | 0.00000324 | 0.00003792 | no |
| CD274 | 4.784 | 4.215 | 3.068 | 3.338 | 4.499 | 3.203 | 0.744 | -0.4273 | 0.002212 | 0.01153 | no |
| ADGRG6 | 9.95 | 9.529 | 7.161 | 7.197 | 9.739 | 7.179 | 0.744 | -0.4268 | 1.985E-08 | 3.466E-07 | no |
| ZSWIM8 | 35.847 | 32.569 | 25.479 | 25.838 | 34.208 | 25.659 | 0.745 | -0.4253 | 7.614E-17 | 3.662E-15 | no |
| TOR2A | 6.472 | 5.558 | 4.308 | 4.528 | 6.015 | 4.418 | 0.745 | -0.4241 | 0.0001033 | 0.0008216 | no |
| TLR4 | 1.492 | 1.489 | 0.977 | 0.85 | 1.49 | 0.913 | 0.745 | -0.4244 | 0.02521 | 0.08456 | no |
| HNRNPAB | 348.401 | 323.985 | 247.299 | 251.494 | 336.193 | 249.397 | 0.745 | -0.425 | 5.085E-25 | 4.145E-23 | no |
| FZD9 | 2.655 | 2.278 | 1.602 | 1.249 | 2.466 | 1.425 | 0.745 | -0.4254 | 0.02826 | 0.09245 | no |
| PSPC1 | 57.311 | 52.182 | 39.616 | 38.682 | 54.746 | 39.149 | 0.746 | -0.4234 | 2.495E-11 | 6.639E-10 | no |
| CASC3 | 46.579 | 45.427 | 35.327 | 33.685 | 46.003 | 34.506 | 0.746 | -0.4221 | 1.001E-14 | 4.008E-13 | no |
| ZNF620 | 7.665 | 7.125 | 5.11 | 4.288 | 7.395 | 4.699 | 0.747 | -0.4211 | 0.002196 | 0.01146 | no |
| TOP3A | 21.072 | 18.689 | 14.508 | 15.213 | 19.88 | 14.86 | 0.747 | -0.4207 | 8.986E-09 | 1.692E-07 | no |
| TGFB1I1 | 43.822 | 40.988 | 31.927 | 31.146 | 42.405 | 31.537 | 0.747 | -0.4217 | 2.769E-08 | 4.736E-07 | no |
| TFAP2C | 20.177 | 18.572 | 14.254 | 14.613 | 19.374 | 14.433 | 0.747 | -0.42 | 3.124E-07 | 0.000004482 | no |
| POGK | 16.144 | 14.338 | 11.499 | 11.085 | 15.241 | 11.292 | 0.747 | -0.4206 | 6.073E-07 | 0.000008222 | no |
| OLFM1 | 48.081 | 47.111 | 39.118 | 37.933 | 47.596 | 38.526 | 0.747 | -0.4201 | 1.399E-09 | 3.035E-08 | no |
| NSUN2 | 67.744 | 56.747 | 47.334 | 45.709 | 62.245 | 46.522 | 0.747 | -0.4212 | 9.621E-12 | 2.711E-10 | no |
| DENND2C | 3.622 | 3.572 | 2.657 | 2.509 | 3.597 | 2.583 | 0.747 | -0.4207 | 0.002195 | 0.01146 | no |
| CCDC102A | 13.057 | 13.501 | 9.731 | 10.285 | 13.279 | 10.008 | 0.747 | -0.4207 | 0.00003383 | 0.0003043 | no |
| UBTF | 27.596 | 27.653 | 21.249 | 21.6 | 27.624 | 21.425 | 0.748 | -0.4185 | 1.157E-11 | 3.221E-10 | no |
| SNRNP40 | 50.201 | 43.149 | 32.435 | 36.073 | 46.675 | 34.254 | 0.748 | -0.4197 | 0.000005762 | 0.00006272 | no |
| PRKD3 | 21.124 | 20.324 | 15.944 | 15.263 | 20.724 | 15.604 | 0.748 | -0.4192 | 6.79E-14 | 2.502E-12 | no |
| NCOA5 | 17.975 | 16.567 | 13.228 | 12.834 | 17.271 | 13.031 | 0.748 | -0.4186 | 5.965E-07 | 0.000008095 | no |
| KRR1 | 40.797 | 37.514 | 28.908 | 28.417 | 39.156 | 28.663 | 0.748 | -0.4195 | 0.00001884 | 0.0001787 | no |
| TFPT | 8.242 | 8.858 | 6.507 | 5.577 | 8.55 | 6.042 | 0.749 | -0.4164 | 0.005741 | 0.02563 | no |
| SRSF10 | 22.626 | 20.898 | 16.882 | 16.273 | 21.762 | 16.578 | 0.749 | -0.4166 | 1.482E-10 | 3.613E-09 | no |
| GMPPB | 31.073 | 29.96 | 22.011 | 24.029 | 30.517 | 23.02 | 0.749 | -0.4161 | 0.000002939 | 0.00003485 | no |
| EXOSC3 | 10.474 | 9.695 | 7.63 | 7.067 | 10.085 | 7.348 | 0.749 | -0.4161 | 0.0008139 | 0.004933 | no |
| ABL2 | 4.898 | 5.032 | 3.82 | 3.608 | 4.965 | 3.714 | 0.749 | -0.4161 | 0.000002607 | 0.00003128 | no |
| RRP15 | 11.411 | 10.016 | 7.708 | 8.226 | 10.713 | 7.967 | 0.75 | -0.4146 | 0.00001719 | 0.0001646 | no |
| POLR3D | 5.896 | 5.032 | 3.761 | 4.288 | 5.464 | 4.024 | 0.75 | -0.4159 | 0.0004067 | 0.002719 | no |
| OTUD4 | 37.782 | 36.229 | 28.322 | 28.547 | 37.005 | 28.434 | 0.75 | -0.4153 | 2.732E-13 | 9.318E-12 | no |
| INHBA | 3.828 | 4.234 | 2.491 | 1.679 | 4.031 | 2.085 | 0.75 | -0.4152 | 0.03273 | 0.1035 | no |
| FCGR3A | 5.968 | 4.653 | 3.8 | 3.428 | 5.31 | 3.614 | 0.75 | -0.4143 | 0.01524 | 0.05706 | no |
| TRAPPC12 | 6.451 | 5.986 | 4.689 | 4.648 | 6.218 | 4.668 | 0.751 | -0.4138 | 0.00004214 | 0.0003692 | no |
| TNFAIP1 | 30.929 | 29.123 | 23.545 | 21.61 | 30.026 | 22.578 | 0.751 | -0.4122 | 1.959E-09 | 4.124E-08 | no |
| MEDAG | 51.415 | 45.213 | 34.35 | 37.423 | 48.314 | 35.886 | 0.751 | -0.4125 | 9.039E-08 | 0.000001423 | no |
| CTH | 4.27 | 4.516 | 3.126 | 2.939 | 4.393 | 3.032 | 0.751 | -0.4127 | 0.01392 | 0.05316 | no |
| ADAMTS16 | 17.018 | 16.576 | 12.271 | 12.764 | 16.797 | 12.518 | 0.751 | -0.4135 | 4.155E-07 | 0.000005755 | no |
| ZNF330 | 19.632 | 18.65 | 13.961 | 14.283 | 19.141 | 14.122 | 0.752 | -0.4113 | 0.0001333 | 0.001024 | no |
| WDR12 | 14.971 | 13.617 | 10.962 | 10.505 | 14.294 | 10.733 | 0.752 | -0.4111 | 1.918E-07 | 0.000002862 | no |
| SMAD3 | 43.605 | 42.731 | 32.933 | 33.115 | 43.168 | 33.024 | 0.752 | -0.4111 | 3.206E-18 | 1.746E-16 | no |
| POP1 | 6.935 | 6.278 | 5.217 | 4.528 | 6.606 | 4.872 | 0.752 | -0.4119 | 0.0002768 | 0.001952 | no |
| KANK1 | 13.88 | 11.933 | 9.691 | 9.786 | 12.907 | 9.739 | 0.752 | -0.4115 | 6.184E-07 | 0.000008352 | no |
| FBXL5 | 62.486 | 57.195 | 45.497 | 44.669 | 59.84 | 45.083 | 0.752 | -0.4109 | 3.974E-13 | 1.324E-11 | no |
| DIABLO | 29.19 | 29.094 | 20.272 | 22.999 | 29.142 | 21.636 | 0.752 | -0.4107 | 0.00002938 | 0.0002683 | no |
| ALKBH3 | 13.983 | 13.199 | 9.75 | 9.766 | 13.591 | 9.758 | 0.752 | -0.4116 | 0.002818 | 0.01416 | no |
| RMND5B | 22.708 | 19.574 | 15.475 | 15.693 | 21.141 | 15.584 | 0.753 | -0.4084 | 0.000002815 | 0.00003353 | no |
| NUAK2 | 3.786 | 3.592 | 2.374 | 2.999 | 3.689 | 2.687 | 0.753 | -0.409 | 0.00293 | 0.01463 | no |
| DDX42 | 20.578 | 20.46 | 15.934 | 15.093 | 20.519 | 15.514 | 0.753 | -0.4094 | 1.631E-10 | 3.939E-09 | no |
| STRADB | 12.882 | 11.213 | 8.138 | 9.326 | 12.047 | 8.732 | 0.754 | -0.4069 | 0.00102 | 0.00599 | no |
| GTF2F1 | 75.018 | 69.128 | 53.45 | 55.125 | 72.073 | 54.288 | 0.754 | -0.4078 | 1.519E-10 | 3.685E-09 | no |
| CDC42EP3 | 36.022 | 35.752 | 28.058 | 28.877 | 35.887 | 28.468 | 0.754 | -0.4065 | 2.912E-07 | 0.000004221 | no |
| AGAP1 | 8.19 | 7.398 | 5.93 | 6.537 | 7.794 | 6.233 | 0.754 | -0.407 | 6.501E-07 | 0.000008729 | no |
| UMPS | 51.775 | 49.846 | 38.932 | 37.613 | 50.81 | 38.273 | 0.755 | -0.4055 | 1.523E-08 | 2.726E-07 | no |
| RAPGEF2 | 14.446 | 14.377 | 10.766 | 11.075 | 14.412 | 10.921 | 0.755 | -0.4056 | 3.323E-09 | 0.000000067 | no |
| UACA | 13.211 | 13.072 | 10.336 | 9.786 | 13.142 | 10.061 | 0.756 | -0.4037 | 3.974E-10 | 9.172E-09 | no |
| SRP68 | 104.126 | 94.222 | 74.445 | 76.215 | 99.174 | 75.33 | 0.756 | -0.4033 | 8.662E-17 | 4.148E-15 | no |
| SEC14L1 | 58.021 | 50.819 | 42.127 | 43.25 | 54.42 | 42.689 | 0.756 | -0.4037 | 3.776E-16 | 1.75E-14 | no |
| MPP6 | 2.305 | 2.404 | 1.827 | 1.629 | 2.354 | 1.728 | 0.756 | -0.4034 | 0.002427 | 0.01243 | no |
| MED8 | 26.639 | 24.821 | 19.451 | 17.522 | 25.73 | 18.486 | 0.756 | -0.4043 | 0.001278 | 0.007224 | no |
| MAD1L1 | 11.688 | 11.203 | 8.392 | 8.906 | 11.445 | 8.649 | 0.756 | -0.4032 | 0.000001215 | 0.00001546 | no |
| IL10RA | 2.367 | 2.472 | 1.71 | 1.659 | 2.42 | 1.684 | 0.756 | -0.404 | 0.01658 | 0.0609 | no |
| DLST | 138.06 | 126.294 | 100.696 | 100.314 | 132.177 | 100.505 | 0.756 | -0.4035 | 2.464E-21 | 1.647E-19 | no |
| WDR26 | 22.667 | 22.524 | 16.774 | 17.612 | 22.596 | 17.193 | 0.757 | -0.4021 | 8.591E-12 | 2.439E-10 | no |
| SEMA3D | 0.401 | 0.526 | 0.283 | 0.23 | 0.464 | 0.257 | 0.757 | -0.4025 | 0.03805 | 0.1164 | no |
| PTP4A1 | 24.807 | 23.283 | 18.406 | 18.222 | 24.045 | 18.314 | 0.757 | -0.4009 | 1.327E-09 | 2.904E-08 | no |
| PPTC7 | 10.783 | 10.201 | 8.265 | 7.507 | 10.492 | 7.886 | 0.757 | -0.4022 | 0.00001331 | 0.000131 | no |
| KBTBD8 | 0.638 | 0.603 | 0.469 | 0.26 | 0.621 | 0.364 | 0.757 | -0.4017 | 0.03886 | 0.1182 | no |
| ERF | 27.328 | 24.568 | 20.34 | 18.761 | 25.948 | 19.55 | 0.757 | -0.4019 | 8.323E-07 | 0.00001092 | no |
| DDX3X | 227.071 | 215.036 | 167.373 | 170.961 | 221.053 | 169.167 | 0.757 | -0.4016 | 9.547E-46 | 1.742E-43 | no |
| CORO1C | 67.384 | 61.234 | 48.926 | 48.977 | 64.309 | 48.951 | 0.757 | -0.4016 | 7.286E-17 | 3.519E-15 | no |
| BYSL | 16.679 | 16.265 | 12.759 | 12.204 | 16.472 | 12.482 | 0.758 | -0.4 | 1.963E-07 | 0.000002927 | no |
| AMMECR1L | 22.811 | 22.465 | 17.165 | 17.332 | 22.638 | 17.248 | 0.758 | -0.3997 | 9.73E-10 | 2.172E-08 | no |
| ZC3H3 | 33.81 | 29.61 | 23.828 | 24.279 | 31.71 | 24.053 | 0.759 | -0.3987 | 0.00003278 | 0.0002962 | no |
| WDR36 | 12.306 | 12.586 | 9.408 | 9.506 | 12.446 | 9.457 | 0.759 | -0.3979 | 2.643E-07 | 0.000003866 | no |
| SS18 | 46.805 | 44.561 | 34.653 | 34.254 | 45.683 | 34.453 | 0.759 | -0.397 | 3.828E-12 | 1.131E-10 | no |
| SENP5 | 13.232 | 12.099 | 9.828 | 9.386 | 12.665 | 9.607 | 0.759 | -0.3979 | 1.895E-07 | 0.000002832 | no |
| MEFV | 4.784 | 5.139 | 3.546 | 4.648 | 4.962 | 4.097 | 0.759 | -0.397 | 0.003306 | 0.01622 | no |
| YWHAG | 173.774 | 161.988 | 127.122 | 130.54 | 167.881 | 128.831 | 0.76 | -0.3957 | 6.437E-29 | 6.217E-27 | no |
| TXLNG | 10.022 | 9.675 | 7.454 | 8.316 | 9.849 | 7.885 | 0.76 | -0.3964 | 0.00005758 | 0.0004891 | no |
| PPP6C | 13.746 | 13.072 | 10.629 | 9.636 | 13.409 | 10.133 | 0.76 | -0.3959 | 0.00001154 | 0.0001161 | no |
| KMT5B | 7.871 | 8.449 | 5.735 | 5.777 | 8.16 | 5.756 | 0.76 | -0.3957 | 0.0005527 | 0.003556 | no |
| CDK12 | 9.548 | 10.201 | 7.571 | 7.457 | 9.875 | 7.514 | 0.76 | -0.3959 | 2.073E-07 | 0.000003087 | no |
| YEATS2 | 8.859 | 8.235 | 6.536 | 6.417 | 8.547 | 6.476 | 0.761 | -0.3941 | 0.000006111 | 0.00006613 | no |
| RAD54L2 | 6.935 | 7.719 | 5.471 | 5.567 | 7.327 | 5.519 | 0.761 | -0.3948 | 0.0001818 | 0.001351 | no |
| NAA15 | 26.875 | 24.101 | 19.539 | 19.481 | 25.488 | 19.51 | 0.761 | -0.3948 | 7.43E-12 | 2.124E-10 | no |
| CTDP1 | 13.222 | 13.948 | 10.278 | 9.296 | 13.585 | 9.787 | 0.761 | -0.3931 | 0.00003585 | 0.0003204 | no |
| YARS2 | 7.933 | 6.794 | 5.383 | 5.687 | 7.364 | 5.535 | 0.762 | -0.393 | 0.0002222 | 0.001612 | no |
| RNPS1 | 139.367 | 128.134 | 101.086 | 101.303 | 133.75 | 101.195 | 0.762 | -0.3926 | 4.563E-17 | 2.261E-15 | no |
| OSR1 | 17.666 | 14.659 | 11.03 | 13.004 | 16.163 | 12.017 | 0.762 | -0.3917 | 0.001138 | 0.006568 | no |
| FGF2 | 60.274 | 61.702 | 48.799 | 48.128 | 60.988 | 48.463 | 0.762 | -0.3922 | 3.414E-15 | 1.438E-13 | no |
| AKAP12 | 74.555 | 77.431 | 59.087 | 59.813 | 75.993 | 59.45 | 0.762 | -0.3923 | 3.13E-23 | 2.306E-21 | no |
| TWNK | 6.328 | 5.714 | 3.996 | 4.358 | 6.021 | 4.177 | 0.763 | -0.3893 | 0.0000824 | 0.0006727 | no |
| THOP1 | 79.185 | 66.822 | 55.032 | 56.524 | 73.004 | 55.778 | 0.763 | -0.3893 | 3.38E-10 | 7.896E-09 | no |
| STAC2 | 40.117 | 37.806 | 29.123 | 30.586 | 38.962 | 29.855 | 0.763 | -0.3898 | 2.577E-10 | 6.119E-09 | no |
| SLC35D1 | 1.852 | 1.986 | 1.436 | 1.389 | 1.919 | 1.413 | 0.763 | -0.3901 | 0.005155 | 0.02333 | no |
| EVPL | 12.841 | 12.294 | 9.086 | 9.566 | 12.567 | 9.326 | 0.763 | -0.3906 | 0.000003953 | 0.00004508 | no |
| DUSP11 | 37.401 | 33.542 | 25.802 | 27.457 | 35.472 | 26.63 | 0.763 | -0.3903 | 0.00005916 | 0.0005018 | no |
| ZBTB49 | 6.883 | 6.492 | 4.66 | 5.058 | 6.688 | 4.859 | 0.764 | -0.3879 | 0.00319 | 0.01578 | no |
| WNT7A | 27.678 | 24.967 | 19.784 | 18.701 | 26.322 | 19.242 | 0.764 | -0.389 | 0.0000128 | 0.0001266 | no |
| UTP3 | 21.618 | 21.21 | 15.7 | 16.153 | 21.414 | 15.926 | 0.764 | -0.3886 | 0.0009741 | 0.00578 | no |
| RPL7L1 | 76.448 | 69.274 | 55.062 | 55.584 | 72.861 | 55.323 | 0.764 | -0.3883 | 2.847E-08 | 4.863E-07 | no |
| MDN1 | 6.379 | 6.346 | 4.816 | 5.378 | 6.362 | 5.097 | 0.764 | -0.3888 | 3.481E-07 | 0.000004897 | no |
| MAPK8 | 9.507 | 8.897 | 7.21 | 6.817 | 9.202 | 7.014 | 0.764 | -0.3888 | 0.0000103 | 0.0001045 | no |
| INO80 | 14.004 | 13.15 | 10.571 | 10.185 | 13.577 | 10.378 | 0.764 | -0.3878 | 5.166E-08 | 8.459E-07 | no |
| C11orf68 | 80.245 | 72.175 | 58.725 | 57.624 | 76.21 | 58.175 | 0.764 | -0.3877 | 1.566E-08 | 2.795E-07 | no |
| ARHGEF10 | 16.298 | 14.863 | 11.655 | 12.264 | 15.58 | 11.959 | 0.764 | -0.3875 | 0.000000276 | 0.000004011 | no |
| ZBTB46 | 14.065 | 12.946 | 8.49 | 10.575 | 13.505 | 9.532 | 0.765 | -0.3863 | 0.0006994 | 0.004343 | no |
| YAP1 | 75.728 | 75.562 | 57.748 | 59.113 | 75.645 | 58.43 | 0.765 | -0.3866 | 7.86E-22 | 5.41E-20 | no |
| WDR75 | 25.702 | 23.351 | 18.504 | 18.871 | 24.526 | 18.688 | 0.765 | -0.3859 | 0.000003362 | 0.00003923 | no |
| SRPRB | 18.407 | 16.537 | 13.58 | 13.174 | 17.472 | 13.377 | 0.765 | -0.3856 | 2.751E-07 | 0.000004002 | no |
| NUFIP1 | 25.034 | 26.738 | 17.712 | 20.011 | 25.886 | 18.861 | 0.765 | -0.3867 | 0.006304 | 0.02777 | no |
| NOL8 | 14.631 | 13.578 | 10.864 | 10.905 | 14.104 | 10.884 | 0.765 | -0.3869 | 0.000001981 | 0.0000244 | no |
| NECTIN2 | 77.241 | 68.671 | 58.217 | 57.504 | 72.956 | 57.861 | 0.765 | -0.3863 | 1.731E-12 | 5.277E-11 | no |
| MGAT2 | 58.103 | 54.937 | 43.143 | 40.881 | 56.52 | 42.012 | 0.765 | -0.3857 | 7.129E-08 | 0.000001138 | no |
| BANP | 4.383 | 4.361 | 3.234 | 3.079 | 4.372 | 3.157 | 0.765 | -0.387 | 0.01325 | 0.05099 | no |
| ZNF622 | 33.203 | 26.661 | 20.868 | 24.149 | 29.932 | 22.508 | 0.766 | -0.3837 | 0.0002725 | 0.001928 | no |
| ZNF142 | 5.741 | 5.548 | 4.279 | 4.288 | 5.644 | 4.284 | 0.766 | -0.3855 | 0.00007299 | 0.0006057 | no |
| RAD54L | 6.39 | 6.288 | 4.455 | 4.988 | 6.339 | 4.722 | 0.766 | -0.3839 | 0.003631 | 0.01748 | no |
| CDC42BPB | 61.251 | 58.052 | 46.972 | 46.639 | 59.651 | 46.806 | 0.766 | -0.3839 | 6.436E-14 | 2.379E-12 | no |
| SLC25A29 | 27.616 | 22.67 | 21.483 | 20.601 | 25.143 | 21.042 | 0.767 | -0.3824 | 0.00001625 | 0.0001568 | no |
| NET1 | 18.13 | 16.917 | 14.029 | 12.714 | 17.523 | 13.372 | 0.767 | -0.383 | 0.000009342 | 0.0000963 | no |
| PRPF38A | 13.767 | 12.206 | 9.535 | 10.295 | 12.986 | 9.915 | 0.768 | -0.3807 | 0.0000492 | 0.0004249 | no |
| MTPAP | 2.891 | 3.086 | 2.13 | 2.249 | 2.989 | 2.189 | 0.768 | -0.3802 | 0.01269 | 0.04925 | no |
| LRRC8D | 37.473 | 35.742 | 27.57 | 28.707 | 36.608 | 28.139 | 0.768 | -0.3807 | 5.364E-08 | 8.758E-07 | no |
| EXOSC10 | 42.638 | 36.861 | 30.335 | 30.756 | 39.749 | 30.546 | 0.768 | -0.381 | 3.343E-08 | 0.000000561 | no |
| YRDC | 23.83 | 20.509 | 17.4 | 15.953 | 22.169 | 16.676 | 0.769 | -0.3788 | 0.0006523 | 0.004099 | no |
| MARK3 | 38.739 | 35.654 | 29.143 | 28.027 | 37.197 | 28.585 | 0.769 | -0.379 | 2.45E-08 | 4.241E-07 | no |
| GPRC5D | 0.597 | 0.496 | 0.303 | 0.3 | 0.546 | 0.301 | 0.769 | -0.3795 | 0.04929 | 0.1414 | no |
| CPOX | 28.768 | 28.023 | 21.747 | 22.27 | 28.395 | 22.008 | 0.769 | -0.3785 | 1.916E-10 | 4.587E-09 | no |
| TXLNA | 43.822 | 41.28 | 33.148 | 32.805 | 42.551 | 32.977 | 0.77 | -0.3778 | 5.256E-15 | 2.173E-13 | no |
| TRMT44 | 4.651 | 4.877 | 4.035 | 2.739 | 4.764 | 3.387 | 0.77 | -0.3779 | 0.02375 | 0.08076 | no |
| SEH1L | 7.511 | 6.658 | 5.403 | 5.348 | 7.085 | 5.375 | 0.77 | -0.3774 | 0.001014 | 0.005966 | no |
| ELP2 | 9.816 | 9.189 | 7.591 | 6.917 | 9.503 | 7.254 | 0.77 | -0.3775 | 0.0001241 | 0.0009645 | no |
| CSF1 | 65.285 | 60.709 | 47.5 | 49.597 | 62.997 | 48.549 | 0.77 | -0.3763 | 3.468E-15 | 1.455E-13 | no |
| CDKN1A | 10.33 | 10.688 | 7.611 | 8.356 | 10.509 | 7.983 | 0.77 | -0.3762 | 0.00006557 | 0.0005505 | no |
| AEN | 137.422 | 121.408 | 100.471 | 98.935 | 129.415 | 99.703 | 0.77 | -0.3775 | 1.896E-17 | 9.908E-16 | no |
| UBE2QL1 | 10.238 | 8.984 | 6.653 | 7.517 | 9.611 | 7.085 | 0.771 | -0.3754 | 0.009607 | 0.03943 | no |
| RHOJ | 31.907 | 28.997 | 22.372 | 24.079 | 30.452 | 23.226 | 0.771 | -0.3752 | 0.00009232 | 0.0007452 | no |
| RBM23 | 41.342 | 36.832 | 29.836 | 30.336 | 39.087 | 30.086 | 0.771 | -0.3753 | 1.716E-08 | 0.000000303 | no |
| PTGES3L | 2.624 | 2.239 | 1.68 | 1.649 | 2.431 | 1.664 | 0.771 | -0.3745 | 0.04055 | 0.1219 | no |
| PDGFA | 38.965 | 33.591 | 28.205 | 27.657 | 36.278 | 27.931 | 0.771 | -0.3759 | 0.000004383 | 0.00004929 | no |
| HRH2 | 9.445 | 9.062 | 6.78 | 7.347 | 9.253 | 7.064 | 0.771 | -0.3761 | 0.0000788 | 0.0006479 | no |
| FAM126B | 2.634 | 2.56 | 1.973 | 1.599 | 2.597 | 1.786 | 0.771 | -0.3756 | 0.03663 | 0.113 | no |
| EIF1 | 657.22 | 596.09 | 475.341 | 490.015 | 626.655 | 482.678 | 0.771 | -0.376 | 5.147E-28 | 4.849E-26 | no |
| CHERP | 30.754 | 25.619 | 21.933 | 21.32 | 28.187 | 21.627 | 0.771 | -0.3761 | 0.000004281 | 0.00004825 | no |
| C1orf131 | 16.524 | 15.817 | 12.427 | 12.164 | 16.171 | 12.296 | 0.771 | -0.3746 | 0.0003816 | 0.002567 | no |
| TAB3 | 17.975 | 17.306 | 13.56 | 13.924 | 17.641 | 13.742 | 0.772 | -0.3741 | 8.178E-10 | 1.836E-08 | no |
| SNAI2 | 10.845 | 9.666 | 7.357 | 6.827 | 10.256 | 7.092 | 0.772 | -0.3727 | 0.005395 | 0.02428 | no |
| SIAH2 | 5.206 | 4.147 | 3.8 | 2.809 | 4.677 | 3.304 | 0.772 | -0.3726 | 0.02936 | 0.09515 | no |
| MRM1 | 8.941 | 7.943 | 6.184 | 6.607 | 8.442 | 6.396 | 0.772 | -0.3726 | 0.001791 | 0.009581 | no |
| MAP3K7 | 12.944 | 12.975 | 9.77 | 10.185 | 12.96 | 9.977 | 0.772 | -0.3741 | 0.000003148 | 0.00003693 | no |
| FUBP3 | 29.314 | 27.03 | 21.698 | 21.83 | 28.172 | 21.764 | 0.772 | -0.3729 | 0.000000161 | 0.000002451 | no |
| FKRP | 3.858 | 3.339 | 2.579 | 2.809 | 3.599 | 2.694 | 0.772 | -0.3741 | 0.005706 | 0.0255 | no |
| DGCR8 | 12.481 | 11.408 | 9.691 | 8.676 | 11.944 | 9.184 | 0.772 | -0.3731 | 0.00002755 | 0.0002527 | no |
| THOC3 | 53.03 | 49.973 | 39.635 | 39.362 | 51.502 | 39.498 | 0.773 | -0.3712 | 0.000002941 | 0.00003485 | no |
| THOC1 | 6.822 | 6.95 | 5.608 | 4.828 | 6.886 | 5.218 | 0.773 | -0.3706 | 0.002103 | 0.01105 | no |
| SUGP1 | 17.265 | 16.022 | 12.114 | 13.054 | 16.643 | 12.584 | 0.773 | -0.3714 | 0.0004372 | 0.002898 | no |
| SLC3A2 | 58.926 | 54.869 | 43.436 | 44.649 | 56.898 | 44.043 | 0.773 | -0.3712 | 2.886E-09 | 5.891E-08 | no |
| PHF19 | 19.436 | 16.645 | 13.98 | 13.784 | 18.041 | 13.882 | 0.773 | -0.3713 | 0.000006686 | 0.00007135 | no |
| HSPA5 | 227.812 | 208.398 | 168.477 | 171.861 | 218.105 | 170.169 | 0.773 | -0.3716 | 7.119E-30 | 7.306E-28 | no |
| GCNT2 | 8.766 | 8.585 | 6.458 | 7.087 | 8.675 | 6.772 | 0.773 | -0.3723 | 0.0004424 | 0.002928 | no |
| DCK | 20.908 | 19.156 | 15.407 | 15.533 | 20.032 | 15.47 | 0.773 | -0.3722 | 0.000002083 | 0.00002552 | no |
| CEBPZ | 19.086 | 18.338 | 14.127 | 15.073 | 18.712 | 14.6 | 0.773 | -0.3712 | 0.000007689 | 0.00008093 | no |
| ZHX1 | 7.429 | 8.225 | 6.487 | 5.957 | 7.827 | 6.222 | 0.774 | -0.3703 | 0.0008948 | 0.005362 | no |
| SRRT | 79.823 | 73.392 | 56.791 | 57.753 | 76.607 | 57.272 | 0.774 | -0.3694 | 1.947E-17 | 1.012E-15 | no |
| PUS3 | 8.18 | 8.41 | 6.448 | 5.997 | 8.295 | 6.223 | 0.774 | -0.3693 | 0.01062 | 0.04284 | no |
| PISD | 22.821 | 21.453 | 17.576 | 16.592 | 22.137 | 17.084 | 0.774 | -0.3698 | 0.00003415 | 0.0003069 | no |
| LRRC57 | 9.054 | 8.692 | 7.288 | 6.647 | 8.873 | 6.968 | 0.774 | -0.3702 | 0.00006152 | 0.0005199 | no |
| LRRC4C | 3.725 | 4.341 | 2.579 | 3.029 | 4.033 | 2.804 | 0.774 | -0.3703 | 0.04014 | 0.1209 | no |
| FASTKD3 | 3.581 | 2.901 | 2.179 | 2.369 | 3.241 | 2.274 | 0.774 | -0.3701 | 0.03555 | 0.1103 | no |
| EIF4ENIF1 | 12.049 | 10.512 | 8.597 | 8.876 | 11.28 | 8.736 | 0.774 | -0.3689 | 0.000002336 | 0.00002833 | no |
| PTRH1 | 21.803 | 21.375 | 17.224 | 15.483 | 21.589 | 16.354 | 0.775 | -0.3678 | 0.001885 | 0.01001 | no |
| PRRG1 | 1.862 | 1.83 | 1.211 | 1.429 | 1.846 | 1.32 | 0.775 | -0.3681 | 0.03328 | 0.1049 | no |
| PPP2CB | 75.193 | 70.472 | 55.833 | 57.264 | 72.832 | 56.549 | 0.775 | -0.3669 | 6.087E-10 | 1.388E-08 | no |
| OSMR | 135.138 | 130.723 | 103.382 | 104.952 | 132.93 | 104.167 | 0.775 | -0.3675 | 1.334E-29 | 1.345E-27 | no |
| GEM | 60.891 | 55.385 | 45.8 | 44.17 | 58.138 | 44.985 | 0.775 | -0.3674 | 1.849E-08 | 3.249E-07 | no |
| CPSF4 | 8.437 | 7.466 | 5.705 | 6.297 | 7.951 | 6.001 | 0.775 | -0.3684 | 0.004531 | 0.02093 | no |
| CDC25A | 6.06 | 6.327 | 4.328 | 5.058 | 6.194 | 4.693 | 0.775 | -0.3676 | 0.003006 | 0.01496 | no |
| BIRC3 | 50.911 | 48.123 | 37.701 | 39.252 | 49.517 | 38.477 | 0.775 | -0.3675 | 3.695E-13 | 1.242E-11 | no |
| UTP4 | 31.269 | 29.746 | 23.428 | 23.949 | 30.508 | 23.689 | 0.776 | -0.3663 | 2.952E-07 | 0.000004268 | no |
| RRP12 | 19.364 | 17.783 | 14.195 | 14.723 | 18.574 | 14.459 | 0.776 | -0.3652 | 2.74E-09 | 5.624E-08 | no |
| HTR7 | 3.313 | 3.037 | 2.179 | 2.409 | 3.175 | 2.294 | 0.776 | -0.3658 | 0.02935 | 0.09515 | no |
| CYTH1 | 15.773 | 13.958 | 11.245 | 11.645 | 14.866 | 11.445 | 0.776 | -0.3652 | 0.000172 | 0.001287 | no |
| CX3CL1 | 46.147 | 41.125 | 34.77 | 33.774 | 43.636 | 34.272 | 0.776 | -0.3656 | 7.157E-07 | 0.000009554 | no |
| CREBBP | 18.747 | 18.951 | 14.381 | 15.103 | 18.849 | 14.742 | 0.776 | -0.3654 | 2.979E-09 | 6.072E-08 | no |
| RBM22 | 20.969 | 19.01 | 14.977 | 16.193 | 19.989 | 15.585 | 0.777 | -0.3648 | 6.868E-08 | 0.000001099 | no |
| RBM14 | 97.356 | 90.659 | 73.429 | 74.146 | 94.007 | 73.787 | 0.777 | -0.3645 | 3.156E-15 | 1.335E-13 | no |
| PRPF4 | 10.186 | 9.549 | 7.591 | 7.746 | 9.867 | 7.668 | 0.777 | -0.3635 | 0.00001034 | 0.0001048 | no |
| NUP160 | 10.948 | 11.369 | 8.255 | 9.166 | 11.159 | 8.71 | 0.777 | -0.3641 | 0.000001574 | 0.00001958 | no |
| MTERF3 | 15.588 | 13.296 | 10.092 | 11.665 | 14.442 | 10.878 | 0.777 | -0.3647 | 0.002626 | 0.01328 | no |
| EIF5A2 | 7.161 | 7.008 | 5.315 | 5.587 | 7.085 | 5.451 | 0.777 | -0.3647 | 0.0003223 | 0.002216 | no |
| ZP1 | 6.276 | 5.023 | 4.494 | 4.188 | 5.649 | 4.341 | 0.778 | -0.362 | 0.0003578 | 0.002429 | no |
| SLC25A19 | 20.249 | 18.523 | 15.153 | 14.963 | 19.386 | 15.058 | 0.778 | -0.3622 | 0.00007513 | 0.0006203 | no |
| POLR1B | 12.388 | 11.203 | 9.477 | 8.816 | 11.796 | 9.146 | 0.778 | -0.3626 | 0.00004206 | 0.000369 | no |
| CCDC137 | 40.416 | 34.146 | 29.524 | 27.737 | 37.281 | 28.63 | 0.778 | -0.3622 | 0.0001454 | 0.001107 | no |
| BRPF3 | 14.127 | 13.627 | 10.297 | 11.065 | 13.877 | 10.681 | 0.778 | -0.3624 | 5.954E-07 | 0.000008089 | no |
| TEF | 13.962 | 15.467 | 11.059 | 11.595 | 14.715 | 11.327 | 0.779 | -0.3603 | 0.00005107 | 0.0004397 | no |
| RNF25 | 33.676 | 27.673 | 22.372 | 24.369 | 30.675 | 23.37 | 0.779 | -0.3606 | 0.00075 | 0.004595 | no |
| PHF23 | 53.473 | 50.547 | 39.928 | 41.101 | 52.01 | 40.514 | 0.779 | -0.3596 | 1.014E-07 | 0.000001583 | no |
| NOC4L | 7.285 | 7.106 | 5.676 | 5.547 | 7.196 | 5.611 | 0.779 | -0.3595 | 0.00001866 | 0.0001774 | no |
| MTUS1 | 15.629 | 16.226 | 11.948 | 12.514 | 15.927 | 12.231 | 0.779 | -0.3611 | 0.00000206 | 0.00002527 | no |
| KNOP1 | 6.534 | 5.957 | 4.856 | 4.788 | 6.245 | 4.822 | 0.779 | -0.3594 | 0.0002833 | 0.001988 | no |
| HIPK2 | 7.83 | 8.994 | 6.477 | 6.037 | 8.412 | 6.257 | 0.779 | -0.3606 | 0.00008518 | 0.0006934 | no |
| DMAP1 | 18.809 | 17.345 | 13.668 | 14.143 | 18.077 | 13.905 | 0.779 | -0.3605 | 0.000462 | 0.003039 | no |
| VGF | 4.147 | 4.409 | 3.204 | 2.979 | 4.278 | 3.091 | 0.78 | -0.3577 | 0.03523 | 0.1097 | no |
| RRAD | 1.533 | 18.367 | 0.352 | 0.8 | 9.95 | 0.576 | 0.78 | -0.3586 | 0.03478 | 0.1085 | no |
| PHC2 | 109.271 | 94.728 | 76.936 | 78.014 | 101.999 | 77.475 | 0.78 | -0.3593 | 3.488E-12 | 1.041E-10 | no |
| PDE12 | 6.184 | 5.227 | 4.24 | 4.528 | 5.706 | 4.384 | 0.78 | -0.3592 | 0.002238 | 0.01164 | no |
| OPA1 | 7.923 | 8.488 | 6.223 | 6.577 | 8.206 | 6.4 | 0.78 | -0.3576 | 0.00009251 | 0.0007462 | no |
| MIEF1 | 39.305 | 36.638 | 29.944 | 29.606 | 37.971 | 29.775 | 0.78 | -0.3578 | 5.432E-14 | 2.027E-12 | no |
| MEIS3 | 25.095 | 22.679 | 19.471 | 16.892 | 23.887 | 18.181 | 0.78 | -0.3576 | 0.001004 | 0.00592 | no |
| GTF2H1 | 16.967 | 15.953 | 12.652 | 12.794 | 16.46 | 12.723 | 0.78 | -0.3592 | 0.00006324 | 0.0005329 | no |
| CAMK1D | 20.218 | 21.258 | 21.562 | 16.322 | 20.738 | 18.942 | 0.78 | -0.3589 | 5.639E-07 | 0.000007689 | no |
| BRICD5 | 22.688 | 19.506 | 17.507 | 15.413 | 21.097 | 16.46 | 0.78 | -0.3581 | 0.000003902 | 0.00004453 | no |
| AAR2 | 19.292 | 17.501 | 13.404 | 14.653 | 18.397 | 14.029 | 0.78 | -0.3578 | 0.00009362 | 0.0007536 | no |
| TOP1 | 45.2 | 40.171 | 34.282 | 32.585 | 42.686 | 33.433 | 0.781 | -0.3571 | 7.963E-09 | 0.000000152 | no |
| RRP9 | 34.181 | 29.863 | 25.176 | 23.989 | 32.022 | 24.582 | 0.781 | -0.3571 | 0.0002473 | 0.00177 | no |
| PPIL2 | 23.037 | 22.134 | 17.683 | 18.142 | 22.585 | 17.913 | 0.781 | -0.3571 | 5.279E-07 | 0.000007249 | no |
| DDIT3 | 17.821 | 14.931 | 12.183 | 11.655 | 16.376 | 11.919 | 0.781 | -0.3569 | 0.02292 | 0.07856 | no |
| ZNF582 | 1.636 | 1.694 | 1.055 | 1.279 | 1.665 | 1.167 | 0.782 | -0.3555 | 0.04767 | 0.1379 | no |
| ZDHHC23 | 3.416 | 2.132 | 2.179 | 3.099 | 2.774 | 2.639 | 0.782 | -0.3547 | 0.04497 | 0.132 | no |
| POLR2D | 10.979 | 10.094 | 9.076 | 7.876 | 10.537 | 8.476 | 0.782 | -0.3553 | 0.00003636 | 0.0003245 | no |
| LYAR | 71.787 | 65.478 | 53.753 | 52.906 | 68.632 | 53.329 | 0.782 | -0.3542 | 0.000001411 | 0.00001775 | no |
| INPP5E | 4.033 | 3.884 | 2.941 | 3.189 | 3.958 | 3.065 | 0.782 | -0.3553 | 0.005434 | 0.02445 | no |
| CCAR1 | 40.138 | 39.927 | 31.644 | 31.506 | 40.032 | 31.575 | 0.782 | -0.3545 | 1.467E-11 | 4.014E-10 | no |
| CBFB | 35.58 | 31.381 | 26.036 | 26.348 | 33.48 | 26.192 | 0.782 | -0.3546 | 9.529E-07 | 0.00001238 | no |
| ATP2C2 | 8.067 | 6.395 | 5.11 | 4.958 | 7.231 | 5.034 | 0.782 | -0.3555 | 0.04298 | 0.1273 | no |
| THRAP3 | 17.697 | 17.939 | 13.687 | 13.674 | 17.818 | 13.68 | 0.783 | -0.353 | 2.138E-09 | 4.464E-08 | no |
| RANBP3 | 43.441 | 40.258 | 32.367 | 33.295 | 41.85 | 32.831 | 0.783 | -0.3532 | 8.847E-09 | 1.672E-07 | no |
| MEOX1 | 6.667 | 6.035 | 4.435 | 5.178 | 6.351 | 4.806 | 0.783 | -0.3538 | 0.01082 | 0.04351 | no |
| EPG5 | 3.55 | 3.533 | 2.706 | 2.819 | 3.542 | 2.763 | 0.783 | -0.353 | 0.0001635 | 0.001232 | no |
| CFAP73 | 4.918 | 5.441 | 3.771 | 3.598 | 5.179 | 3.684 | 0.783 | -0.3523 | 0.04538 | 0.1327 | no |
| TRMT5 | 16.895 | 16.46 | 13.023 | 12.144 | 16.678 | 12.584 | 0.784 | -0.3513 | 0.001104 | 0.006397 | no |
| SPO11 | 42.36 | 40.531 | 32.035 | 32.985 | 41.445 | 32.51 | 0.784 | -0.3511 | 4.875E-07 | 0.000006719 | no |
| MAGOH | 39.49 | 38.974 | 28.869 | 28.727 | 39.232 | 28.798 | 0.784 | -0.3519 | 0.0128 | 0.04956 | no |
| IL1RAP | 6.853 | 6.395 | 5.285 | 4.988 | 6.624 | 5.136 | 0.784 | -0.3511 | 0.001229 | 0.006984 | no |
| EAF1 | 21.772 | 19.224 | 16.921 | 15.193 | 20.498 | 16.057 | 0.784 | -0.3519 | 0.000004608 | 0.00005147 | no |
| DYNLL1 | 308.819 | 276.29 | 215.03 | 231.154 | 292.555 | 223.092 | 0.784 | -0.3502 | 5.447E-08 | 8.868E-07 | no |
| ALKBH1 | 24.715 | 23.779 | 18.836 | 19.731 | 24.247 | 19.284 | 0.784 | -0.3511 | 0.0003099 | 0.002147 | no |
| ZNF410 | 22.523 | 20.343 | 17.4 | 17.222 | 21.433 | 17.311 | 0.785 | -0.349 | 0.00001186 | 0.0001187 | no |
| WDR82 | 42.021 | 40.95 | 32.279 | 33.445 | 41.486 | 32.862 | 0.785 | -0.3487 | 3.55E-12 | 1.052E-10 | no |
| SMURF1 | 15.773 | 15.097 | 11.978 | 11.345 | 15.435 | 11.662 | 0.785 | -0.3485 | 0.000005373 | 0.00005904 | no |
| PRPF4B | 21.823 | 21.326 | 16.735 | 17.222 | 21.575 | 16.979 | 0.785 | -0.349 | 0.000000061 | 9.876E-07 | no |
| POC5 | 9.662 | 9.481 | 7.611 | 6.927 | 9.572 | 7.269 | 0.785 | -0.3492 | 0.01013 | 0.04116 | no |
| MRPL50 | 11.678 | 10.736 | 7.679 | 8.546 | 11.207 | 8.113 | 0.785 | -0.3497 | 0.03681 | 0.1134 | no |
| LRWD1 | 44.315 | 38.029 | 30.432 | 32.845 | 41.172 | 31.639 | 0.785 | -0.349 | 1.655E-07 | 0.000002505 | no |
| KANSL2 | 31.835 | 26.661 | 21.952 | 22.57 | 29.248 | 22.261 | 0.785 | -0.3498 | 0.0001292 | 0.0009972 | no |
| HIC2 | 2.109 | 2.229 | 1.895 | 1.359 | 2.169 | 1.627 | 0.785 | -0.3499 | 0.02194 | 0.07589 | no |
| FHL3 | 26.001 | 21.278 | 19.754 | 16.093 | 23.639 | 17.924 | 0.785 | -0.3491 | 0.003647 | 0.01754 | no |
| DHX30 | 33.306 | 29.474 | 24.58 | 24.869 | 31.39 | 24.724 | 0.785 | -0.3501 | 1.503E-08 | 2.695E-07 | no |
| CALHM2 | 24.797 | 20.392 | 18.093 | 19.411 | 22.595 | 18.752 | 0.785 | -0.349 | 0.00004209 | 0.000369 | no |
| ZMYND19 | 1.986 | 1.84 | 1.348 | 1.429 | 1.913 | 1.389 | 0.786 | -0.3481 | 0.04276 | 0.1267 | no |
| ZFPL1 | 12.717 | 11.807 | 9.662 | 9.476 | 12.262 | 9.569 | 0.786 | -0.3466 | 0.000541 | 0.003496 | no |
| YWHAH | 112.45 | 108.258 | 85.904 | 87.17 | 110.354 | 86.537 | 0.786 | -0.348 | 6.997E-09 | 1.343E-07 | no |
| UBE2Z | 47.937 | 43.899 | 35.913 | 36.973 | 45.918 | 36.443 | 0.786 | -0.347 | 1.751E-14 | 6.821E-13 | no |
| TGFBRAP1 | 16.411 | 16.995 | 12.779 | 14.163 | 16.703 | 13.471 | 0.786 | -0.3481 | 0.00000373 | 0.00004283 | no |
| SPSB1 | 2.099 | 1.986 | 1.563 | 1.329 | 2.042 | 1.446 | 0.786 | -0.3474 | 0.06048 | 0.1647 | no |
| SPIN1 | 21.988 | 22.368 | 17.966 | 17.032 | 22.178 | 17.499 | 0.786 | -0.3479 | 3.207E-07 | 0.000004578 | no |
| RNMT | 7.563 | 7.398 | 6.008 | 5.797 | 7.48 | 5.902 | 0.786 | -0.3465 | 0.00009683 | 0.0007761 | no |
| MYO1B | 31.475 | 30.311 | 24.59 | 24.409 | 30.893 | 24.499 | 0.786 | -0.3481 | 2.865E-11 | 7.582E-10 | no |
| KCTD11 | 22.904 | 18.903 | 14.996 | 14.473 | 20.904 | 14.735 | 0.786 | -0.3481 | 0.04169 | 0.1243 | no |
| HMBOX1 | 5.206 | 3.816 | 2.794 | 3.059 | 4.511 | 2.926 | 0.786 | -0.3465 | 0.03836 | 0.1171 | no |
| CPNE2 | 7.491 | 6.483 | 5.51 | 5.517 | 6.987 | 5.514 | 0.786 | -0.347 | 0.00007336 | 0.0006079 | no |
| CDC5L | 28.758 | 27.848 | 22.07 | 22.71 | 28.303 | 22.39 | 0.786 | -0.3472 | 6.752E-09 | 0.00000013 | no |
| PKNOX1 | 4.939 | 4.74 | 3.625 | 3.808 | 4.84 | 3.716 | 0.787 | -0.3464 | 0.001773 | 0.009526 | no |
| LYN | 22.183 | 21.064 | 16.511 | 17.172 | 21.623 | 16.841 | 0.787 | -0.346 | 3.259E-08 | 5.493E-07 | no |
| ISG20L2 | 20.517 | 20.411 | 15.27 | 16.762 | 20.464 | 16.016 | 0.787 | -0.345 | 0.0002738 | 0.001934 | no |
| FSTL3 | 51.754 | 46.887 | 39.225 | 36.813 | 49.32 | 38.019 | 0.787 | -0.3457 | 0.000009825 | 0.0001004 | no |
| ENOX1 | 7.192 | 6.677 | 5.305 | 5.477 | 6.934 | 5.391 | 0.787 | -0.3456 | 0.002157 | 0.0113 | no |
| ARID5A | 8.787 | 8.128 | 6.135 | 6.717 | 8.457 | 6.426 | 0.787 | -0.3456 | 0.01211 | 0.04741 | no |
| AK6 | 85.318 | 70.102 | 60.279 | 60.562 | 77.71 | 60.421 | 0.787 | -0.3457 | 0.000003818 | 0.00004375 | no |
| ZNF133 | 3.756 | 3.368 | 2.628 | 2.489 | 3.562 | 2.558 | 0.788 | -0.3445 | 0.04014 | 0.1209 | no |
| UBOX5 | 26.803 | 23.04 | 18.445 | 18.442 | 24.922 | 18.444 | 0.788 | -0.3443 | 0.00001388 | 0.0001359 | no |
| UBE4A | 8.478 | 8.478 | 6.673 | 6.737 | 8.478 | 6.705 | 0.788 | -0.3435 | 0.000006139 | 0.00006638 | no |
| PRKAB1 | 16.555 | 15.058 | 11.733 | 13.044 | 15.806 | 12.389 | 0.788 | -0.3438 | 0.0002905 | 0.002028 | no |
| DBR1 | 13.932 | 14.27 | 10.493 | 11.395 | 14.101 | 10.944 | 0.788 | -0.3446 | 0.002287 | 0.01186 | no |
| CDK20 | 7.151 | 7.252 | 5.422 | 5.677 | 7.201 | 5.55 | 0.788 | -0.3433 | 0.01623 | 0.05997 | no |
| ANKRD17 | 16.298 | 15.934 | 12.046 | 13.564 | 16.116 | 12.805 | 0.788 | -0.3441 | 1.065E-08 | 1.942E-07 | no |
| SYNJ1 | 5.896 | 5.928 | 4.601 | 4.708 | 5.912 | 4.655 | 0.789 | -0.3415 | 0.0005566 | 0.003571 | no |
| SRSF2 | 120.692 | 111.295 | 98.146 | 89.559 | 115.993 | 93.852 | 0.789 | -0.3424 | 2.056E-09 | 4.32E-08 | no |
| SLC51A | 8.694 | 7.952 | 6.35 | 6.407 | 8.323 | 6.378 | 0.789 | -0.3422 | 0.009611 | 0.03943 | no |
| RFC3 | 35.745 | 31.245 | 25.626 | 27.217 | 33.495 | 26.422 | 0.789 | -0.3426 | 0.0009851 | 0.00583 | no |
| PRNP | 103.807 | 96.733 | 80.795 | 78.134 | 100.27 | 79.465 | 0.789 | -0.3422 | 1.673E-12 | 5.115E-11 | no |
| NPAS1 | 7.563 | 6.911 | 6.038 | 5.497 | 7.237 | 5.768 | 0.789 | -0.3419 | 0.02656 | 0.08807 | no |
| LRRC8A | 9.672 | 9.412 | 7.601 | 7.377 | 9.542 | 7.489 | 0.789 | -0.3422 | 0.0004557 | 0.003005 | no |
| WDR77 | 51.425 | 43.704 | 36.724 | 38.172 | 47.564 | 37.448 | 0.79 | -0.3401 | 0.000005525 | 0.00006038 | no |
| TRRAP | 10.979 | 10.765 | 8.666 | 8.636 | 10.872 | 8.651 | 0.79 | -0.3392 | 3.409E-09 | 6.851E-08 | no |
| SYNRG | 23.511 | 23.215 | 18.523 | 18.881 | 23.363 | 18.702 | 0.79 | -0.3404 | 3.267E-10 | 7.662E-09 | no |
| SNX30 | 5.175 | 4.828 | 3.8 | 4.038 | 5.002 | 3.919 | 0.79 | -0.3406 | 0.0007419 | 0.004563 | no |
| MFN1 | 16.154 | 15.399 | 12.212 | 12.854 | 15.776 | 12.533 | 0.79 | -0.3396 | 2.879E-07 | 0.000004178 | no |
| KPNB1 | 161.169 | 154.794 | 125.745 | 127.022 | 157.982 | 126.383 | 0.79 | -0.3396 | 4.822E-35 | 6.227E-33 | no |
| ITPKC | 6.41 | 5.655 | 4.562 | 4.738 | 6.033 | 4.65 | 0.79 | -0.3407 | 0.009933 | 0.04053 | no |
| FBXO38 | 22.718 | 21.044 | 16.589 | 17.052 | 21.881 | 16.82 | 0.79 | -0.3399 | 8.621E-07 | 0.00001126 | no |
| DAB1 | 5.721 | 5.86 | 4.611 | 4.388 | 5.79 | 4.499 | 0.79 | -0.3398 | 0.002319 | 0.01199 | no |
| CWC22 | 8.056 | 7.154 | 5.745 | 6.197 | 7.605 | 5.971 | 0.79 | -0.3395 | 0.0009613 | 0.005708 | no |
| AGO3 | 1.677 | 1.518 | 1.172 | 1.119 | 1.598 | 1.145 | 0.79 | -0.3401 | 0.0603 | 0.1644 | no |
| WSB2 | 23.387 | 24.062 | 19.402 | 18.531 | 23.724 | 18.966 | 0.791 | -0.3377 | 0.00001681 | 0.0001617 | no |
| VASN | 34.829 | 34.652 | 26.974 | 28.037 | 34.74 | 27.505 | 0.791 | -0.3384 | 0.00002484 | 0.0002306 | no |
| TEX10 | 12.45 | 12.81 | 9.652 | 10.265 | 12.63 | 9.959 | 0.791 | -0.3389 | 0.0003957 | 0.002651 | no |
| RBM26 | 8.015 | 8.244 | 5.852 | 6.917 | 8.13 | 6.385 | 0.791 | -0.3383 | 0.001159 | 0.006667 | no |
| PAXIP1 | 17.924 | 16.771 | 14.019 | 13.084 | 17.348 | 13.552 | 0.791 | -0.3391 | 0.00006239 | 0.0005265 | no |
| LIPH | 7.573 | 7.524 | 5.852 | 6.107 | 7.549 | 5.979 | 0.791 | -0.3388 | 0.00007724 | 0.0006369 | no |
| CPSF7 | 33.244 | 31.712 | 25.958 | 25.128 | 32.478 | 25.543 | 0.791 | -0.3374 | 5.017E-08 | 8.238E-07 | no |
| TOX4 | 48.092 | 45.563 | 36.275 | 38.312 | 46.828 | 37.293 | 0.792 | -0.3368 | 4.265E-09 | 8.424E-08 | no |
| SLC7A1 | 25.857 | 24.899 | 19.901 | 20.72 | 25.378 | 20.31 | 0.792 | -0.3365 | 4.466E-11 | 1.146E-09 | no |
| NQO1 | 300.999 | 287.075 | 226.714 | 242.888 | 294.037 | 234.801 | 0.792 | -0.3363 | 1.543E-22 | 1.094E-20 | no |
| LRRC41 | 54.358 | 51.618 | 41.257 | 43.13 | 52.988 | 42.194 | 0.792 | -0.3362 | 1.734E-11 | 4.657E-10 | no |
| KLHL12 | 11.39 | 10.425 | 8.48 | 8.826 | 10.908 | 8.653 | 0.792 | -0.3369 | 0.00008206 | 0.0006703 | no |
| CCNB1IP1 | 2.665 | 2.443 | 1.739 | 1.819 | 2.554 | 1.779 | 0.792 | -0.3372 | 0.0722 | 0.1885 | no |
| CARM1 | 36.177 | 33.182 | 26.398 | 25.468 | 34.68 | 25.933 | 0.792 | -0.3369 | 0.00006919 | 0.0005784 | no |
| ZNF207 | 118.253 | 114.03 | 90.994 | 92.198 | 116.142 | 91.596 | 0.793 | -0.3351 | 1.809E-15 | 7.877E-14 | no |
| WDR1 | 272.724 | 239.468 | 201.538 | 207.764 | 256.096 | 204.651 | 0.793 | -0.3344 | 2.312E-17 | 1.186E-15 | no |
| TSTD2 | 11.195 | 11.865 | 8.04 | 9.726 | 11.53 | 8.883 | 0.793 | -0.3347 | 0.01427 | 0.0542 | no |
| TRMT61B | 13.458 | 12.099 | 10.219 | 10.035 | 12.779 | 10.127 | 0.793 | -0.3342 | 0.00138 | 0.007708 | no |
| THAP4 | 21.072 | 17.112 | 14.498 | 15.363 | 19.092 | 14.93 | 0.793 | -0.3355 | 0.001398 | 0.007787 | no |
| POLG | 26.423 | 24.47 | 20.438 | 20.081 | 25.447 | 20.259 | 0.793 | -0.3344 | 1.818E-07 | 0.000002729 | no |
| PEX10 | 6.914 | 5.052 | 4.494 | 4.578 | 5.983 | 4.536 | 0.793 | -0.335 | 0.02697 | 0.08907 | no |
| GTF3C1 | 27.678 | 27.478 | 21.054 | 23.129 | 27.578 | 22.091 | 0.793 | -0.3349 | 3.585E-10 | 8.295E-09 | no |
| EPS15L1 | 17.769 | 17.482 | 13.814 | 13.274 | 17.625 | 13.544 | 0.793 | -0.3343 | 0.000006365 | 0.0000685 | no |
| DPF2 | 52.217 | 46.128 | 39.606 | 38.472 | 49.172 | 39.039 | 0.793 | -0.3338 | 5.136E-07 | 0.000007069 | no |
| TMEM186 | 4.465 | 4.137 | 3.048 | 2.929 | 4.301 | 2.989 | 0.794 | -0.3328 | 0.04537 | 0.1327 | no |
| RABL6 | 44.305 | 36.842 | 32.24 | 31.945 | 40.573 | 32.093 | 0.794 | -0.3327 | 0.000004516 | 0.00005059 | no |
| OGT | 17.121 | 16.314 | 13.257 | 13.434 | 16.718 | 13.345 | 0.794 | -0.3336 | 3.745E-07 | 0.000005224 | no |
| AHCTF1 | 16.957 | 16.382 | 12.818 | 13.914 | 16.669 | 13.366 | 0.794 | -0.3322 | 1.15E-08 | 2.089E-07 | no |
| ACTR3 | 48.4 | 44.95 | 36.724 | 38.053 | 46.675 | 37.388 | 0.794 | -0.3335 | 4.463E-12 | 1.309E-10 | no |
| ZSCAN23 | 3.21 | 3.378 | 2.833 | 2.239 | 3.294 | 2.536 | 0.795 | -0.3312 | 0.02869 | 0.09349 | no |
| YTHDF1 | 41.681 | 37.562 | 32.504 | 31.426 | 39.621 | 31.965 | 0.795 | -0.331 | 0.000001133 | 0.00001447 | no |
| PPIG | 11.051 | 9.996 | 8.158 | 8.656 | 10.524 | 8.407 | 0.795 | -0.3303 | 0.00001425 | 0.0001392 | no |
| PHF2 | 18.809 | 17.9 | 12.701 | 13.194 | 18.355 | 12.948 | 0.795 | -0.331 | 3.211E-07 | 0.000004578 | no |
| NDUFAF6 | 4.599 | 6.463 | 3.39 | 5.048 | 5.531 | 4.219 | 0.795 | -0.3308 | 0.08161 | 0.2062 | no |
| MRPS10 | 68.834 | 61.41 | 48.682 | 55.045 | 65.122 | 51.864 | 0.795 | -0.3317 | 3.271E-07 | 0.000004641 | no |
| EME1 | 9.487 | 9.159 | 7.083 | 7.387 | 9.323 | 7.235 | 0.795 | -0.3309 | 0.01068 | 0.04303 | no |
| CALD1 | 108.9 | 107.304 | 85.836 | 88.259 | 108.102 | 87.047 | 0.795 | -0.3306 | 1.443E-19 | 8.732E-18 | no |
| WTIP | 6.811 | 6.502 | 4.015 | 4.888 | 6.656 | 4.451 | 0.796 | -0.3298 | 0.05296 | 0.1497 | no |
| STK40 | 9.878 | 8.507 | 7.093 | 7.367 | 9.192 | 7.23 | 0.796 | -0.3296 | 0.002628 | 0.01328 | no |
| SART3 | 10.022 | 9.753 | 7.982 | 7.846 | 9.887 | 7.914 | 0.796 | -0.3285 | 0.000005452 | 0.0000598 | no |
| RNF14 | 19.498 | 18.708 | 15.202 | 15.013 | 19.103 | 15.107 | 0.796 | -0.3294 | 0.00003299 | 0.0002979 | no |
| RELA | 31.114 | 27.498 | 22.626 | 23.699 | 29.306 | 23.163 | 0.796 | -0.329 | 0.00002832 | 0.0002591 | no |
| MSANTD2 | 11.544 | 10.542 | 7.708 | 8.686 | 11.043 | 8.197 | 0.796 | -0.3284 | 0.002576 | 0.01308 | no |
| C3AR1 | 13.407 | 11.252 | 9.34 | 9.496 | 12.329 | 9.418 | 0.796 | -0.3292 | 0.001219 | 0.006945 | no |
| AZI2 | 12.254 | 11.223 | 9.262 | 9.356 | 11.739 | 9.309 | 0.796 | -0.3293 | 0.0007429 | 0.004564 | no |
| AMIGO3 | 9.929 | 8.595 | 6.653 | 7.347 | 9.262 | 7 | 0.796 | -0.3292 | 0.0338 | 0.1061 | no |
| TUBAL3 | 4.877 | 3.806 | 2.97 | 2.779 | 4.341 | 2.875 | 0.797 | -0.3276 | 0.05462 | 0.1531 | no |
| TADA2A | 15.084 | 14.844 | 10.747 | 12.584 | 14.964 | 11.665 | 0.797 | -0.3265 | 0.002481 | 0.01267 | no |
| NFYA | 21.484 | 19.866 | 16.892 | 16.103 | 20.675 | 16.498 | 0.797 | -0.3281 | 0.00002081 | 0.0001958 | no |
| ME2 | 10.649 | 9.588 | 7.923 | 8.206 | 10.118 | 8.064 | 0.797 | -0.327 | 0.000108 | 0.0008547 | no |
| LIF | 3.519 | 2.94 | 2.638 | 2.249 | 3.229 | 2.444 | 0.797 | -0.3269 | 0.03278 | 0.1037 | no |
| GFPT2 | 67.662 | 59.706 | 50.529 | 51.436 | 63.684 | 50.983 | 0.797 | -0.328 | 2.344E-09 | 4.862E-08 | no |
| F2RL2 | 67.692 | 65.887 | 52.141 | 54.765 | 66.79 | 53.453 | 0.797 | -0.3273 | 8.993E-09 | 1.692E-07 | no |
| ZNF473 | 3.025 | 2.648 | 2.306 | 2.089 | 2.837 | 2.197 | 0.798 | -0.326 | 0.02227 | 0.07676 | no |
| ZEB2 | 4.085 | 3.903 | 2.872 | 3.209 | 3.994 | 3.04 | 0.798 | -0.3262 | 0.002085 | 0.01097 | no |
| SSFA2 | 19.148 | 19.088 | 15.104 | 15.603 | 19.118 | 15.354 | 0.798 | -0.3252 | 0.000001927 | 0.00002377 | no |
| PMPCA | 16.761 | 15.72 | 12.32 | 13.644 | 16.241 | 12.982 | 0.798 | -0.3264 | 0.00009704 | 0.0007773 | no |
| NFX1 | 13.962 | 11.914 | 10.219 | 10.355 | 12.938 | 10.287 | 0.798 | -0.3255 | 0.0004909 | 0.003209 | no |
| ELP3 | 12.337 | 12.138 | 9.584 | 9.915 | 12.238 | 9.749 | 0.798 | -0.3264 | 0.0003043 | 0.002115 | no |
| ATG16L1 | 14.374 | 13.092 | 10.59 | 11.175 | 13.733 | 10.883 | 0.798 | -0.326 | 0.0007499 | 0.004595 | no |
| AGO2 | 15.218 | 15.019 | 13.883 | 16.303 | 15.119 | 15.093 | 0.798 | -0.3264 | 1.32E-10 | 3.229E-09 | no |
| ZC3H13 | 9.939 | 10.201 | 8.206 | 7.846 | 10.07 | 8.026 | 0.799 | -0.3245 | 0.00003328 | 0.0003 | no |
| TRAF3 | 8.396 | 7.943 | 6.555 | 6.547 | 8.169 | 6.551 | 0.799 | -0.3234 | 0.00006338 | 0.0005337 | no |
| NKAIN1 | 10.937 | 8.955 | 8.539 | 7.217 | 9.946 | 7.878 | 0.799 | -0.324 | 0.009444 | 0.03892 | no |
| MYBBP1A | 94.29 | 83.223 | 71.338 | 71.607 | 88.757 | 71.472 | 0.799 | -0.3241 | 2.464E-13 | 8.428E-12 | no |
| MED27 | 9.651 | 7.69 | 7.396 | 6.277 | 8.671 | 6.837 | 0.799 | -0.3229 | 0.004115 | 0.01935 | no |
| MED22 | 16.061 | 14.961 | 12.554 | 12.274 | 15.511 | 12.414 | 0.799 | -0.3241 | 0.0001259 | 0.0009745 | no |
| HNRNPU | 181.12 | 171.429 | 140.507 | 144.074 | 176.274 | 142.291 | 0.799 | -0.3234 | 6.551E-25 | 5.303E-23 | no |
| CDCA7 | 11.833 | 12.167 | 9.301 | 9.606 | 12 | 9.454 | 0.799 | -0.3237 | 0.005229 | 0.02363 | no |
| WDR3 | 21.309 | 19.107 | 16.169 | 16.412 | 20.208 | 16.291 | 0.8 | -0.3219 | 0.000007273 | 0.0000769 | no |
| RADIL | 2.469 | 2.287 | 1.759 | 1.909 | 2.378 | 1.834 | 0.8 | -0.3224 | 0.02749 | 0.09039 | no |
| PEAK1 | 11.225 | 11.68 | 9.066 | 8.926 | 11.453 | 8.996 | 0.8 | -0.3228 | 0.00000112 | 0.00001434 | no |
| MAPK1IP1L | 27.77 | 25.093 | 21.19 | 21.35 | 26.431 | 21.27 | 0.8 | -0.3226 | 1.497E-08 | 2.688E-07 | no |
| GRB10 | 10.896 | 11.145 | 8.949 | 8.596 | 11.021 | 8.773 | 0.8 | -0.3214 | 0.000262 | 0.001863 | no |
| GLOD4 | 30.024 | 29.152 | 23.125 | 24.369 | 29.588 | 23.747 | 0.8 | -0.3219 | 7.41E-08 | 0.000001181 | no |
| FAM120B | 10.207 | 10.688 | 8.402 | 8.226 | 10.448 | 8.314 | 0.8 | -0.322 | 0.0005564 | 0.003571 | no |
| EIF2S1 | 93.467 | 89.248 | 73.487 | 73.996 | 91.358 | 73.742 | 0.8 | -0.3226 | 1.63E-16 | 7.71E-15 | no |
| CCNF | 17.718 | 15.983 | 13.502 | 13.504 | 16.851 | 13.503 | 0.8 | -0.3215 | 0.00001325 | 0.0001306 | no |
| ACER2 | 4.867 | 5.139 | 4.025 | 3.888 | 5.003 | 3.957 | 0.8 | -0.3222 | 0.004956 | 0.02254 | no |
| PAFAH1B2 | 26.474 | 24.539 | 20.213 | 20.401 | 25.507 | 20.307 | 0.801 | -0.3207 | 1.344E-08 | 2.426E-07 | no |
| EIF3A | 146.116 | 144.068 | 117.294 | 117.746 | 145.092 | 117.52 | 0.801 | -0.3206 | 4.417E-24 | 3.43E-22 | no |
| CCDC71 | 14.25 | 14.308 | 10.61 | 11.375 | 14.279 | 10.992 | 0.801 | -0.3209 | 0.01102 | 0.04416 | no |
| ATG4B | 23.254 | 20.519 | 17.859 | 17.022 | 21.886 | 17.441 | 0.801 | -0.3197 | 0.0000746 | 0.0006169 | no |
| SLC7A6OS | 19.261 | 20.869 | 15.592 | 16.402 | 20.065 | 15.997 | 0.802 | -0.3186 | 0.001277 | 0.007221 | no |
| RBM10 | 51.919 | 48.766 | 41.286 | 39.652 | 50.343 | 40.469 | 0.802 | -0.3175 | 1.221E-08 | 2.213E-07 | no |
| PHF20L1 | 24.571 | 24.266 | 18.855 | 19.791 | 24.419 | 19.323 | 0.802 | -0.3186 | 9.625E-08 | 0.000001507 | no |
| MANF | 25.147 | 23.614 | 17.839 | 19.711 | 24.38 | 18.775 | 0.802 | -0.318 | 0.00002494 | 0.0002314 | no |
| GRK2 | 32.401 | 30.865 | 25.811 | 26.718 | 31.633 | 26.264 | 0.802 | -0.3179 | 0.00002122 | 0.0001992 | no |
| GMPS | 12.193 | 11.953 | 9.359 | 10.105 | 12.073 | 9.732 | 0.802 | -0.3187 | 0.00001764 | 0.0001686 | no |
| GATAD2B | 5.402 | 6.668 | 4.904 | 3.239 | 6.035 | 4.072 | 0.802 | -0.3185 | 0.09991 | 0.2377 | no |
| DHX38 | 18.13 | 17.696 | 14.938 | 13.894 | 17.913 | 14.416 | 0.802 | -0.3176 | 0.00003822 | 0.00034 | no |
| CSK | 55.983 | 50.927 | 42.381 | 43.65 | 53.455 | 43.016 | 0.802 | -0.3175 | 3.198E-07 | 0.000004576 | no |
| CENPO | 5.464 | 5.023 | 4.328 | 3.918 | 5.244 | 4.123 | 0.802 | -0.3182 | 0.01189 | 0.04678 | no |
| C18orf54 | 6.894 | 7.067 | 4.856 | 5.847 | 6.981 | 5.351 | 0.802 | -0.318 | 0.02124 | 0.07414 | no |
| USP10 | 43.801 | 42.118 | 34.77 | 34.254 | 42.96 | 34.512 | 0.803 | -0.3164 | 4.738E-08 | 7.802E-07 | no |
| SBNO2 | 22.77 | 22.679 | 18.132 | 17.452 | 22.724 | 17.792 | 0.803 | -0.3174 | 0.00007548 | 0.0006228 | no |
| RPP38 | 23.295 | 19.088 | 14.254 | 18.232 | 21.192 | 16.243 | 0.803 | -0.3157 | 0.01905 | 0.06803 | no |
| MYADM | 134.263 | 119.432 | 95.547 | 97.195 | 126.847 | 96.371 | 0.803 | -0.3162 | 5.67E-13 | 1.846E-11 | no |
| LARP4B | 5.988 | 6.599 | 4.533 | 5.038 | 6.293 | 4.786 | 0.803 | -0.3163 | 0.01117 | 0.04459 | no |
| IQCJ-SCHIP1 | 27.544 | 24.441 | 21.61 | 19.941 | 25.992 | 20.776 | 0.803 | -0.3163 | 0.0003407 | 0.002326 | no |
| GSR | 41.908 | 41.154 | 32.65 | 34.164 | 41.531 | 33.407 | 0.803 | -0.3166 | 0.000005458 | 0.0000598 | no |
| GATAD2A | 18.757 | 17.521 | 14.098 | 15.333 | 18.139 | 14.716 | 0.803 | -0.3165 | 0.000001437 | 0.00001801 | no |
| FAM193A | 8.53 | 8.653 | 6.829 | 6.927 | 8.591 | 6.878 | 0.803 | -0.3158 | 0.001059 | 0.006173 | no |
| ACSS3 | 9.487 | 8.916 | 7.337 | 7.437 | 9.201 | 7.387 | 0.803 | -0.3157 | 0.0004988 | 0.003256 | no |
| ZZZ3 | 15.969 | 14.815 | 12.417 | 12.334 | 15.392 | 12.375 | 0.804 | -0.3142 | 0.00003499 | 0.0003137 | no |
| UBAP2L | 95.216 | 91.779 | 73.302 | 75.485 | 93.498 | 74.394 | 0.804 | -0.3143 | 8.051E-16 | 3.643E-14 | no |
| PWP1 | 21.597 | 19.944 | 17.409 | 16.183 | 20.77 | 16.796 | 0.804 | -0.315 | 6.519E-07 | 0.000008743 | no |
| PPP2CA | 50.54 | 49.749 | 40.212 | 41.091 | 50.145 | 40.651 | 0.804 | -0.3142 | 8.172E-10 | 1.836E-08 | no |
| PHYKPL | 0.792 | 1.149 | 0.742 | 0.64 | 0.971 | 0.691 | 0.804 | -0.3144 | 0.09875 | 0.2357 | no |
| METTL13 | 5.628 | 4.935 | 4.308 | 4.098 | 5.281 | 4.203 | 0.804 | -0.3145 | 0.004862 | 0.02217 | no |
| KPNA6 | 38.018 | 36.044 | 29.7 | 30.496 | 37.031 | 30.098 | 0.804 | -0.3143 | 1.03E-12 | 3.234E-11 | no |
| HOMER1 | 33.028 | 7.067 | 21.347 | 5.757 | 20.047 | 13.552 | 0.804 | -0.3151 | 0.0009281 | 0.005536 | no |
| HEATR4 | 17.759 | 14.474 | 14.107 | 12.914 | 16.117 | 13.511 | 0.804 | -0.3156 | 0.001589 | 0.008708 | no |
| EIF2B3 | 25.96 | 24.616 | 19.842 | 20.431 | 25.288 | 20.136 | 0.804 | -0.314 | 0.002003 | 0.01059 | no |
| ATAD1 | 30.127 | 28.442 | 22.304 | 24.959 | 29.285 | 23.631 | 0.804 | -0.3144 | 0.00001644 | 0.0001584 | no |
| XBP1 | 76.623 | 68.36 | 57.27 | 58.983 | 72.492 | 58.127 | 0.805 | -0.3136 | 0.000003633 | 0.00004205 | no |
| SUPV3L1 | 58.01 | 48.999 | 42.039 | 38.932 | 53.505 | 40.486 | 0.805 | -0.3131 | 0.001118 | 0.006466 | no |
| PSMG3 | 63.196 | 58.295 | 50.577 | 48.658 | 60.745 | 49.617 | 0.805 | -0.3123 | 0.000127 | 0.0009824 | no |
| PHAX | 23.428 | 20.908 | 17.312 | 18.132 | 22.168 | 17.722 | 0.805 | -0.3133 | 0.001202 | 0.006875 | no |
| DHX37 | 33.399 | 31.352 | 28.166 | 28.117 | 32.376 | 28.142 | 0.805 | -0.3127 | 0.00003953 | 0.0003506 | no |
| CAB39 | 21.463 | 20.178 | 16.56 | 16.832 | 20.821 | 16.696 | 0.805 | -0.3137 | 0.00004131 | 0.0003643 | no |
| BIN3 | 10.094 | 8.936 | 7.728 | 7.047 | 9.515 | 7.387 | 0.805 | -0.3135 | 0.02672 | 0.08852 | no |
| ZNF592 | 8.293 | 7.641 | 6.653 | 6.137 | 7.967 | 6.395 | 0.806 | -0.3107 | 0.0006601 | 0.004137 | no |
| XAB2 | 22.986 | 18.348 | 15.68 | 17.402 | 20.667 | 16.541 | 0.806 | -0.3107 | 0.001696 | 0.009181 | no |
| PAX3 | 17.749 | 15.165 | 13.541 | 12.914 | 16.457 | 13.227 | 0.806 | -0.3114 | 0.0005936 | 0.003761 | no |
| CYTH3 | 26.484 | 23.371 | 19.852 | 20.69 | 24.928 | 20.271 | 0.806 | -0.3104 | 0.000007363 | 0.00007772 | no |
| CRLF1 | 22.986 | 17.686 | 17.781 | 15.763 | 20.336 | 16.772 | 0.806 | -0.3112 | 0.01061 | 0.04282 | no |
| VASP | 45.941 | 43.091 | 36.626 | 35.594 | 44.516 | 36.11 | 0.807 | -0.3089 | 2.388E-07 | 0.000003522 | no |
| SP6 | 6.595 | 7.203 | 4.816 | 5.198 | 6.899 | 5.007 | 0.807 | -0.3091 | 0.09129 | 0.2232 | no |
| PRICKLE3 | 11.493 | 9.422 | 8.148 | 8.316 | 10.457 | 8.232 | 0.807 | -0.3094 | 0.01735 | 0.06325 | no |
| NECTIN4 | 12.964 | 11.943 | 10.141 | 9.975 | 12.454 | 10.058 | 0.807 | -0.3089 | 0.001873 | 0.009961 | no |
| MFSD5 | 46.414 | 40.92 | 33.695 | 35.943 | 43.667 | 34.819 | 0.807 | -0.31 | 0.0007757 | 0.004732 | no |
| MCM3AP | 22.451 | 21.55 | 18.074 | 17.852 | 22.001 | 17.963 | 0.807 | -0.3101 | 3.286E-08 | 5.531E-07 | no |
| KIAA1024 | 6.986 | 6.405 | 5.305 | 5.258 | 6.696 | 5.281 | 0.807 | -0.3092 | 0.00153 | 0.008431 | no |
| ANKRD39 | 6.657 | 6.025 | 5.373 | 3.928 | 6.341 | 4.651 | 0.807 | -0.3101 | 0.08348 | 0.2098 | no |
| ANKLE2 | 20.321 | 19.448 | 16.56 | 15.693 | 19.885 | 16.127 | 0.807 | -0.309 | 0.000006792 | 0.00007229 | no |
| ZBTB21 | 4.116 | 4.108 | 3.204 | 3.408 | 4.112 | 3.306 | 0.808 | -0.308 | 0.003794 | 0.01816 | no |
| UBE3A | 11.678 | 12.644 | 10.121 | 9.726 | 12.161 | 9.924 | 0.808 | -0.3083 | 0.000012 | 0.0001197 | no |
| MON1B | 7.635 | 7.018 | 6.077 | 5.797 | 7.326 | 5.937 | 0.808 | -0.3083 | 0.0007093 | 0.004402 | no |
| KIAA1958 | 4.301 | 3.134 | 3.82 | 2.659 | 3.718 | 3.239 | 0.808 | -0.3071 | 0.04669 | 0.1358 | no |
| INTS7 | 9.734 | 9.636 | 7.523 | 8.086 | 9.685 | 7.804 | 0.808 | -0.3077 | 0.001536 | 0.008455 | no |
| GCLC | 10.896 | 9.461 | 7.962 | 8.346 | 10.178 | 8.154 | 0.808 | -0.3075 | 0.003981 | 0.01885 | no |
| UNC5A | 3.293 | 3.29 | 2.745 | 2.339 | 3.292 | 2.542 | 0.809 | -0.3061 | 0.05406 | 0.1519 | no |
| SPRED3 | 13.16 | 11.7 | 9.584 | 10.455 | 12.43 | 10.02 | 0.809 | -0.3055 | 0.001006 | 0.005926 | no |
| SIPA1 | 19.323 | 18.27 | 15.856 | 14.533 | 18.797 | 15.194 | 0.809 | -0.3054 | 0.0002141 | 0.001566 | no |
| MBNL1 | 36.352 | 34.438 | 29.055 | 28.897 | 35.395 | 28.976 | 0.809 | -0.306 | 4.461E-11 | 1.146E-09 | no |
| GFM2 | 8.571 | 8.585 | 6.604 | 7.167 | 8.578 | 6.886 | 0.809 | -0.3065 | 0.004385 | 0.02041 | no |
| FIP1L1 | 21.34 | 20.392 | 16.12 | 17.922 | 20.866 | 17.021 | 0.809 | -0.3063 | 0.000004387 | 0.00004929 | no |
| EARS2 | 2.778 | 3.095 | 2.452 | 2.179 | 2.937 | 2.316 | 0.809 | -0.3049 | 0.02986 | 0.0964 | no |
| CSRP1 | 187.479 | 167.954 | 146.036 | 143.234 | 177.716 | 144.635 | 0.809 | -0.3061 | 1.563E-11 | 4.248E-10 | no |
| USF1 | 88.363 | 82.211 | 69.599 | 67.959 | 85.287 | 68.779 | 0.81 | -0.3033 | 8.014E-08 | 0.000001272 | no |
| TAF11 | 23.027 | 22.495 | 18.24 | 17.702 | 22.761 | 17.971 | 0.81 | -0.3045 | 0.01279 | 0.04956 | no |
| SLC16A6 | 4.599 | 4.254 | 3.048 | 3.638 | 4.426 | 3.343 | 0.81 | -0.3035 | 0.03567 | 0.1106 | no |
| RNF4 | 39.284 | 35.382 | 30.276 | 30.616 | 37.333 | 30.446 | 0.81 | -0.3046 | 0.000003535 | 0.00004108 | no |
| RABEP1 | 25.99 | 74.91 | 30.149 | 26.128 | 50.45 | 28.139 | 0.81 | -0.3042 | 0.0001085 | 0.000857 | no |
| LRRC59 | 197.408 | 181.144 | 154.048 | 155.189 | 189.276 | 154.618 | 0.81 | -0.3042 | 3.898E-18 | 2.114E-16 | no |
| EXOSC5 | 4.671 | 3.893 | 3.693 | 2.859 | 4.282 | 3.276 | 0.81 | -0.3038 | 0.06587 | 0.1754 | no |
| ANAPC2 | 23.799 | 21.463 | 18.152 | 18.432 | 22.631 | 18.292 | 0.81 | -0.3041 | 0.0003529 | 0.002404 | no |
| UBIAD1 | 13.119 | 12.956 | 10.571 | 10.465 | 13.037 | 10.518 | 0.811 | -0.3027 | 0.002073 | 0.0109 | no |
| TIMM10 | 15.547 | 14.523 | 11.684 | 12.404 | 15.035 | 12.044 | 0.811 | -0.3018 | 0.006868 | 0.02979 | no |
| TEX30 | 9.713 | 6.726 | 6.477 | 6.077 | 8.22 | 6.277 | 0.811 | -0.3027 | 0.06245 | 0.1685 | no |
| SMYD5 | 26.752 | 24.071 | 20.165 | 21.3 | 25.412 | 20.733 | 0.811 | -0.302 | 0.00007171 | 0.0005968 | no |
| PRPF40A | 30.538 | 29.853 | 24.395 | 24.839 | 30.196 | 24.617 | 0.811 | -0.3031 | 5.175E-11 | 1.316E-09 | no |
| PDGFB | 17.348 | 15.457 | 13.873 | 12.474 | 16.402 | 13.174 | 0.811 | -0.3028 | 0.0005905 | 0.003744 | no |
| NRF1 | 7.851 | 6.784 | 5.852 | 5.847 | 7.317 | 5.85 | 0.811 | -0.302 | 0.008625 | 0.03611 | no |
| LCMT2 | 2.037 | 2.492 | 1.719 | 1.449 | 2.264 | 1.584 | 0.811 | -0.3021 | 0.1162 | 0.2657 | no |
| KIAA0232 | 17.852 | 16.304 | 13.502 | 14.373 | 17.078 | 13.938 | 0.811 | -0.3026 | 0.00000145 | 0.00001815 | no |
| ID2 | 7.902 | 7.096 | 6.096 | 5.697 | 7.499 | 5.896 | 0.811 | -0.3029 | 0.02924 | 0.09484 | no |
| DYSF | 2.233 | 2.151 | 1.768 | 1.679 | 2.192 | 1.724 | 0.811 | -0.3022 | 0.03678 | 0.1134 | no |
| TP53I11 | 28.624 | 28.237 | 22.89 | 23.469 | 28.43 | 23.18 | 0.812 | -0.3008 | 0.000009616 | 0.00009868 | no |
| TAF3 | 3.262 | 2.92 | 2.638 | 2.539 | 3.091 | 2.588 | 0.812 | -0.3007 | 0.04005 | 0.1208 | no |
| PMM2 | 12.45 | 11.758 | 9.564 | 10.075 | 12.104 | 9.819 | 0.812 | -0.2997 | 0.001002 | 0.005916 | no |
| PDXK | 14.96 | 12.917 | 11.206 | 11.425 | 13.939 | 11.316 | 0.812 | -0.3007 | 0.0005297 | 0.003426 | no |
| LSM12 | 41.969 | 38.506 | 33.148 | 32.475 | 40.237 | 32.812 | 0.812 | -0.3011 | 0.000005999 | 0.00006511 | no |
| LEO1 | 38.636 | 38.769 | 31.155 | 31.875 | 38.703 | 31.515 | 0.812 | -0.2997 | 0.00002371 | 0.0002208 | no |
| LANCL2 | 11.503 | 10.853 | 9.232 | 8.896 | 11.178 | 9.064 | 0.812 | -0.3013 | 0.0008091 | 0.004908 | no |
| EIF1AD | 5.844 | 6.142 | 4.768 | 4.878 | 5.993 | 4.823 | 0.812 | -0.3002 | 0.001589 | 0.008707 | no |
| ZNF580 | 41.321 | 41.426 | 41.492 | 41.021 | 41.373 | 41.257 | 0.813 | -0.2985 | 0.01166 | 0.04617 | no |
| ZNF550 | 5.381 | 4.468 | 4.054 | 3.888 | 4.925 | 3.971 | 0.813 | -0.2978 | 0.03729 | 0.1147 | no |
| SS18L1 | 2.84 | 2.511 | 2.032 | 2.149 | 2.675 | 2.091 | 0.813 | -0.299 | 0.05275 | 0.1492 | no |
| PARS2 | 4.177 | 4.39 | 3.273 | 3.598 | 4.284 | 3.436 | 0.813 | -0.2987 | 0.01738 | 0.06331 | no |
| NUBP1 | 25.455 | 24.597 | 19.959 | 20.461 | 25.026 | 20.21 | 0.813 | -0.2995 | 0.0002616 | 0.001862 | no |
| MRM3 | 7.542 | 6.444 | 5.451 | 5.727 | 6.993 | 5.589 | 0.813 | -0.2989 | 0.02251 | 0.07745 | no |
| LYRM2 | 7.274 | 6.522 | 5.315 | 6.047 | 6.898 | 5.681 | 0.813 | -0.2987 | 0.02732 | 0.08995 | no |
| LPCAT2 | 31.732 | 33.922 | 30.657 | 33.055 | 32.827 | 31.856 | 0.813 | -0.2978 | 0.001776 | 0.009536 | no |
| GGA3 | 18.109 | 17.044 | 14.43 | 14.293 | 17.577 | 14.361 | 0.813 | -0.2991 | 0.00001104 | 0.0001114 | no |
| DPP9 | 27.75 | 24.646 | 21.425 | 21.89 | 26.198 | 21.657 | 0.813 | -0.2979 | 0.00005219 | 0.000448 | no |
| DPH2 | 15.495 | 13.812 | 11.567 | 12.124 | 14.653 | 11.846 | 0.813 | -0.2994 | 0.002305 | 0.01194 | no |
| DGKH | 1.873 | 1.324 | 1.026 | 1.03 | 1.599 | 1.028 | 0.813 | -0.2982 | 0.1239 | 0.2777 | no |
| CUTC | 7.408 | 7.222 | 5.5 | 5.917 | 7.315 | 5.708 | 0.813 | -0.2989 | 0.06768 | 0.1793 | no |
| ZNF800 | 10.649 | 9.87 | 9.047 | 10.275 | 10.259 | 9.661 | 0.814 | -0.2971 | 0.0249 | 0.08377 | no |
| WAPL | 26.67 | 26.816 | 22.666 | 21.25 | 26.743 | 21.958 | 0.814 | -0.2963 | 3.045E-07 | 0.000004387 | no |
| UGCG | 39.85 | 36.53 | 31.8 | 30.096 | 38.19 | 30.948 | 0.814 | -0.2973 | 8.922E-07 | 0.00001163 | no |
| TRNAU1AP | 16.031 | 15.486 | 12.466 | 12.864 | 15.758 | 12.665 | 0.814 | -0.2962 | 0.004484 | 0.02076 | no |
| SLC45A4 | 10.732 | 9.004 | 7.962 | 8.996 | 9.868 | 8.479 | 0.814 | -0.2969 | 0.002558 | 0.01302 | no |
| SLC37A3 | 17.142 | 16.051 | 13.677 | 12.904 | 16.596 | 13.29 | 0.814 | -0.2977 | 0.001045 | 0.006111 | no |
| SLC35F2 | 78.671 | 75.631 | 61.979 | 63.241 | 77.151 | 62.61 | 0.814 | -0.2975 | 8.602E-07 | 0.00001126 | no |
| RPS6KB1 | 21.113 | 19.944 | 17.048 | 16.582 | 20.529 | 16.815 | 0.814 | -0.2963 | 0.000006503 | 0.00006966 | no |
| RHBDD3 | 7.223 | 5.976 | 5.403 | 4.638 | 6.599 | 5.021 | 0.814 | -0.2968 | 0.07363 | 0.1913 | no |
| RAB2B | 30.116 | 27.546 | 23.936 | 23.209 | 28.831 | 23.572 | 0.814 | -0.2972 | 0.0000714 | 0.0005952 | no |
| NCS1 | 13.448 | 12.732 | 10.248 | 11.265 | 13.09 | 10.756 | 0.814 | -0.2969 | 0.0005032 | 0.003279 | no |
| FARP2 | 6.071 | 5.714 | 4.895 | 4.718 | 5.893 | 4.806 | 0.814 | -0.2968 | 0.001782 | 0.009553 | no |
| E4F1 | 8.91 | 8.789 | 6.927 | 7.137 | 8.849 | 7.032 | 0.814 | -0.2961 | 0.005852 | 0.02604 | no |
| DET1 | 33.399 | 28.919 | 25.509 | 24.959 | 31.159 | 25.234 | 0.814 | -0.2971 | 0.0005489 | 0.003535 | no |
| C6orf62 | 44.048 | 42.303 | 34.995 | 35.264 | 43.175 | 35.13 | 0.814 | -0.2972 | 5.122E-08 | 8.398E-07 | no |
| SKIV2L2 | 47.814 | 46.936 | 38.766 | 39.222 | 47.375 | 38.994 | 0.815 | -0.2947 | 1.41E-09 | 3.053E-08 | no |
| RBM17 | 13.952 | 12.323 | 10.209 | 10.645 | 13.137 | 10.427 | 0.815 | -0.2943 | 0.003886 | 0.01853 | no |
| RAB35 | 27.431 | 26.845 | 20.243 | 20.271 | 27.138 | 20.257 | 0.815 | -0.2955 | 0.00008073 | 0.0006609 | no |
| PAFAH1B1 | 40.2 | 39.003 | 31.566 | 33.545 | 39.602 | 32.556 | 0.815 | -0.2946 | 2.635E-09 | 5.427E-08 | no |
| MED30 | 39.737 | 36.745 | 29.221 | 29.566 | 38.241 | 29.393 | 0.815 | -0.2949 | 0.01404 | 0.05359 | no |
| CYLD | 15.825 | 15.068 | 13.228 | 12.184 | 15.447 | 12.706 | 0.815 | -0.2949 | 0.00003146 | 0.0002859 | no |
| COPS2 | 40.231 | 38.088 | 31.028 | 33.335 | 39.16 | 32.181 | 0.815 | -0.2957 | 2.902E-08 | 4.949E-07 | no |
| ANKIB1 | 9.384 | 10.045 | 7.865 | 8.036 | 9.715 | 7.95 | 0.815 | -0.2951 | 0.0003563 | 0.002422 | no |
| ACKR3 | 7.182 | 6.337 | 5.119 | 5.428 | 6.76 | 5.274 | 0.815 | -0.295 | 0.05058 | 0.1442 | no |
| ZBTB41 | 2.418 | 2.093 | 1.778 | 1.839 | 2.256 | 1.808 | 0.816 | -0.2938 | 0.02376 | 0.08076 | no |
| TTC31 | 6.739 | 6.132 | 5.578 | 4.628 | 6.435 | 5.103 | 0.816 | -0.2938 | 0.0317 | 0.1009 | no |
| HNRNPF | 363.238 | 344.046 | 288.761 | 292.536 | 353.642 | 290.649 | 0.816 | -0.2935 | 9.594E-19 | 5.514E-17 | no |
| DDX31 | 12.326 | 12.031 | 10.532 | 9.276 | 12.178 | 9.904 | 0.816 | -0.2931 | 0.00185 | 0.009858 | no |
| TTLL4 | 2.171 | 1.986 | 1.632 | 1.639 | 2.079 | 1.635 | 0.817 | -0.2924 | 0.05488 | 0.1538 | no |
| TLE1 | 12.82 | 11.009 | 9.75 | 9.736 | 11.915 | 9.743 | 0.817 | -0.2913 | 0.005843 | 0.02602 | no |
| TEPSIN | 8.375 | 8.527 | 7.62 | 6.797 | 8.451 | 7.208 | 0.817 | -0.2912 | 0.02937 | 0.09516 | no |
| SRSF9 | 28.367 | 26.875 | 22.499 | 23.149 | 27.621 | 22.824 | 0.817 | -0.2909 | 0.001098 | 0.006373 | no |
| RNF41 | 7.038 | 6.94 | 5.461 | 5.867 | 6.989 | 5.664 | 0.817 | -0.2914 | 0.0013 | 0.007328 | no |
| RETSAT | 2.161 | 1.908 | 1.778 | 1.389 | 2.034 | 1.583 | 0.817 | -0.2908 | 0.04481 | 0.1316 | no |
| PPP4R1 | 59.43 | 58.48 | 48.526 | 48.418 | 58.955 | 48.472 | 0.817 | -0.292 | 1.031E-09 | 2.291E-08 | no |
| MAD2L1BP | 2.12 | 2.102 | 1.915 | 1.899 | 2.111 | 1.907 | 0.817 | -0.291 | 0.04816 | 0.1389 | no |
| FGF7 | 1.595 | 1.353 | 0.518 | 1.219 | 1.474 | 0.869 | 0.817 | -0.2916 | 0.1213 | 0.2739 | no |
| AKAP1 | 12.759 | 12.333 | 10.014 | 10.695 | 12.546 | 10.354 | 0.817 | -0.2916 | 0.0002064 | 0.001515 | no |
| ACTG1 | 4288.779 | 3798.806 | 3311.222 | 3327 | 4043.793 | 3319.111 | 0.817 | -0.2909 | 6.444E-08 | 0.000001035 | no |
| ZFAND6 | 51.096 | 44.23 | 36.753 | 40.761 | 47.663 | 38.757 | 0.818 | -0.2891 | 0.0004197 | 0.002798 | no |
| TAPT1 | 8.067 | 7.641 | 6.028 | 6.697 | 7.854 | 6.362 | 0.818 | -0.2892 | 0.01273 | 0.0494 | no |
| NOL11 | 54.162 | 49.583 | 42.517 | 42.7 | 51.873 | 42.609 | 0.818 | -0.2903 | 0.000002018 | 0.00002484 | no |
| NDUFV3 | 15.218 | 14.785 | 12.241 | 12.354 | 15.002 | 12.297 | 0.818 | -0.289 | 0.0001821 | 0.001351 | no |
| ID3 | 68.032 | 59.307 | 53.743 | 47.968 | 63.669 | 50.856 | 0.818 | -0.2896 | 0.003576 | 0.01724 | no |
| FBXO22 | 30.981 | 27.906 | 24.014 | 23.509 | 29.444 | 23.761 | 0.818 | -0.2907 | 0.00001172 | 0.0001177 | no |
| AMPD2 | 16.782 | 14.357 | 12.994 | 12.384 | 15.569 | 12.689 | 0.818 | -0.2894 | 0.000005736 | 0.00006249 | no |
| ZNF35 | 1.523 | 1.635 | 1.319 | 1.159 | 1.579 | 1.239 | 0.819 | -0.2881 | 0.0864 | 0.2149 | no |
| SV2C | 63.031 | 59.989 | 50.9 | 52.956 | 61.51 | 51.928 | 0.819 | -0.2887 | 1.668E-11 | 4.491E-10 | no |
| RASD1 | 0.967 | 0.934 | 0.401 | 0.52 | 0.951 | 0.461 | 0.819 | -0.2889 | 0.09963 | 0.2373 | no |
| PPP4R3A | 21.792 | 20.012 | 17.282 | 17.192 | 20.902 | 17.237 | 0.819 | -0.2876 | 0.00001146 | 0.0001154 | no |
| PDLIM7 | 167.785 | 153.802 | 129.799 | 129.92 | 160.793 | 129.859 | 0.819 | -0.2876 | 3.725E-11 | 9.752E-10 | no |
| NLK | 4.496 | 5.675 | 4.054 | 4.128 | 5.085 | 4.091 | 0.819 | -0.2878 | 0.03396 | 0.1064 | no |
| NAA60 | 17.79 | 17.336 | 14.019 | 14.663 | 17.563 | 14.341 | 0.819 | -0.2884 | 0.002405 | 0.01234 | no |
| DNAJC11 | 16.957 | 16.294 | 13.404 | 13.874 | 16.626 | 13.639 | 0.819 | -0.2881 | 0.0001054 | 0.0008363 | no |
| DHX36 | 11.833 | 11.807 | 9.486 | 10.005 | 11.82 | 9.745 | 0.819 | -0.2874 | 0.0001023 | 0.0008155 | no |
| ABCF3 | 14.323 | 14.484 | 12.603 | 10.965 | 14.404 | 11.784 | 0.819 | -0.2876 | 0.001435 | 0.00796 | no |
| SRP9 | 31.896 | 28.471 | 24.873 | 24.399 | 30.184 | 24.636 | 0.82 | -0.2861 | 0.001096 | 0.006362 | no |
| RMDN1 | 3.241 | 3.3 | 2.569 | 2.669 | 3.271 | 2.619 | 0.82 | -0.2861 | 0.0442 | 0.1301 | no |
| LIFR | 4.208 | 3.796 | 3.224 | 3.328 | 4.002 | 3.276 | 0.82 | -0.2856 | 0.002998 | 0.01493 | no |
| HIC1 | 19.189 | 17.044 | 14.518 | 14.793 | 18.117 | 14.655 | 0.82 | -0.2865 | 0.01088 | 0.04369 | no |
| HEXIM1 | 118.963 | 106.652 | 90.496 | 92.717 | 112.808 | 91.606 | 0.82 | -0.2858 | 0.000000554 | 0.000007571 | no |
| CENPK | 12.553 | 10.444 | 8.9 | 9.436 | 11.498 | 9.168 | 0.82 | -0.286 | 0.03992 | 0.1205 | no |
| ZNF296 | 2.017 | 2.141 | 1.671 | 1.629 | 2.079 | 1.65 | 0.821 | -0.2845 | 0.06171 | 0.1672 | no |
| TSFM | 44.346 | 37.971 | 33.422 | 33.854 | 41.158 | 33.638 | 0.821 | -0.2846 | 0.0009542 | 0.005677 | no |
| TFPI | 31.114 | 30.788 | 25.167 | 26.058 | 30.951 | 25.613 | 0.821 | -0.2844 | 0.000002499 | 0.00003008 | no |
| SMARCD1 | 31.763 | 29.415 | 24.698 | 25.798 | 30.589 | 25.248 | 0.821 | -0.2848 | 0.00001226 | 0.0001215 | no |
| PTPN1 | 36.331 | 34.701 | 29.495 | 29.446 | 35.516 | 29.471 | 0.821 | -0.2842 | 0.000001002 | 0.00001297 | no |
| NPLOC4 | 109.981 | 109.358 | 88.757 | 91.628 | 109.669 | 90.192 | 0.821 | -0.2846 | 1.848E-13 | 6.399E-12 | no |
| NAA25 | 5.155 | 4.896 | 3.957 | 4.268 | 5.026 | 4.112 | 0.821 | -0.2838 | 0.008508 | 0.03573 | no |
| LARP1B | 7.367 | 6.268 | 5.207 | 5.687 | 6.817 | 5.447 | 0.821 | -0.2847 | 0.0172 | 0.06287 | no |
| KLHL3 | 50.468 | 48.328 | 40.827 | 40.801 | 49.398 | 40.814 | 0.821 | -0.2853 | 1.189E-07 | 0.000001832 | no |
| GTF2IRD1 | 5.813 | 5.655 | 5.188 | 4.058 | 5.734 | 4.623 | 0.821 | -0.2854 | 0.03084 | 0.09865 | no |
| EXOC5 | 19.395 | 19.146 | 16.052 | 15.903 | 19.27 | 15.977 | 0.821 | -0.2845 | 7.317E-09 | 1.399E-07 | no |
| EIF4A2 | 85.997 | 79.359 | 67.059 | 68.169 | 82.678 | 67.614 | 0.821 | -0.2837 | 3.679E-07 | 0.000005144 | no |
| COX10 | 12.933 | 10.726 | 9.662 | 9.626 | 11.829 | 9.644 | 0.821 | -0.2846 | 0.006168 | 0.02724 | no |
| ZC3H4 | 34.366 | 33.133 | 28.146 | 27.807 | 33.749 | 27.977 | 0.822 | -0.2826 | 0.00001014 | 0.0001031 | no |
| RPTOR | 13.613 | 12.8 | 10.766 | 11.105 | 13.207 | 10.936 | 0.822 | -0.2823 | 0.00004335 | 0.0003781 | no |
| PPP1R8 | 57.732 | 51.335 | 43.348 | 44.18 | 54.534 | 43.764 | 0.822 | -0.2828 | 0.0005537 | 0.00356 | no |
| OSBPL5 | 33.45 | 28.335 | 26.437 | 24.139 | 30.893 | 25.288 | 0.822 | -0.2827 | 0.0004888 | 0.003198 | no |
| NACC1 | 146.549 | 126.771 | 106.977 | 108.84 | 136.66 | 107.909 | 0.822 | -0.2833 | 0.00003517 | 0.000315 | no |
| MAP3K11 | 6.667 | 6.93 | 5.764 | 5.368 | 6.798 | 5.566 | 0.822 | -0.283 | 0.007765 | 0.03306 | no |
| JMJD6 | 15.166 | 14.182 | 11.831 | 12.544 | 14.674 | 12.188 | 0.822 | -0.2827 | 0.000005493 | 0.00006013 | no |
| FZR1 | 13.15 | 12.303 | 10.922 | 10.295 | 12.727 | 10.608 | 0.822 | -0.2829 | 0.001166 | 0.0067 | no |
| FUT11 | 3.91 | 3.417 | 3.165 | 2.819 | 3.663 | 2.992 | 0.822 | -0.2832 | 0.04119 | 0.1233 | no |
| FEM1A | 20.517 | 19.808 | 16.11 | 16.692 | 20.163 | 16.401 | 0.822 | -0.2832 | 0.007297 | 0.03128 | no |
| ENOSF1 | 4.229 | 3.786 | 3.498 | 2.259 | 4.008 | 2.878 | 0.822 | -0.2822 | 0.1395 | 0.3018 | no |
| EMC2 | 27.493 | 25.668 | 21.268 | 21.35 | 26.581 | 21.309 | 0.822 | -0.282 | 0.01874 | 0.06717 | no |
| ARFGAP1 | 25.538 | 20.509 | 17.048 | 19.921 | 23.023 | 18.484 | 0.822 | -0.2832 | 0.006809 | 0.02958 | no |
| AMD1 | 46.075 | 47.023 | 30.413 | 42.55 | 46.549 | 36.481 | 0.822 | -0.2834 | 0.00001186 | 0.0001187 | no |
| ZSCAN26 | 2.202 | 1.986 | 1.749 | 1.559 | 2.094 | 1.654 | 0.823 | -0.2818 | 0.08852 | 0.2186 | no |
| ZNF275 | 5.227 | 6.2 | 4.181 | 4.768 | 5.713 | 4.474 | 0.823 | -0.2802 | 0.00534 | 0.02407 | no |
| WDR83 | 12.296 | 11.895 | 9.985 | 9.396 | 12.095 | 9.691 | 0.823 | -0.2802 | 0.04724 | 0.137 | no |
| ULK1 | 18.757 | 16.304 | 14.117 | 14.723 | 17.53 | 14.42 | 0.823 | -0.2807 | 0.000228 | 0.001646 | no |
| TUBGCP2 | 24.468 | 24.013 | 19.383 | 20.491 | 24.241 | 19.937 | 0.823 | -0.2803 | 0.00022 | 0.001599 | no |
| STK25 | 47.649 | 41.806 | 35.982 | 37.463 | 44.727 | 36.722 | 0.823 | -0.2814 | 0.000218 | 0.001587 | no |
| PUF60 | 224.602 | 200.504 | 173.274 | 177.548 | 212.553 | 175.411 | 0.823 | -0.2814 | 3.848E-11 | 1.001E-09 | no |
| MOGS | 7.84 | 7.475 | 6.047 | 6.237 | 7.657 | 6.142 | 0.823 | -0.2812 | 0.003305 | 0.01622 | no |
| Metazoa_SRP | 16.751 | 15.321 | 12.779 | 14.183 | 16.036 | 13.481 | 0.823 | -0.281 | 0.01109 | 0.04438 | no |
| INO80E | 30.775 | 26.524 | 22.714 | 24.159 | 28.649 | 23.436 | 0.823 | -0.2802 | 0.003417 | 0.01662 | no |
| GCNT1 | 9.219 | 8.011 | 6.135 | 6.147 | 8.615 | 6.141 | 0.823 | -0.2802 | 0.03177 | 0.101 | no |
| BICD2 | 15.886 | 15.068 | 12.544 | 13.064 | 15.477 | 12.804 | 0.823 | -0.2813 | 0.000155 | 0.001172 | no |
| ANKS3 | 11.493 | 11.048 | 8.529 | 9.766 | 11.271 | 9.148 | 0.823 | -0.2819 | 0.01661 | 0.06101 | no |
| SLU7 | 28.275 | 25.551 | 22.314 | 22.07 | 26.913 | 22.192 | 0.824 | -0.2785 | 0.0006536 | 0.004105 | no |
| RGS2 | 4.98 | 4.769 | 3.537 | 3.708 | 4.875 | 3.623 | 0.824 | -0.2792 | 0.1332 | 0.2921 | no |
| PDCD10 | 10.598 | 10.113 | 8.529 | 8.686 | 10.355 | 8.607 | 0.824 | -0.2794 | 0.0001208 | 0.0009444 | no |
| NTMT1 | 58.144 | 51.501 | 41.921 | 43.66 | 54.822 | 42.79 | 0.824 | -0.2793 | 0.0003089 | 0.002141 | no |
| NEDD1 | 20.187 | 19.798 | 16.921 | 16.053 | 19.992 | 16.487 | 0.824 | -0.2793 | 0.00013 | 0.001002 | no |
| MRTO4 | 32.997 | 30.924 | 26.71 | 26.118 | 31.96 | 26.414 | 0.824 | -0.2798 | 0.0006966 | 0.004328 | no |
| CDK8 | 5.546 | 5.217 | 4.387 | 4.338 | 5.381 | 4.362 | 0.824 | -0.2785 | 0.0385 | 0.1174 | no |
| APPL2 | 14.395 | 13.325 | 11.294 | 11.745 | 13.86 | 11.52 | 0.824 | -0.2785 | 0.003938 | 0.01873 | no |
| TRUB2 | 9.548 | 9.617 | 7.874 | 7.577 | 9.582 | 7.726 | 0.825 | -0.2779 | 0.03839 | 0.1171 | no |
| TMEM160 | 15.074 | 10.629 | 10.727 | 7.427 | 12.851 | 9.077 | 0.825 | -0.2769 | 0.1019 | 0.2413 | no |
| RSL1D1 | 77.704 | 68.574 | 59.585 | 60.962 | 73.139 | 60.273 | 0.825 | -0.2777 | 0.00002662 | 0.0002454 | no |
| RDH10 | 6.451 | 5.85 | 5.149 | 4.848 | 6.15 | 4.998 | 0.825 | -0.2769 | 0.02699 | 0.08913 | no |
| PGPEP1 | 21.329 | 17.949 | 13.355 | 17.422 | 19.639 | 15.389 | 0.825 | -0.2782 | 0.06443 | 0.1725 | no |
| HIP1R | 27.451 | 25.035 | 21.552 | 21.44 | 26.243 | 21.496 | 0.825 | -0.2783 | 0.0000121 | 0.0001203 | no |
| GMEB2 | 11.318 | 9.967 | 8.597 | 8.886 | 10.643 | 8.741 | 0.825 | -0.2784 | 0.005502 | 0.02469 | no |
| FBXO21 | 16.319 | 15.515 | 13.375 | 12.984 | 15.917 | 13.18 | 0.825 | -0.2783 | 0.00005653 | 0.000481 | no |
| EHMT1 | 15.146 | 14.377 | 12.466 | 12.004 | 14.762 | 12.235 | 0.825 | -0.2772 | 0.0002155 | 0.001575 | no |
| DDX47 | 11.935 | 11.943 | 9.408 | 10.435 | 11.939 | 9.921 | 0.825 | -0.2777 | 0.00002159 | 0.0002025 | no |
| C8orf33 | 36.804 | 36.18 | 31.79 | 32.335 | 36.492 | 32.063 | 0.825 | -0.2784 | 2.981E-07 | 0.000004304 | no |
| BRAT1 | 30.055 | 26.096 | 22.099 | 23.969 | 28.075 | 23.034 | 0.825 | -0.2775 | 0.0001849 | 0.001369 | no |
| ZNF354A | 8.087 | 7.913 | 6.223 | 6.437 | 8 | 6.33 | 0.826 | -0.2754 | 0.02947 | 0.09538 | no |
| ZBED1 | 32.997 | 29.328 | 24.776 | 26.208 | 31.163 | 25.492 | 0.826 | -0.2754 | 0.0004355 | 0.002889 | no |
| WDHD1 | 24.324 | 24.529 | 20.184 | 20.611 | 24.427 | 20.398 | 0.826 | -0.2755 | 1.307E-07 | 0.000002003 | no |
| TRIM28 | 278.61 | 243.283 | 213.545 | 219.709 | 260.947 | 216.627 | 0.826 | -0.2754 | 1.795E-11 | 4.808E-10 | no |
| SLC4A1AP | 23.974 | 22.689 | 19.168 | 19.351 | 23.331 | 19.259 | 0.826 | -0.275 | 0.001005 | 0.005922 | no |
| SHMT1 | 24.303 | 22.514 | 18.406 | 20.271 | 23.409 | 19.338 | 0.826 | -0.2759 | 0.001117 | 0.006466 | no |
| NDNF | 2.655 | 2.628 | 2.188 | 2.129 | 2.641 | 2.159 | 0.826 | -0.2756 | 0.1008 | 0.2395 | no |
| NAB1 | 5.165 | 5.295 | 4.474 | 4.258 | 5.23 | 4.366 | 0.826 | -0.2756 | 0.01382 | 0.05289 | no |
| MUM1 | 8.509 | 9.033 | 7.063 | 7.377 | 8.771 | 7.22 | 0.826 | -0.2759 | 0.02297 | 0.07868 | no |
| MRPL54 | 17.049 | 17.238 | 13.648 | 13.614 | 17.143 | 13.631 | 0.826 | -0.2752 | 0.00379 | 0.01815 | no |
| KLHL18 | 11.421 | 11.038 | 9.037 | 9.536 | 11.229 | 9.287 | 0.826 | -0.2763 | 0.000275 | 0.00194 | no |
| GALR2 | 2.459 | 2.648 | 1.026 | 2.229 | 2.554 | 1.627 | 0.826 | -0.2759 | 0.1501 | 0.3168 | no |
| FNBP4 | 16.205 | 15.681 | 12.984 | 13.404 | 15.943 | 13.194 | 0.826 | -0.2759 | 0.0003987 | 0.002669 | no |
| ERBIN | 31.804 | 31.177 | 26.153 | 26.378 | 31.49 | 26.265 | 0.826 | -0.2754 | 6.578E-10 | 1.494E-08 | no |
| DDX50 | 17.049 | 15.778 | 13.492 | 13.504 | 16.413 | 13.498 | 0.826 | -0.2765 | 0.004406 | 0.02047 | no |
| CDC34 | 68.629 | 57.74 | 52.121 | 51.526 | 63.184 | 51.824 | 0.826 | -0.2761 | 0.0004327 | 0.002872 | no |
| C4orf33 | 3.879 | 3.67 | 3.058 | 2.989 | 3.774 | 3.023 | 0.826 | -0.275 | 0.06227 | 0.1682 | no |
| BAP1 | 57.372 | 52.221 | 44.764 | 46.469 | 54.796 | 45.617 | 0.826 | -0.2751 | 1.565E-07 | 0.000002385 | no |
| ZSCAN16 | 13.201 | 12.177 | 10.444 | 10.185 | 12.689 | 10.315 | 0.827 | -0.2739 | 0.002881 | 0.01444 | no |
| USP27X | 3.478 | 4.059 | 2.863 | 2.559 | 3.769 | 2.711 | 0.827 | -0.2734 | 0.1553 | 0.3247 | no |
| TIMM8A | 15.382 | 16.207 | 13.082 | 11.725 | 15.794 | 12.404 | 0.827 | -0.2742 | 0.03046 | 0.09778 | no |
| RBM18 | 12.316 | 9.773 | 9.008 | 9.216 | 11.044 | 9.112 | 0.827 | -0.2737 | 0.005386 | 0.02426 | no |
| PES1 | 87.334 | 78.93 | 70.185 | 67.839 | 83.132 | 69.012 | 0.827 | -0.2735 | 6.464E-07 | 0.00000869 | no |
| ORAI2 | 16.339 | 12.897 | 12.3 | 11.105 | 14.618 | 11.703 | 0.827 | -0.2745 | 0.003395 | 0.01654 | no |
| KAT5 | 12.378 | 9.743 | 8.91 | 9.166 | 11.061 | 9.038 | 0.827 | -0.2742 | 0.009843 | 0.04025 | no |
| FBXL19 | 16.535 | 13.111 | 12.3 | 11.225 | 14.823 | 11.762 | 0.827 | -0.2745 | 0.01053 | 0.04252 | no |
| CUL1 | 54.533 | 50.012 | 41.98 | 44.699 | 52.273 | 43.34 | 0.827 | -0.2745 | 6.391E-07 | 0.000008602 | no |
| CRLF3 | 12.018 | 11.797 | 9.301 | 10.365 | 11.908 | 9.833 | 0.827 | -0.2749 | 0.01214 | 0.04749 | no |
| B3GNT8 | 5.042 | 4.039 | 3.879 | 2.759 | 4.54 | 3.319 | 0.827 | -0.2749 | 0.1468 | 0.3123 | no |
| TOM1L2 | 21.082 | 19.282 | 16.433 | 17.222 | 20.182 | 16.828 | 0.828 | -0.2728 | 0.00001662 | 0.00016 | no |
| SLC25A17 | 16.38 | 14.941 | 12.808 | 14.363 | 15.66 | 13.585 | 0.828 | -0.2723 | 0.00004142 | 0.0003648 | no |
| SHQ1 | 7.13 | 6.979 | 5.559 | 5.967 | 7.054 | 5.763 | 0.828 | -0.273 | 0.03118 | 0.09952 | no |
| PACRGL | 8.663 | 6.93 | 5.793 | 5.937 | 7.796 | 5.865 | 0.828 | -0.2723 | 0.06202 | 0.1678 | no |
| CTU2 | 24.313 | 22.261 | 19.236 | 18.591 | 23.287 | 18.913 | 0.828 | -0.2724 | 0.003544 | 0.01713 | no |
| XPO4 | 25.414 | 26.67 | 23.154 | 23.539 | 26.042 | 23.346 | 0.829 | -0.271 | 0.000281 | 0.001978 | no |
| USB1 | 3.467 | 3.592 | 2.706 | 2.889 | 3.53 | 2.797 | 0.829 | -0.2703 | 0.105 | 0.2474 | no |
| RPAP3 | 14.837 | 14.893 | 11.899 | 12.964 | 14.865 | 12.431 | 0.829 | -0.2709 | 0.0003211 | 0.00221 | no |
| PNPT1 | 20.218 | 19.282 | 15.143 | 17.602 | 19.75 | 16.373 | 0.829 | -0.2709 | 0.003084 | 0.01532 | no |
| PFDN4 | 21.566 | 21.395 | 17.712 | 17.402 | 21.48 | 17.557 | 0.829 | -0.2707 | 0.01499 | 0.05637 | no |
| MCRS1 | 71.458 | 65.371 | 57.143 | 56.044 | 68.415 | 56.593 | 0.829 | -0.2706 | 0.00001314 | 0.0001298 | no |
| GPCPD1 | 8.9 | 8.332 | 6.829 | 8.026 | 8.616 | 7.428 | 0.829 | -0.2712 | 0.02175 | 0.07547 | no |
| FAM222B | 4.795 | 5.869 | 4.641 | 4.988 | 5.332 | 4.815 | 0.829 | -0.2705 | 0.06053 | 0.1648 | no |
| EDC3 | 15.537 | 14.766 | 12.984 | 12.194 | 15.152 | 12.589 | 0.829 | -0.2714 | 0.0005575 | 0.003572 | no |
| ZNF584 | 1.554 | 1.421 | 1.016 | 0.98 | 1.488 | 0.998 | 0.83 | -0.2692 | 0.1651 | 0.3382 | no |
| REPIN1 | 11.863 | 10.357 | 9.017 | 9.316 | 11.11 | 9.166 | 0.83 | -0.2687 | 0.01224 | 0.04782 | no |
| PUSL1 | 2.716 | 2.433 | 1.719 | 1.469 | 2.575 | 1.594 | 0.83 | -0.2689 | 0.1584 | 0.3289 | no |
| PRELID3A | 5.638 | 5.529 | 5.139 | 4.138 | 5.583 | 4.639 | 0.83 | -0.2693 | 0.03905 | 0.1186 | no |
| MRPS18A | 57.578 | 47.899 | 42.088 | 42.91 | 52.739 | 42.499 | 0.83 | -0.268 | 0.01513 | 0.05676 | no |
| MAP2K3 | 92.736 | 83.71 | 73.429 | 73.646 | 88.223 | 73.537 | 0.83 | -0.2687 | 2.738E-07 | 0.000003988 | no |
| ESS2 | 26.104 | 25.074 | 20.233 | 21.97 | 25.589 | 21.102 | 0.83 | -0.2686 | 0.00606 | 0.02684 | no |
| DRG2 | 7.789 | 7.466 | 5.979 | 6.537 | 7.627 | 6.258 | 0.83 | -0.2681 | 0.03169 | 0.1009 | no |
| DLK2 | 12.748 | 10.746 | 9.008 | 10.135 | 11.747 | 9.572 | 0.83 | -0.2688 | 0.04287 | 0.127 | no |
| DAZAP1 | 65.542 | 59.463 | 51.828 | 52.666 | 62.502 | 52.247 | 0.83 | -0.2694 | 0.000008427 | 0.00008765 | no |
| YWHAZ | 111.174 | 106.662 | 91.952 | 91.178 | 108.918 | 91.565 | 0.831 | -0.2663 | 6.94E-15 | 2.809E-13 | no |
| WDR47 | 2.14 | 1.888 | 1.544 | 1.639 | 2.014 | 1.591 | 0.831 | -0.2671 | 0.1141 | 0.2624 | no |
| SPIRE1 | 12.059 | 11.262 | 9.74 | 9.915 | 11.66 | 9.828 | 0.831 | -0.2664 | 0.00073 | 0.004503 | no |
| NR2C2AP | 16.113 | 18.679 | 11.108 | 11.165 | 17.396 | 11.136 | 0.831 | -0.2664 | 0.1664 | 0.3398 | no |
| MRPL39 | 14.58 | 13.335 | 11.831 | 11.015 | 13.957 | 11.423 | 0.831 | -0.2666 | 0.02523 | 0.08461 | no |
| LSG1 | 13.119 | 12.313 | 10.454 | 10.795 | 12.716 | 10.625 | 0.831 | -0.2668 | 0.001035 | 0.006063 | no |
| KDM5B | 2.593 | 2.755 | 2.159 | 2.239 | 2.674 | 2.199 | 0.831 | -0.2663 | 0.03226 | 0.1022 | no |
| FGFR1OP | 7.83 | 7.106 | 6.233 | 5.987 | 7.468 | 6.11 | 0.831 | -0.2672 | 0.03905 | 0.1186 | no |
| FBXO18 | 9.98 | 9.325 | 7.943 | 7.966 | 9.652 | 7.954 | 0.831 | -0.267 | 0.008217 | 0.03471 | no |
| CSNK1A1 | 74.895 | 72.818 | 60.943 | 62.841 | 73.856 | 61.892 | 0.831 | -0.2678 | 4.239E-11 | 1.093E-09 | no |
| CD44 | 88.682 | 79.378 | 71.318 | 69.588 | 84.03 | 70.453 | 0.831 | -0.2665 | 3.36E-09 | 6.763E-08 | no |
| ZNFX1 | 37.525 | 36.015 | 30.071 | 31.825 | 36.77 | 30.948 | 0.832 | -0.265 | 3.684E-09 | 7.364E-08 | no |
| ZBTB7B | 17.646 | 17.141 | 13.453 | 15.883 | 17.393 | 14.668 | 0.832 | -0.2646 | 0.04474 | 0.1315 | no |
| WDR45B | 32.802 | 31.634 | 26.554 | 25.558 | 32.218 | 26.056 | 0.832 | -0.2647 | 0.00001022 | 0.0001037 | no |
| WAC | 18.14 | 16.917 | 14.488 | 14.683 | 17.529 | 14.585 | 0.832 | -0.2653 | 0.0000794 | 0.0006523 | no |
| VIPAS39 | 20.187 | 19.36 | 16.755 | 16.622 | 19.773 | 16.688 | 0.832 | -0.2649 | 2.464E-07 | 0.000003617 | no |
| UTP15 | 7.902 | 8.05 | 6.155 | 7.137 | 7.976 | 6.646 | 0.832 | -0.2646 | 0.006371 | 0.02797 | no |
| TRMT61A | 28.943 | 26.213 | 22.734 | 23.009 | 27.578 | 22.872 | 0.832 | -0.2651 | 0.0009327 | 0.005558 | no |
| TRAF2 | 10.341 | 8.468 | 8.06 | 7.167 | 9.404 | 7.614 | 0.832 | -0.266 | 0.04281 | 0.1269 | no |
| RPP14 | 7.336 | 7.232 | 5.969 | 6.057 | 7.284 | 6.013 | 0.832 | -0.2655 | 0.01374 | 0.05269 | no |
| RBM19 | 18.284 | 18.036 | 14.987 | 14.873 | 18.16 | 14.93 | 0.832 | -0.2651 | 0.0002783 | 0.001961 | no |
| POLR2H | 30.744 | 23.341 | 20.184 | 22.78 | 27.043 | 21.482 | 0.832 | -0.2657 | 0.06476 | 0.1731 | no |
| OSBPL3 | 76.057 | 74.939 | 62.34 | 65.05 | 75.498 | 63.695 | 0.832 | -0.2645 | 1.138E-13 | 4.024E-12 | no |
| MTR | 2.912 | 2.813 | 2.247 | 2.449 | 2.862 | 2.348 | 0.832 | -0.2649 | 0.02604 | 0.08674 | no |
| MFSD14B | 33.316 | 31.566 | 26.827 | 27.367 | 32.441 | 27.097 | 0.832 | -0.2662 | 0.00007184 | 0.0005975 | no |
| DNAJA3 | 35.498 | 32.316 | 27.941 | 28.547 | 33.907 | 28.244 | 0.832 | -0.2654 | 0.000182 | 0.001351 | no |
| DDX55 | 11.39 | 10.756 | 9.252 | 9.146 | 11.073 | 9.199 | 0.832 | -0.2652 | 0.008345 | 0.03517 | no |
| CMTM6 | 24.704 | 23.059 | 19.1 | 19.091 | 23.882 | 19.096 | 0.832 | -0.2649 | 0.000816 | 0.004941 | no |
| SLC20A1 | 29.015 | 27.235 | 24.151 | 23.039 | 28.125 | 23.595 | 0.833 | -0.2644 | 0.000006664 | 0.00007119 | no |
| SF3B4 | 62.342 | 61.196 | 51.613 | 51.206 | 61.769 | 51.41 | 0.833 | -0.2629 | 0.0001849 | 0.001369 | no |
| RTN4IP1 | 5.433 | 5.461 | 4.26 | 4.518 | 5.447 | 4.389 | 0.833 | -0.263 | 0.04336 | 0.1281 | no |
| POLR3F | 4.188 | 4.283 | 2.999 | 3.868 | 4.236 | 3.433 | 0.833 | -0.2628 | 0.0815 | 0.2061 | no |
| PDXDC1 | 51.23 | 48.98 | 41.951 | 42.291 | 50.105 | 42.121 | 0.833 | -0.2631 | 5.863E-08 | 9.518E-07 | no |
| NFRKB | 6.05 | 6.084 | 5.129 | 4.998 | 6.067 | 5.063 | 0.833 | -0.2636 | 0.009796 | 0.04008 | no |
| NCBP3 | 6.235 | 6.113 | 5.373 | 5.048 | 6.174 | 5.21 | 0.833 | -0.2632 | 0.003133 | 0.01554 | no |
| KHDRBS1 | 140.859 | 128.883 | 112.956 | 113.478 | 134.871 | 113.217 | 0.833 | -0.2631 | 8.949E-11 | 2.236E-09 | no |
| GPR68 | 20.054 | 16.732 | 14.742 | 15.743 | 18.393 | 15.242 | 0.833 | -0.2639 | 0.004992 | 0.02266 | no |
| COLEC12 | 0.731 | 0.574 | 0.352 | 0.31 | 0.652 | 0.331 | 0.833 | -0.2635 | 0.1571 | 0.327 | no |
| CMC2 | 22.225 | 20.947 | 17.605 | 18.651 | 21.586 | 18.128 | 0.833 | -0.2633 | 0.009587 | 0.03937 | no |
| ATXN7L2 | 1.976 | 2.385 | 1.622 | 1.649 | 2.18 | 1.635 | 0.833 | -0.2638 | 0.1633 | 0.3356 | no |
| AADAT | 16.36 | 13.87 | 12.964 | 12.704 | 15.115 | 12.834 | 0.833 | -0.2632 | 0.02032 | 0.07151 | no |
| VPS4B | 19.323 | 18.095 | 15.065 | 16.173 | 18.709 | 15.619 | 0.834 | -0.2627 | 0.001099 | 0.006373 | no |
| NTN1 | 16.648 | 17.793 | 14.518 | 13.734 | 17.221 | 14.126 | 0.834 | -0.2624 | 0.02996 | 0.09667 | no |
| NFKBIB | 33.409 | 32.082 | 27.257 | 24.919 | 32.745 | 26.088 | 0.834 | -0.2615 | 0.01825 | 0.06574 | no |
| NARFL | 4.589 | 4.341 | 3.693 | 3.738 | 4.465 | 3.716 | 0.834 | -0.2612 | 0.01566 | 0.05824 | no |
| INTS9 | 7.305 | 6.716 | 5.901 | 5.647 | 7.011 | 5.774 | 0.834 | -0.2627 | 0.02834 | 0.09264 | no |
| FAM102A | 4.538 | 4.195 | 3.41 | 3.748 | 4.367 | 3.579 | 0.834 | -0.262 | 0.05268 | 0.1491 | no |
| DLD | 32.853 | 29.882 | 26.945 | 25.438 | 31.367 | 26.191 | 0.834 | -0.2614 | 0.0005618 | 0.003593 | no |
| CHD1 | 22.935 | 21.823 | 21.542 | 18.521 | 22.379 | 20.032 | 0.834 | -0.2619 | 0.0000544 | 0.0004649 | no |
| BECN1 | 51.065 | 47.422 | 40.964 | 41.481 | 49.243 | 41.222 | 0.834 | -0.2615 | 0.00008039 | 0.0006596 | no |
| ZNF169 | 3.57 | 3.631 | 3.175 | 2.369 | 3.6 | 2.772 | 0.835 | -0.2597 | 0.1487 | 0.3151 | no |
| WDFY1 | 16.339 | 16.703 | 13.677 | 14.114 | 16.521 | 13.896 | 0.835 | -0.2599 | 0.0002968 | 0.002069 | no |
| TOMM70 | 24.91 | 24.023 | 20.79 | 20.341 | 24.466 | 20.566 | 0.835 | -0.2594 | 0.00005533 | 0.0004721 | no |
| TMEM87A | 27.462 | 25.23 | 21.689 | 22.58 | 26.346 | 22.134 | 0.835 | -0.26 | 0.00008547 | 0.0006953 | no |
| ORC4 | 9.271 | 8.011 | 6.966 | 7.357 | 8.641 | 7.162 | 0.835 | -0.2606 | 0.02012 | 0.07097 | no |
| GLYCTK | 7.141 | 6.161 | 5.627 | 5.378 | 6.651 | 5.502 | 0.835 | -0.2603 | 0.01996 | 0.07058 | no |
| GINS4 | 41.98 | 38.263 | 34.067 | 32.675 | 40.121 | 33.371 | 0.835 | -0.2605 | 0.001415 | 0.007877 | no |
| CUL2 | 6.472 | 5.918 | 5.061 | 5.258 | 6.195 | 5.159 | 0.835 | -0.2597 | 0.01417 | 0.05389 | no |
| C8orf89 | 11.627 | 10.094 | 8.333 | 10.815 | 10.861 | 9.574 | 0.835 | -0.2608 | 0.1202 | 0.2722 | no |
| C7orf43 | 16.957 | 14.776 | 14.205 | 12.154 | 15.867 | 13.18 | 0.835 | -0.2598 | 0.009536 | 0.03923 | no |
| AURKAIP1 | 24.015 | 15.603 | 15.475 | 15.993 | 19.809 | 15.734 | 0.835 | -0.2599 | 0.08949 | 0.2202 | no |
| AUP1 | 41.085 | 41.125 | 34.262 | 36.483 | 41.105 | 35.373 | 0.835 | -0.2609 | 0.00001483 | 0.0001441 | no |
| ZBTB11 | 3.076 | 2.949 | 2.442 | 2.619 | 3.013 | 2.53 | 0.836 | -0.2578 | 0.01446 | 0.05477 | no |
| USP12 | 14.95 | 13.033 | 12.007 | 11.565 | 13.991 | 11.786 | 0.836 | -0.2587 | 0.002589 | 0.01313 | no |
| TRIT1 | 4.651 | 4.293 | 3.957 | 3.328 | 4.472 | 3.643 | 0.836 | -0.2576 | 0.1388 | 0.3006 | no |
| TOMM20 | 99.342 | 95.487 | 80.941 | 83.442 | 97.415 | 82.191 | 0.836 | -0.2586 | 1.484E-10 | 3.613E-09 | no |
| SLC43A3 | 11.555 | 9.374 | 8.773 | 8.446 | 10.465 | 8.61 | 0.836 | -0.2584 | 0.03936 | 0.1192 | no |
| RUVBL2 | 63.793 | 57.711 | 51.73 | 49.677 | 60.752 | 50.703 | 0.836 | -0.2581 | 0.0002249 | 0.001629 | no |
| RCC2 | 37.946 | 34.739 | 30.696 | 30.616 | 36.343 | 30.656 | 0.836 | -0.2577 | 0.00001488 | 0.0001444 | no |
| PDPK1 | 11.75 | 10.59 | 9.604 | 9.186 | 11.17 | 9.395 | 0.836 | -0.2579 | 0.0004152 | 0.002771 | no |
| NAA50 | 30.775 | 29.6 | 25.206 | 25.788 | 30.188 | 25.497 | 0.836 | -0.2589 | 0.000001128 | 0.00001442 | no |
| MAPK6 | 87.108 | 80.488 | 69.677 | 71.797 | 83.798 | 70.737 | 0.836 | -0.2586 | 1.748E-10 | 4.203E-09 | no |
| KDM6A | 8.334 | 7.592 | 6.653 | 6.587 | 7.963 | 6.62 | 0.836 | -0.2579 | 0.009051 | 0.03762 | no |
| HSPB8 | 51.538 | 48.59 | 41.746 | 42.031 | 50.064 | 41.889 | 0.836 | -0.2586 | 0.0001825 | 0.001353 | no |
| DLX2 | 2.171 | 1.947 | 1.387 | 1.729 | 2.059 | 1.558 | 0.836 | -0.2586 | 0.1699 | 0.3446 | no |
| COG2 | 24.509 | 23.789 | 20.614 | 19.781 | 24.149 | 20.197 | 0.836 | -0.2583 | 0.0007213 | 0.004465 | no |
| ACIN1 | 61.56 | 60.008 | 51.623 | 48.438 | 60.784 | 50.031 | 0.836 | -0.2592 | 9.305E-08 | 0.000001461 | no |
| ZNF777 | 14.981 | 14.114 | 12.29 | 11.765 | 14.547 | 12.027 | 0.837 | -0.2568 | 0.01238 | 0.04827 | no |
| TEAD3 | 31.886 | 30.924 | 26.73 | 25.898 | 31.405 | 26.314 | 0.837 | -0.2568 | 0.0002709 | 0.001919 | no |
| TCOF1 | 14.364 | 13.462 | 11.557 | 11.855 | 13.913 | 11.706 | 0.837 | -0.2575 | 0.0007533 | 0.00461 | no |
| SNORD65 | 103.396 | 91.672 | 79.075 | 81.473 | 97.534 | 80.274 | 0.837 | -0.256 | 0.00177 | 0.009517 | no |
| RFK | 8.55 | 7.407 | 6.184 | 6.907 | 7.979 | 6.546 | 0.837 | -0.2559 | 0.05379 | 0.1514 | no |
| RARS | 105.063 | 98.845 | 84.595 | 86.57 | 101.954 | 85.582 | 0.837 | -0.2561 | 9.058E-08 | 0.000001424 | no |
| PIP5K1B | 3.046 | 2.959 | 2.452 | 2.409 | 3.002 | 2.43 | 0.837 | -0.2575 | 0.09744 | 0.2334 | no |
| PARM1 | 1.019 | 0.788 | 0.303 | 0.29 | 0.903 | 0.296 | 0.837 | -0.257 | 0.1076 | 0.2516 | no |
| KCTD18 | 4.208 | 3.504 | 3.087 | 3.169 | 3.856 | 3.128 | 0.837 | -0.2571 | 0.08101 | 0.2052 | no |
| GBA2 | 3.437 | 3.027 | 2.95 | 2.249 | 3.232 | 2.599 | 0.837 | -0.2572 | 0.1047 | 0.2471 | no |
| FASTKD2 | 12.409 | 12.459 | 10.414 | 10.275 | 12.434 | 10.345 | 0.837 | -0.2572 | 0.01467 | 0.05535 | no |
| ELP1 | 13.808 | 13.316 | 12.134 | 11.285 | 13.562 | 11.71 | 0.837 | -0.2572 | 0.0003559 | 0.002422 | no |
| EGFL6 | 0.401 | 0.448 | 0.234 | 0.33 | 0.424 | 0.282 | 0.837 | -0.256 | 0.1845 | 0.3651 | no |
| CRTC3 | 13.345 | 12.897 | 10.766 | 11.145 | 13.121 | 10.956 | 0.837 | -0.2573 | 0.0007341 | 0.004522 | no |
| CRIM1 | 21.206 | 18.786 | 16.716 | 16.762 | 19.996 | 16.739 | 0.837 | -0.2568 | 0.0001185 | 0.0009286 | no |
| CCL22 | 0.875 | 0.633 | 0.508 | 0.28 | 0.754 | 0.394 | 0.837 | -0.2569 | 0.1408 | 0.3035 | no |
| CCDC58 | 35.446 | 27.021 | 24.786 | 24.609 | 31.233 | 24.698 | 0.837 | -0.2575 | 0.08126 | 0.2056 | no |
| ARMC8 | 13.787 | 13.88 | 10.248 | 11.385 | 13.834 | 10.816 | 0.837 | -0.2564 | 0.008147 | 0.03446 | no |
| ARMC6 | 6.029 | 5.83 | 4.904 | 4.988 | 5.929 | 4.946 | 0.837 | -0.2565 | 0.01755 | 0.06376 | no |
| TMEM69 | 12.563 | 13.17 | 9.809 | 10.845 | 12.867 | 10.327 | 0.838 | -0.2557 | 0.05623 | 0.1562 | no |
| TACO1 | 5.011 | 4.477 | 3.781 | 4.408 | 4.744 | 4.095 | 0.838 | -0.2555 | 0.01578 | 0.05861 | no |
| SYNC | 27.184 | 28.247 | 24.258 | 22.56 | 27.715 | 23.409 | 0.838 | -0.2542 | 0.002321 | 0.01199 | no |
| SUPT7L | 9.096 | 7.252 | 7.093 | 6.377 | 8.174 | 6.735 | 0.838 | -0.2546 | 0.03905 | 0.1186 | no |
| SMNDC1 | 9.538 | 8.838 | 6.956 | 8.186 | 9.188 | 7.571 | 0.838 | -0.2547 | 0.02497 | 0.08392 | no |
| SLC30A6 | 10.135 | 9.636 | 8.304 | 8.226 | 9.886 | 8.265 | 0.838 | -0.2543 | 0.009383 | 0.03881 | no |
| RHOBTB3 | 23.634 | 21.463 | 19.061 | 18.971 | 22.549 | 19.016 | 0.838 | -0.2551 | 0.0000775 | 0.0006381 | no |
| PSMD12 | 76.695 | 69.683 | 60.318 | 63.271 | 73.189 | 61.794 | 0.838 | -0.2556 | 3.027E-08 | 5.139E-07 | no |
| POLR3A | 9.692 | 9.237 | 7.874 | 8.096 | 9.465 | 7.985 | 0.838 | -0.2547 | 0.0008174 | 0.004947 | no |
| NAA35 | 13.633 | 12.547 | 11.46 | 10.505 | 13.09 | 10.983 | 0.838 | -0.2543 | 0.003799 | 0.01818 | no |
| MRPL16 | 60.727 | 56.27 | 45.653 | 50.817 | 58.498 | 48.235 | 0.838 | -0.2548 | 0.006951 | 0.03006 | no |
| MCMBP | 29.129 | 26.553 | 23.213 | 23.369 | 27.841 | 23.291 | 0.838 | -0.2548 | 0.000009987 | 0.0001019 | no |
| GART | 28.172 | 27.712 | 22.597 | 23.039 | 27.942 | 22.818 | 0.838 | -0.2549 | 0.00002047 | 0.0001927 | no |
| G3BP1 | 110.763 | 109.397 | 92.821 | 92.787 | 110.08 | 92.804 | 0.838 | -0.2556 | 3.167E-08 | 5.345E-07 | no |
| CLP1 | 15.341 | 14.639 | 13.072 | 12.394 | 14.99 | 12.733 | 0.838 | -0.2548 | 0.01514 | 0.05677 | no |
| BTAF1 | 12.018 | 11.943 | 10.17 | 10.055 | 11.98 | 10.113 | 0.838 | -0.2542 | 0.0007889 | 0.004803 | no |
| YTHDF2 | 30.693 | 27.537 | 25.04 | 22.81 | 29.115 | 23.925 | 0.839 | -0.2534 | 0.0005866 | 0.003725 | no |
| TCTA | 9.847 | 10.882 | 8.5 | 8.146 | 10.364 | 8.323 | 0.839 | -0.2537 | 0.09317 | 0.2264 | no |
| SULF1 | 58.628 | 57.049 | 48.838 | 49.167 | 57.838 | 49.002 | 0.839 | -0.2538 | 2.925E-10 | 6.918E-09 | no |
| RNF40 | 15.043 | 14.026 | 11.841 | 12.714 | 14.534 | 12.277 | 0.839 | -0.2541 | 0.0001704 | 0.001276 | no |
| RIF1 | 2.922 | 3.086 | 2.56 | 2.579 | 3.004 | 2.57 | 0.839 | -0.2533 | 0.01527 | 0.05713 | no |
| RASSF8 | 8.437 | 7.69 | 7.044 | 6.417 | 8.063 | 6.73 | 0.839 | -0.2539 | 0.007585 | 0.03237 | no |
| POLRMT | 53.329 | 49.34 | 41.335 | 40.132 | 51.335 | 40.733 | 0.839 | -0.2529 | 0.01009 | 0.04104 | no |
| PLA2G4A | 3.118 | 2.696 | 2.276 | 2.449 | 2.907 | 2.362 | 0.839 | -0.2534 | 0.1033 | 0.2441 | no |
| PHLDB1 | 41.218 | 39.762 | 34.741 | 36.493 | 40.49 | 35.617 | 0.839 | -0.2524 | 1.657E-11 | 4.472E-10 | no |
| KIF7 | 16.957 | 16.654 | 13.091 | 12.744 | 16.806 | 12.918 | 0.839 | -0.253 | 0.06913 | 0.1824 | no |
| HCFC2 | 2.48 | 2.686 | 2.061 | 2.139 | 2.583 | 2.1 | 0.839 | -0.2526 | 0.03784 | 0.1159 | no |
| ESF1 | 21.885 | 20.996 | 18.045 | 17.942 | 21.441 | 17.994 | 0.839 | -0.2527 | 0.001055 | 0.006156 | no |
| DPH7 | 19.91 | 15.895 | 14.313 | 15.023 | 17.902 | 14.668 | 0.839 | -0.2532 | 0.01554 | 0.05795 | no |
| CNST | 4.507 | 4.078 | 3.664 | 3.778 | 4.293 | 3.721 | 0.839 | -0.2531 | 0.03802 | 0.1163 | no |
| CA10 | 13.005 | 13.248 | 11.098 | 10.685 | 13.127 | 10.892 | 0.839 | -0.254 | 0.008012 | 0.03397 | no |
| ADPRM | 2.85 | 2.599 | 2.257 | 2.119 | 2.724 | 2.188 | 0.839 | -0.2528 | 0.1261 | 0.2812 | no |
| ACTR6 | 15.207 | 13.598 | 11.86 | 11.984 | 14.402 | 11.922 | 0.839 | -0.2528 | 0.02998 | 0.09668 | no |
| TMEM115 | 40.261 | 35.654 | 32.386 | 31.366 | 37.958 | 31.876 | 0.84 | -0.2516 | 0.001001 | 0.005915 | no |
| TFIP11 | 30.507 | 28.899 | 24.17 | 26.068 | 29.703 | 25.119 | 0.84 | -0.2511 | 0.0001665 | 0.001249 | no |
| TAOK3 | 7.213 | 6.745 | 5.969 | 5.727 | 6.979 | 5.848 | 0.84 | -0.2509 | 0.023 | 0.07874 | no |
| STK35 | 4.784 | 4.331 | 3.849 | 3.808 | 4.558 | 3.829 | 0.84 | -0.2509 | 0.02183 | 0.07561 | no |
| SLC33A1 | 11.153 | 11.096 | 9.74 | 9.336 | 11.125 | 9.538 | 0.84 | -0.2507 | 0.001144 | 0.006594 | no |
| SLC16A1 | 17.09 | 15.817 | 13.365 | 14.543 | 16.453 | 13.954 | 0.84 | -0.2509 | 0.0001177 | 0.0009233 | no |
| PSME3 | 50.787 | 48.075 | 39.166 | 39.812 | 49.431 | 39.489 | 0.84 | -0.2522 | 4.134E-09 | 8.193E-08 | no |
| PDRG1 | 63.33 | 51.559 | 49.171 | 46.389 | 57.444 | 47.78 | 0.84 | -0.2514 | 0.004577 | 0.02111 | no |
| NUDT1 | 106.472 | 91.876 | 81.137 | 84.151 | 99.174 | 82.644 | 0.84 | -0.251 | 0.0005194 | 0.003371 | no |
| NOP9 | 21.268 | 20.324 | 17.869 | 17.012 | 20.796 | 17.441 | 0.84 | -0.2521 | 0.003889 | 0.01853 | no |
| KITLG | 27.328 | 27.799 | 23.301 | 22.75 | 27.563 | 23.026 | 0.84 | -0.2512 | 0.00001449 | 0.0001414 | no |
| ICAM1 | 102.562 | 95.77 | 83.462 | 84.521 | 99.166 | 83.992 | 0.84 | -0.2508 | 3.849E-09 | 7.667E-08 | no |
| EGR4 | 0.185 | 0.195 | 0.117 | 0.04 | 0.19 | 0.079 | 0.84 | -0.2513 | 0.1106 | 0.2567 | no |
| DUS1L | 80.914 | 73.46 | 64.167 | 65.65 | 77.187 | 64.909 | 0.84 | -0.2518 | 0.00002341 | 0.0002182 | no |
| DNTTIP1 | 56.364 | 49.184 | 43.993 | 43.93 | 52.774 | 43.962 | 0.84 | -0.251 | 0.003483 | 0.01687 | no |
| DHX8 | 18.315 | 17.277 | 15.084 | 15.093 | 17.796 | 15.088 | 0.84 | -0.2508 | 0.00003432 | 0.0003082 | no |
| C18orf25 | 6.575 | 6.385 | 5.373 | 5.517 | 6.48 | 5.445 | 0.84 | -0.2507 | 0.01701 | 0.06228 | no |
| BLZF1 | 9.445 | 9.442 | 7.796 | 7.986 | 9.444 | 7.891 | 0.84 | -0.2512 | 0.01595 | 0.05914 | no |
| AVL9 | 8.437 | 8.897 | 7.2 | 7.427 | 8.667 | 7.313 | 0.84 | -0.2513 | 0.003493 | 0.01691 | no |
| ATPAF1 | 32.061 | 30.33 | 26.72 | 26.028 | 31.195 | 26.374 | 0.84 | -0.2508 | 0.00008118 | 0.0006642 | no |
| AKIRIN2 | 13.026 | 10.999 | 10.522 | 9.636 | 12.012 | 10.079 | 0.84 | -0.2521 | 0.01566 | 0.05824 | no |
| TMEM39B | 5.978 | 5.83 | 4.299 | 5.088 | 5.904 | 4.694 | 0.841 | -0.2502 | 0.1203 | 0.2722 | no |
| SNIP1 | 5.237 | 4.37 | 3.879 | 4.258 | 4.803 | 4.069 | 0.841 | -0.2495 | 0.02568 | 0.08571 | no |
| SMN1 | 40.92 | 36.384 | 31.341 | 32.965 | 38.652 | 32.153 | 0.841 | -0.2498 | 0.01079 | 0.04344 | no |
| SEC24D | 15.29 | 15.661 | 13.179 | 12.984 | 15.476 | 13.082 | 0.841 | -0.2501 | 0.00133 | 0.007465 | no |
| RNF20 | 7.429 | 6.434 | 5.862 | 5.817 | 6.931 | 5.84 | 0.841 | -0.2506 | 0.006315 | 0.0278 | no |
| RABEPK | 12.069 | 10.668 | 8.832 | 9.456 | 11.369 | 9.144 | 0.841 | -0.2504 | 0.1104 | 0.2564 | no |
| PDSS1 | 2.377 | 2.015 | 1.514 | 1.589 | 2.196 | 1.551 | 0.841 | -0.2505 | 0.1977 | 0.3828 | no |
| NUDT19 | 7.491 | 7.232 | 5.95 | 6.137 | 7.361 | 6.043 | 0.841 | -0.2505 | 0.1071 | 0.2507 | no |
| NRDE2 | 3.385 | 3.066 | 2.696 | 2.659 | 3.225 | 2.678 | 0.841 | -0.2496 | 0.05962 | 0.1633 | no |
| MTRR | 11.164 | 10.921 | 9.35 | 9.166 | 11.043 | 9.258 | 0.841 | -0.2495 | 0.006481 | 0.02842 | no |
| MOB2 | 18.428 | 17.083 | 12.935 | 14.283 | 17.755 | 13.609 | 0.841 | -0.2496 | 0.1487 | 0.3151 | no |
| IMMP1L | 7.491 | 7.174 | 6.262 | 6.177 | 7.332 | 6.22 | 0.841 | -0.2503 | 0.07093 | 0.1859 | no |
| GPATCH2L | 7.737 | 8.186 | 6.438 | 6.367 | 7.962 | 6.402 | 0.841 | -0.249 | 0.001402 | 0.00781 | no |
| CNOT10 | 21.021 | 20.003 | 17.449 | 17.142 | 20.512 | 17.296 | 0.841 | -0.2493 | 0.003486 | 0.01688 | no |
| CHMP7 | 23.861 | 22.31 | 20.409 | 18.412 | 23.085 | 19.41 | 0.841 | -0.2505 | 0.002473 | 0.01263 | no |
| B3GALNT2 | 174.998 | 174.33 | 143.78 | 148.172 | 174.664 | 145.976 | 0.841 | -0.2491 | 0.00000565 | 0.00006167 | no |
| AP5S1 | 8.283 | 7.368 | 6.165 | 6.127 | 7.825 | 6.146 | 0.841 | -0.2497 | 0.0588 | 0.1617 | no |
| ZNF212 | 8.735 | 7.904 | 6.682 | 7.117 | 8.319 | 6.899 | 0.842 | -0.2484 | 0.03383 | 0.1061 | no |
| WASHC4 | 23.007 | 22.962 | 19.354 | 19.711 | 22.985 | 19.532 | 0.842 | -0.2487 | 0.00001762 | 0.0001686 | no |
| UTP20 | 16.977 | 16.693 | 14.244 | 14.423 | 16.835 | 14.334 | 0.842 | -0.2479 | 6.891E-07 | 0.000009221 | no |
| TARS | 80.132 | 78.736 | 66.355 | 68.429 | 79.434 | 67.392 | 0.842 | -0.2482 | 3.228E-07 | 0.000004598 | no |
| SCAF4 | 7.727 | 8.05 | 6.565 | 6.767 | 7.889 | 6.666 | 0.842 | -0.2477 | 0.006543 | 0.02865 | no |
| RBP4 | 282.777 | 231.116 | 212.704 | 212.572 | 256.947 | 212.638 | 0.842 | -0.2474 | 0.0001569 | 0.001184 | no |
| RBBP8 | 8.921 | 8.283 | 7.142 | 7.377 | 8.602 | 7.26 | 0.842 | -0.2483 | 0.004552 | 0.02101 | no |
| PLRG1 | 17.008 | 17.248 | 14.293 | 14.763 | 17.128 | 14.528 | 0.842 | -0.2489 | 0.002066 | 0.01088 | no |
| MYBL2 | 60.294 | 51.842 | 47.275 | 47.448 | 56.068 | 47.361 | 0.842 | -0.2477 | 0.0001082 | 0.0008556 | no |
| MISP | 43.513 | 39.684 | 34.614 | 35.524 | 41.599 | 35.069 | 0.842 | -0.2484 | 1.815E-07 | 0.000002728 | no |
| MCPH1 | 11.081 | 10.045 | 8.822 | 8.706 | 10.563 | 8.764 | 0.842 | -0.2474 | 0.01529 | 0.05719 | no |
| LPP | 6.318 | 5.023 | 4.074 | 7.047 | 5.67 | 5.56 | 0.842 | -0.2487 | 0.0004764 | 0.003122 | no |
| KIN | 2.377 | 2.599 | 1.846 | 2.119 | 2.488 | 1.983 | 0.842 | -0.2479 | 0.1564 | 0.326 | no |
| JMJD8 | 54.954 | 45.631 | 40.349 | 43 | 50.293 | 41.674 | 0.842 | -0.2489 | 0.0149 | 0.05611 | no |
| GALE | 16.957 | 15.603 | 13.922 | 13.284 | 16.28 | 13.603 | 0.842 | -0.2481 | 0.003388 | 0.01652 | no |
| EPS8 | 55.798 | 55.151 | 45.165 | 49.047 | 55.475 | 47.106 | 0.842 | -0.2475 | 0.000001585 | 0.00001969 | no |
| ZNF250 | 8.519 | 7.962 | 6.477 | 7.397 | 8.241 | 6.937 | 0.843 | -0.246 | 0.009277 | 0.0384 | no |
| USP2 | 3.138 | 3.728 | 2.56 | 2.859 | 3.433 | 2.71 | 0.843 | -0.2467 | 0.1188 | 0.2698 | no |
| TCERG1 | 36.65 | 36.793 | 30.227 | 31.795 | 36.721 | 31.011 | 0.843 | -0.2463 | 0.000007361 | 0.00007772 | no |
| SFXN1 | 62.208 | 58.684 | 50.548 | 50.907 | 60.446 | 50.727 | 0.843 | -0.246 | 1.804E-07 | 0.000002718 | no |
| MOB3C | 11.668 | 9.266 | 9.32 | 8.236 | 10.467 | 8.778 | 0.843 | -0.2468 | 0.03436 | 0.1073 | no |
| MIS12 | 35.765 | 38.701 | 29.084 | 26.808 | 37.233 | 27.946 | 0.843 | -0.2462 | 0.007961 | 0.03381 | no |
| METTL6 | 6.719 | 6.453 | 5.461 | 5.318 | 6.586 | 5.389 | 0.843 | -0.2468 | 0.1019 | 0.2413 | no |
| MED15 | 27.215 | 25.006 | 21.708 | 22.34 | 26.111 | 22.024 | 0.843 | -0.2471 | 0.002186 | 0.01144 | no |
| IRX1 | 3.221 | 2.307 | 1.759 | 0.56 | 2.764 | 1.159 | 0.843 | -0.2469 | 0.119 | 0.2699 | no |
| CDC16 | 19.23 | 18.883 | 15.172 | 16.862 | 19.056 | 16.017 | 0.843 | -0.2459 | 0.01356 | 0.05207 | no |
| BDP1 | 5.978 | 6.629 | 5.276 | 5.418 | 6.303 | 5.347 | 0.843 | -0.2466 | 0.003354 | 0.0164 | no |
| ZNF593 | 83.898 | 79.631 | 67.831 | 65.31 | 81.764 | 66.571 | 0.844 | -0.2443 | 0.02137 | 0.07455 | no |
| TARSL2 | 2.13 | 2.531 | 1.983 | 1.769 | 2.33 | 1.876 | 0.844 | -0.2442 | 0.1516 | 0.3194 | no |
| STX18 | 11.75 | 11.116 | 9.711 | 9.496 | 11.433 | 9.604 | 0.844 | -0.2443 | 0.01154 | 0.04584 | no |
| RBM5 | 22.194 | 20.538 | 18.562 | 18.491 | 21.366 | 18.526 | 0.844 | -0.244 | 0.0006779 | 0.004227 | no |
| PLAA | 15.228 | 14.308 | 12.486 | 12.524 | 14.768 | 12.505 | 0.844 | -0.2451 | 0.003639 | 0.01751 | no |
| MYNN | 2.984 | 2.745 | 2.599 | 2.239 | 2.865 | 2.419 | 0.844 | -0.2455 | 0.08603 | 0.2143 | no |
| GPAT4 | 26.731 | 24.655 | 21.435 | 22.45 | 25.693 | 21.942 | 0.844 | -0.2439 | 0.000008094 | 0.0000845 | no |
| GEMIN6 | 21.515 | 22.475 | 16.98 | 18.991 | 21.995 | 17.986 | 0.844 | -0.2452 | 0.07605 | 0.1962 | no |
| GCC1 | 5.422 | 4.302 | 3.8 | 4.278 | 4.862 | 4.039 | 0.844 | -0.2455 | 0.0631 | 0.1698 | no |
| DISP1 | 13.901 | 13.559 | 11.86 | 10.455 | 13.73 | 11.157 | 0.844 | -0.245 | 0.004345 | 0.02025 | no |
| ZNF653 | 10.598 | 9.636 | 8.646 | 8.356 | 10.117 | 8.501 | 0.845 | -0.2431 | 0.01001 | 0.04076 | no |
| UQCC1 | 9.713 | 8.4 | 7.327 | 8.036 | 9.056 | 7.681 | 0.845 | -0.2435 | 0.0211 | 0.07385 | no |
| UBE2J2 | 14.981 | 13.588 | 12.407 | 11.615 | 14.284 | 12.011 | 0.845 | -0.2428 | 0.01744 | 0.06346 | no |
| TPR | 30.044 | 30.476 | 25.626 | 26.008 | 30.26 | 25.817 | 0.845 | -0.2424 | 2.463E-07 | 0.000003617 | no |
| TAPBP | 9.168 | 5.918 | 6.428 | 5.907 | 7.543 | 6.168 | 0.845 | -0.2432 | 0.1063 | 0.2492 | no |
| STK17A | 5.752 | 5.422 | 4.709 | 4.718 | 5.587 | 4.713 | 0.845 | -0.2433 | 0.04094 | 0.1228 | no |
| SRC | 11.843 | 10.717 | 9.447 | 9.626 | 11.28 | 9.537 | 0.845 | -0.2433 | 0.006994 | 0.03023 | no |
| SMARCB1 | 59.934 | 58.402 | 47.695 | 52.136 | 59.168 | 49.916 | 0.845 | -0.2428 | 0.0007662 | 0.004682 | no |
| PLEKHN1 | 24.488 | 22.261 | 19.031 | 20.531 | 23.374 | 19.781 | 0.845 | -0.2428 | 0.003684 | 0.01771 | no |
| MRPL9 | 67.199 | 63.999 | 53.723 | 56.814 | 65.599 | 55.269 | 0.845 | -0.2429 | 0.0009109 | 0.00545 | no |
| KCNS3 | 5.494 | 6.084 | 4.484 | 4.708 | 5.789 | 4.596 | 0.845 | -0.2434 | 0.1661 | 0.3397 | no |
| ICE1 | 11.812 | 11.028 | 9.721 | 9.736 | 11.42 | 9.729 | 0.845 | -0.2422 | 0.0003248 | 0.00223 | no |
| EPB41L5 | 5.186 | 4.964 | 4.426 | 4.138 | 5.075 | 4.282 | 0.845 | -0.2426 | 0.01922 | 0.06847 | no |
| ENDOU | 8.591 | 7.709 | 6.575 | 6.957 | 8.15 | 6.766 | 0.845 | -0.2426 | 0.06803 | 0.1799 | no |
| EED | 14.724 | 14.143 | 12.134 | 12.204 | 14.434 | 12.169 | 0.845 | -0.2426 | 0.02387 | 0.08103 | no |
| DNAJC25 | 88.898 | 76.575 | 72.032 | 71.437 | 82.737 | 71.734 | 0.845 | -0.2432 | 0.0003553 | 0.002419 | no |
| CHRNA5 | 2.294 | 2.657 | 2.227 | 1.659 | 2.476 | 1.943 | 0.845 | -0.2427 | 0.1523 | 0.3204 | no |
| C7orf26 | 43.842 | 39.859 | 35.923 | 34.844 | 41.85 | 35.383 | 0.845 | -0.2425 | 0.001909 | 0.01013 | no |
| USP16 | 28.511 | 27.809 | 24.043 | 23.929 | 28.16 | 23.986 | 0.846 | -0.2406 | 0.0006852 | 0.004262 | no |
| TNIP2 | 8.087 | 8.186 | 6.819 | 6.557 | 8.136 | 6.688 | 0.846 | -0.2417 | 0.07446 | 0.193 | no |
| SUPT4H1 | 18.932 | 18.328 | 15.807 | 15.743 | 18.63 | 15.775 | 0.846 | -0.2407 | 0.006355 | 0.02791 | no |
| SPATA1 | 5.206 | 4.205 | 4.084 | 3.468 | 4.706 | 3.776 | 0.846 | -0.2421 | 0.1561 | 0.3257 | no |
| SLC30A7 | 13.119 | 12.663 | 11.001 | 11.005 | 12.891 | 11.003 | 0.846 | -0.2413 | 0.0000885 | 0.0007169 | no |
| SH3GL1 | 24.231 | 21.336 | 19.852 | 18.521 | 22.784 | 19.187 | 0.846 | -0.242 | 0.00963 | 0.03947 | no |
| RCHY1 | 15.609 | 12.732 | 11.587 | 11.835 | 14.171 | 11.711 | 0.846 | -0.2417 | 0.07155 | 0.187 | no |
| RCC1L | 25.435 | 23.993 | 21.454 | 20.101 | 24.714 | 20.777 | 0.846 | -0.2414 | 0.0005442 | 0.003508 | no |
| RBM15 | 2.819 | 2.881 | 2.462 | 2.289 | 2.85 | 2.376 | 0.846 | -0.2415 | 0.1349 | 0.2948 | no |
| PSMD7 | 97.315 | 86.026 | 79.212 | 75.655 | 91.671 | 77.434 | 0.846 | -0.2418 | 0.0001399 | 0.001069 | no |
| PPP4R3B | 37.113 | 33.98 | 30.247 | 30.396 | 35.546 | 30.322 | 0.846 | -0.2417 | 0.000002971 | 0.0000351 | no |
| NCDN | 19.982 | 18.942 | 16.687 | 16.352 | 19.462 | 16.52 | 0.846 | -0.2414 | 0.001975 | 0.01045 | no |
| MED28 | 32.205 | 34.282 | 30.149 | 28.467 | 33.243 | 29.308 | 0.846 | -0.2419 | 0.002243 | 0.01166 | no |
| LIN9 | 18.13 | 17.569 | 14.889 | 14.883 | 17.849 | 14.886 | 0.846 | -0.2409 | 0.03294 | 0.1041 | no |
| KIAA1671 | 9.034 | 9.607 | 7.992 | 8.026 | 9.32 | 8.009 | 0.846 | -0.2407 | 0.003574 | 0.01724 | no |
| DNAJC19 | 5.525 | 4.906 | 4.172 | 4.178 | 5.216 | 4.175 | 0.846 | -0.2413 | 0.09435 | 0.2281 | no |
| DDX20 | 12.347 | 12.05 | 10.571 | 10.055 | 12.198 | 10.313 | 0.846 | -0.2408 | 0.01204 | 0.04726 | no |
| DCAF1 | 15.177 | 14.63 | 12.476 | 12.834 | 14.904 | 12.655 | 0.846 | -0.241 | 0.0002709 | 0.001919 | no |
| CPTP | 23.789 | 19.117 | 19.354 | 17.812 | 21.453 | 18.583 | 0.846 | -0.2417 | 0.01949 | 0.06925 | no |
| CLDN10 | 1.101 | 3.193 | 1.055 | 0.85 | 2.147 | 0.952 | 0.846 | -0.2412 | 0.1352 | 0.2954 | no |
| CDC23 | 13.592 | 12.693 | 10.698 | 11.475 | 13.143 | 11.087 | 0.846 | -0.2411 | 0.01194 | 0.04693 | no |
| CASP8 | 16.524 | 15.837 | 13.873 | 13.594 | 16.181 | 13.733 | 0.846 | -0.2412 | 0.004066 | 0.01918 | no |
| C18orf8 | 24.426 | 21.531 | 20.301 | 18.292 | 22.978 | 19.297 | 0.846 | -0.2409 | 0.01638 | 0.06037 | no |
| BOP1 | 85.081 | 69.849 | 64.382 | 66.989 | 77.465 | 65.686 | 0.846 | -0.2421 | 0.0002823 | 0.001984 | no |
| APCDD1 | 19.519 | 18.552 | 15.983 | 16.263 | 19.035 | 16.123 | 0.846 | -0.2409 | 0.001019 | 0.005985 | no |
| AP5B1 | 10.588 | 9.928 | 8.431 | 8.806 | 10.258 | 8.618 | 0.846 | -0.2406 | 0.02248 | 0.07741 | no |
| STX12 | 16.463 | 15.652 | 13.648 | 13.554 | 16.058 | 13.601 | 0.847 | -0.2389 | 0.009961 | 0.04062 | no |
| SRPK1 | 20.105 | 19 | 16.843 | 16.492 | 19.553 | 16.668 | 0.847 | -0.2391 | 0.0006112 | 0.003862 | no |
| RAD51D | 4.383 | 4.799 | 3.879 | 3.838 | 4.591 | 3.859 | 0.847 | -0.2399 | 0.03252 | 0.1029 | no |
| RAD23B | 55.068 | 56.056 | 47.148 | 47.838 | 55.562 | 47.493 | 0.847 | -0.2394 | 0.000001798 | 0.0000222 | no |
| PITX1 | 4.435 | 4.03 | 3.556 | 3.149 | 4.232 | 3.353 | 0.847 | -0.2398 | 0.1769 | 0.355 | no |
| PHYH | 23.212 | 22.952 | 19.705 | 18.921 | 23.082 | 19.313 | 0.847 | -0.2401 | 0.02163 | 0.07529 | no |
| DNAJB1 | 24.859 | 24.305 | 20.477 | 21.55 | 24.582 | 21.014 | 0.847 | -0.2404 | 0.0002378 | 0.001708 | no |
| DCAF15 | 12.378 | 11.963 | 10.346 | 11.595 | 12.171 | 10.971 | 0.847 | -0.2401 | 0.02229 | 0.07679 | no |
| ZBED4 | 15.002 | 13.501 | 12.798 | 12.294 | 14.252 | 12.546 | 0.848 | -0.2385 | 0.006793 | 0.02953 | no |
| USP38 | 3.416 | 3.183 | 2.648 | 2.799 | 3.3 | 2.724 | 0.848 | -0.2384 | 0.03798 | 0.1162 | no |
| TIPRL | 7.892 | 7.612 | 6.223 | 6.867 | 7.752 | 6.545 | 0.848 | -0.2385 | 0.03137 | 0.1001 | no |
| RUNDC1 | 11.616 | 9.364 | 8.285 | 9.276 | 10.49 | 8.78 | 0.848 | -0.2371 | 0.043 | 0.1273 | no |
| RAVER1 | 40.498 | 36.443 | 32.748 | 33.005 | 38.471 | 32.877 | 0.848 | -0.2383 | 0.00000965 | 0.00009889 | no |
| RALBP1 | 22.739 | 22.271 | 19.1 | 19.301 | 22.505 | 19.2 | 0.848 | -0.2387 | 0.0004756 | 0.00312 | no |
| PTK2 | 40.951 | 40.327 | 33.52 | 34.684 | 40.639 | 34.102 | 0.848 | -0.2378 | 0.000004856 | 0.00005393 | no |
| PPP6R2 | 30.723 | 30.895 | 25.645 | 26.408 | 30.809 | 26.026 | 0.848 | -0.2378 | 0.000122 | 0.0009513 | no |
| KHSRP | 55.623 | 53.496 | 47.178 | 46.219 | 54.559 | 46.698 | 0.848 | -0.238 | 5.874E-07 | 0.00000799 | no |
| GNE | 17.728 | 17.336 | 15.045 | 14.913 | 17.532 | 14.979 | 0.848 | -0.2378 | 0.00119 | 0.006819 | no |
| FBXL2 | 15.65 | 15.36 | 13.463 | 12.304 | 15.505 | 12.883 | 0.848 | -0.2372 | 0.0269 | 0.08893 | no |
| COG3 | 9.25 | 8.965 | 7.738 | 7.726 | 9.107 | 7.732 | 0.848 | -0.2384 | 0.01019 | 0.04138 | no |
| WIPF2 | 13.366 | 12.401 | 10.62 | 11.325 | 12.883 | 10.973 | 0.849 | -0.2357 | 0.0007945 | 0.004832 | no |
| TGIF2-C20orf24 | 111.699 | 111.061 | 94.121 | 93.837 | 111.38 | 93.979 | 0.849 | -0.2354 | 0.000542 | 0.003497 | no |
| MRPS35 | 59.903 | 58.957 | 49.219 | 49.687 | 59.43 | 49.453 | 0.849 | -0.2367 | 0.004149 | 0.01949 | no |
| METAP2 | 39.15 | 37.543 | 31.82 | 34.174 | 38.346 | 32.997 | 0.849 | -0.2366 | 1.104E-07 | 0.000001713 | no |
| ING5 | 5.834 | 4.779 | 4.836 | 3.798 | 5.306 | 4.317 | 0.849 | -0.2362 | 0.08195 | 0.207 | no |
| IGF2BP1 | 17.008 | 15.885 | 14.156 | 13.964 | 16.447 | 14.06 | 0.849 | -0.2366 | 0.00005182 | 0.0004452 | no |
| FAM160A2 | 4.095 | 4.088 | 3.644 | 3.089 | 4.091 | 3.367 | 0.849 | -0.2362 | 0.1113 | 0.2578 | no |
| FAM13B | 4.27 | 4.701 | 3.449 | 4.018 | 4.486 | 3.733 | 0.849 | -0.2366 | 0.06013 | 0.1641 | no |
| DCP1A | 6.883 | 6.58 | 5.754 | 5.607 | 6.732 | 5.681 | 0.849 | -0.2367 | 0.03301 | 0.1042 | no |
| CNOT7 | 27.698 | 27.254 | 23.848 | 22.989 | 27.476 | 23.419 | 0.849 | -0.2357 | 0.001803 | 0.009635 | no |
| CDCA5 | 32.637 | 28.568 | 25.616 | 26.438 | 30.602 | 26.027 | 0.849 | -0.2363 | 0.002234 | 0.01163 | no |
| ARID1B | 12.09 | 12.42 | 10.424 | 10.595 | 12.255 | 10.509 | 0.849 | -0.2367 | 0.0003417 | 0.002331 | no |
| ANGPTL7 | 0.494 | 0.837 | 0.322 | 0.44 | 0.665 | 0.381 | 0.849 | -0.2362 | 0.1826 | 0.3624 | no |
| ZSCAN12 | 8.077 | 8.05 | 6.194 | 7.377 | 8.064 | 6.785 | 0.85 | -0.2341 | 0.01464 | 0.05525 | no |
| VPS37A | 9.991 | 8.809 | 8.089 | 7.946 | 9.4 | 8.018 | 0.85 | -0.2353 | 0.03022 | 0.09723 | no |
| SNX8 | 12.831 | 12.537 | 10.981 | 10.605 | 12.684 | 10.793 | 0.85 | -0.2349 | 0.006438 | 0.02824 | no |
| SNX5 | 50.828 | 50.479 | 42.674 | 45.099 | 50.654 | 43.886 | 0.85 | -0.2348 | 0.00007445 | 0.0006161 | no |
| PALLD | 4.774 | 5.139 | 4.054 | 4.248 | 4.957 | 4.151 | 0.85 | -0.2351 | 0.08478 | 0.212 | no |
| NUP93 | 32.678 | 33.26 | 27.248 | 29.077 | 32.969 | 28.163 | 0.85 | -0.2348 | 0.001063 | 0.006189 | no |
| MAPKAPK5 | 7.326 | 6.093 | 5.569 | 5.777 | 6.71 | 5.673 | 0.85 | -0.2339 | 0.03865 | 0.1177 | no |
| HDGFL2 | 83.373 | 71.912 | 61.705 | 64.081 | 77.643 | 62.893 | 0.85 | -0.2345 | 0.0003315 | 0.00227 | no |
| GRPEL1 | 45.087 | 39.11 | 36.128 | 35.384 | 42.099 | 35.756 | 0.85 | -0.2345 | 0.001564 | 0.008597 | no |
| GNA11 | 12.542 | 11.641 | 10.463 | 10.025 | 12.091 | 10.244 | 0.85 | -0.2342 | 0.02121 | 0.07412 | no |
| GATA6 | 10.927 | 6.21 | 1.798 | 2.129 | 8.569 | 1.964 | 0.85 | -0.235 | 0.1789 | 0.3578 | no |
| FRMD6 | 38.193 | 37.163 | 31.81 | 32.745 | 37.678 | 32.277 | 0.85 | -0.235 | 0.000003989 | 0.00004535 | no |
| ARL4C | 19.765 | 10.172 | 10.942 | 10.105 | 14.969 | 10.524 | 0.85 | -0.2345 | 0.2266 | 0.4197 | no |

^a^Raw read counts have been normalized by the principle of “reads per kilo bases per million reads”.

^b^Fold change between average read counts from FCV infection group and mock group.

^C^Statistics analysis is based on DESeq2 software (p-adjust < 0.05 && |log_2_FC| >= 1) and EdgeR software is also used for differentially expressed analysis (p-adjust < 0.05 && |log_2_FC| >= 1).
